# Genes of the most conserved WOX clade in plants affect root and flower development in Arabidopsis

**DOI:** 10.1186/1471-2148-8-291

**Published:** 2008-10-24

**Authors:** Yves Deveaux, Claire Toffano-Nioche, Gaelle Claisse, Vincent Thareau, Halima Morin, Patrick Laufs, Hervé Moreau, Martin Kreis, Alain Lecharny

**Affiliations:** 1 Université Paris-Sud 11, Institut de Biotechnologie des Plantes, Bâtiment 630, UMR/CNRS 8618, F-91405 Orsay, France; 2 Laboratoire de Biologie Cellulaire, Institut J. P. Bourgin, INRA, 78026 Versailles Cedex, France; 3 Observatoire Océanologique, Laboratoire Arago, Unité Mixte de Recherche 7628, CNRS-Université Pierre et Marie Curie, BP44, 66651 Banyuls sur Mer Cedex, France

## Abstract

**Background:**

The Wuschel related homeobox (WOX) family proteins are key regulators implicated in the determination of cell fate in plants by preventing cell differentiation. A recent WOX phylogeny, based on WOX homeodomains, showed that all of the *Physcomitrella patens *and *Selaginella moellendorffii *WOX proteins clustered into a single orthologous group. We hypothesized that members of this group might preferentially share a significant part of their function in phylogenetically distant organisms. Hence, we first validated the limits of the WOX13 orthologous group (WOX13 OG) using the occurrence of other clade specific signatures and conserved intron insertion sites. Secondly, a functional analysis using expression data and mutants was undertaken.

**Results:**

The WOX13 OG contained the most conserved plant WOX proteins including the only WOX detected in the highly proliferating basal unicellular and photosynthetic organism *Ostreococcus tauri*. A large expansion of the WOX family was observed after the separation of mosses from other land plants and before monocots and dicots have arisen. In *Arabidopsis thaliana*, *AtWOX13 *was dynamically expressed during primary and lateral root initiation and development, in gynoecium and during embryo development. *AtWOX13 *appeared to affect the floral transition. An intriguing clade, represented by the functional *AtWOX14 *gene inside the WOX13 OG, was only found in the Brassicaceae. Compared to *AtWOX13*, the gene expression profile of *AtWOX14 *was restricted to the early stages of lateral root formation and specific to developing anthers. A mutational insertion upstream of the *AtWOX14 *homeodomain sequence led to abnormal root development, a delay in the floral transition and premature anther differentiation.

**Conclusion:**

Our data provide evidence in favor of the WOX13 OG as the clade containing the most conserved WOX genes and established a functional link to organ initiation and development in Arabidopsis, most likely by preventing premature differentiation. The future use of *Ostreococcus tauri *and *Physcomitrella patens *as biological models should allow us to obtain a better insight into the functional importance of WOX13 OG genes.

## Background

Homeodomain (HD) containing transcription factors are key regulators implicated in the determination of cell fate and cell differentiation in both plants and animals. In Angiosperms, a gene called *WUSCHEL *(*WUS*) has been isolated from many different species. *WUS *was the first identified member of the Wuschel-related homeobox (*WOX*) subfamily [1,2] that is only found in plants.

The specific expression of the *WOX *genes in different plant organs and cell types [2-7] suggested an important role for them during organogenesis. The *WUS *gene is expressed in a restricted region of the meristem called the organizing centre located below the stem cells of the shoot apical meristem (SAM) and functions non-cell autonomously to control the stem cell fate [8]. Interestingly Gallois et al [3] showed recently that ectopic expression of WUS in the root establishes shoot stem cells and leaf development. Other members of the WOX family might also prevent premature cell differentiation in developing organs or tissues. Recently, WOX5 was shown to be involved in stem cell maintenance signaling in the root [9]. Wu et al [10] have shown that WOX9 (Stimpy/STIP) prevents premature cell differentiation during organ growth, in addition to contributing positively to *WUS *expression. This work also revealed that *stip *mutants can be rescued by sucrose, a modulator of cell proliferation trough *cyclin D *induction [11,12]. Two other *WOX *genes, named Narrow Sheath 1 and 2, that are maize orthologues of *Arabidopsis thaliana AtWOX6 *were shown to be involved in the recruitment of the lateral founder cells within the SAM prior to the primordia development [13]. Last, Haecker et al [1] analyzed the expression dynamics of the *WOX *genes during *A. thaliana *embryo development and showed that members of the *WOX *family mark cell fate decisions during embryonic patterning. Taken together, these results suggest that not only *WUS *but several other *WOX *genes play a role in regulating cell division and preventing cell differentiation.

Characterized genomes of Angiosperms contain more than 10 different *WOXs *and phylogenetic studies suggest that the *WOX *family existed in the last common ancestor of monocots and core eudicots [14,15]. A recent WOX phylogeny based on WOX HDs from different Angiosperms, the moss *Physcomitrella patens *and the lycophyte *Selaginella moellendorffii *showed that all of the *P. patens *and eight of the *S. moellendorffii WOX *genes clustered in the same clade [16]. Indeed, the phylogenetic analyses of a transcription factor family like WOX suffer from some limitations due to the relatively short length of the HD, which is the only conserved sequence between the WOX protein family members, and the relatively few land plant species with a completely characterized proteome. As a consequence, the patterns of phylogenic trees of large families of plant transcription factors are often weakly supported by statistics and may be affected by long branch attraction [17]. Nevertheless, clade specific gene features and protein domains were observed in transcription factors [18,19]. Thus, in this study, we used clade specific signatures beyond the HD and clade specific conservation of intron insertion sites, to provide additional data in support of the orthology links predicted by the WOX phylogeny. In order to carry out the latter analyses, complete genome sequences with accurate structural annotations are essential. Our results strongly support the existence of a WOX13 orthologous group (WOX13 OG) that, contrary to the two other WOX OGs, contains genes from many different members of the plant kingdom, including basal species, and a branch apparently specific to Brassicaceae. To obtain information on the function of the genes belonging to the WOX13 clade, the cell specificity of At *WOX13 *and At *WOX14 *expression was established experimentally and phenotypes resulting from mutations within these two genes were characterized in the plant model *A. thaliana*.

## Results

### A WOX distance tree from model genomes

We carefully screened several completely sequenced eukaryote genomes for WOX genes, *i.e.*, two Angiosperms, *A. thaliana *and *O. sativa*, three algae, *O. tauri*, *O. lucimarinus *and *Chlamydomonas reinhardtii *and two yeasts, *Saccharomyces cerevisiae *and *Schizosaccharomyces pombe*. No *WOX *genes were identified outside of the plant kingdom and *Ostreococcus *revealed only one WOX gene per genome. Moreover, using the HMMsearch tool [20], we found only one HD gene in *C. reinhardtii*, 2 in *S. pombe*, 4 in *O. tauri *and 8 in *S. cerevisiae *compared to the 92 HD genes in *A. thaliana*.

**Figure 1:**
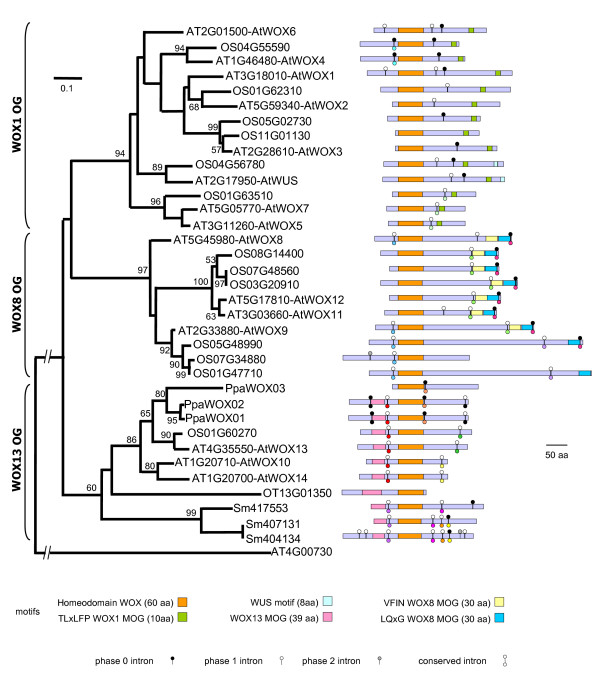
***WOX *genes from model plant genomes**. The neighbor-joining tree of the WOX proteins from the 5 completely sequenced genomes of the green lineage: *Arabidopsis thaliana *(AT prefix), *Oryza sativa *(OS prefix), *Physcomitrella patens *(Ppa prefix), *Ostreococcus tauri *(OT prefix) and *Selaginella moellendorffii *(Sm prefix). The tree topology was inferred from Jones-Taylor-Thornton matrix distances. The significance of each node was tested using 1000 bootstrap replicates. Only bootstrap values above 50% are shown. The scale, at the top left of the tree, indicates 0.1 substitutions per site. Different structural features are represented by different signs and colors as described at the bottom of the figure.

Distance and phylogenic trees were computed from the HD sequences of the WOX proteins from the five plant model genomes, *O. tauri*, *P. patens, S. moellendorffii*, *A. thaliana *and *O. sativa *(see method section). The clustering of the WOX proteins in 3 different OGs were consistent using either the Neighbor-Joining (Figure 1) or the Maximum likelihood (see additional file 1: Figure 1) methods and different outgroups. The topology of the trees clearly showed three different OGs namely the WOX1, WOX8 and WOX13 MOGs (for Model plant OG). Each OG contained rice and Arabidopsis proteins, but the single WOX protein of *O. tauri*, the three *P. patens *and the three *S. Moellendorffii *WOX proteins were all found in the same group, the WOX13 MOG. The WOX1 MOG contained AtWOX1-7 and AtWUS, the WOX8 OG associated AtWOX8, 9, 11 and 12 and the WOX13 OG included AtWOX10, 13 and 14. Thus, the previous observation of the presence of the *P. patens *proteins only in the WOX13 OG group was extended to *O. tauri *and *S. moellendorffii*. However, the introduction of OtWOX and the SmWOXs did not change the main branching of the trees nor significantly modified the bootstrap values at the internal node of the tree. The 14 available *WOX *sequences from different Gymnosperms were distributed between the three OGs (data not shown). Furthermore, the trees we obtained were not consistent with the phylogenetic tree of plant species as it is now known [21]. Thus the WOX trees we produced suggest probably several rounds of gene deletions in *O. tauri *and *P. patens *in order to explain the branching of the WOX13 OG. The finding that all the WOX sequences of *O. tauri *and *P. patens*, as well as those of *S. moellendorffii *were clustered in the WOX13 OG suggested to us that *AtWOX10, 13, 14 *might represent the most interesting genes to study the functional diversification of the WOX genes although they are the WOX genes for which we have the least functional information (reviewed in [22]). This prompted us to look at the specificity of the WOX13 OG with regards to gene structure and conserved amino acid signatures other than the HD. Conservation in gene structure is an indication of a common ancestor while conserved amino acid signatures are linked to functions such as protein-protein interactions or regulatory sites.

### WOX gene structures

The intron-exon organization of *WOX *genes from the model genomes showed different gene structures (see additional file 2: Figure 1). Alignments supporting the conservation of intron insertion sites are given in additional file 2: Figure 2. The organization of the gene structures along the phylogeny tree did not reveal any contradiction between gene structures and tree pattern. Intron site conservations were exclusively observed within each of the three OGs and never between them. In this context, it was interesting to observe that the insertion site of the first intron in *AtWOX13 *is conserved in almost all the genes (from moss to Angiosperms) belonging to the WOX13 OG. The only exception was *PpaWOX03*, a possible pseudogene (see below). This observation cannot be extended to *O. tauri *as its genes do not have introns. *S. moellendorffii *genes have introns and the three full-length *SmWOXs *have one conserved intron insertion site downstream from the WOX13 motif. The latter intron of *SmWOXs *is in a similar position (relative to the coding region) to the first highly conserved intron of WOX13 OG genes in moss and Angiosperms. Nevertheless, there is no trace of amino acid sequence conservation in the corresponding protein region between *S. moellendorffii *and the other genes in the WOX13 OG (see additional file 3: Table 3). When taken together, our analyses of intron insertion sites support the boundaries of the WOX13 OG as defined by phylogeny. In addition to the highly conserved intron upstream of the HD coding sequence found in the WOX13 OG, *AtWOX10 *and *AtWOX14 *share another conserved intron insertion site downstream of the HD while *OS01G60270 *and *AtWOX13 *share a different intron insertion site in the 3' region of the gene. The tree pattern implies first a loss of the orthologues of *AtWOX10 *and *14 *in *O. sativa *and in *P. patens *and secondly that *AtWOX13 *and *OS01G60270 *are orthologous. Interestingly, the two latter genes share a specific intron insertion site.

### Specific motifs in the WOX proteins

A search for specific motifs other than the HD used to build the distance and phylogeny trees led us to identify HMMs that were specific to each clade and located either within the N or the C terminal regions of the different proteins. These HMMs are illustrated by sequence logos (see additional file 2: Figure 3) and by alignments (see additional file 2: Figure 4). Only proteins of the WOX1 clade have a small motif of 10 amino acids (*i.e.*, the TLxLFP motif) in the C-terminal region, but at relatively variable distances from the HD. TLxLFP has previously been described as a characteristic motif of WUS proteins [1,23], but we show here that it is common to the entire WOX1 clade, *i.e.*, it is observed not only in WUS, but also in the AtWOX1-7 proteins. However, the WUS proteins of *A. thaliana *(AT2G17950-AtWUS) and *O. sativa *(OS04G56780) share specific features not observed in any of the other proteins of the WOX1 MOG. Indeed, they display 1) the LELxL WUS motif [1] known as EAR-like or ERF-like motifs that are located in the outmost C-terminal region, similar to a motif found in potent transcriptional repressors in plants *i.e.*, Superman [24] and Aux/IAA [25]; 2) the same intron exon structure in the coding regions of each gene; and 3) an additional amino acid at a conserved position in the homeodomain.

Nearly all proteins of the WOX8 MOG contain two contiguous motifs of 30 amino acids situated near to their C-terminus (*i.e.*, VFIN-WOX8 and LQxG-WOX8 MOG motifs). The latter motifs are surrounded by two conserved introns. Remarkably, these introns are also found in OS05G48990 in which the VFIN-WOX8 MOG motif is replaced by a non-conserved sequence of a similar length.

All the proteins of the WOX13 MOG, except for PpaWOX03, share a 39 amino acid motif that is found upstream from the HD and named the WOX13 MOG motif. This observation provides further support for the validity of the WOX13 OG as defined by phylogeny and to the hypothesis of a specific function of the genes clustered within. For all of these reasons, it was decided to investigate further the WOX13 clade.

### WOX13 OG distance tree

**Figure 2:**
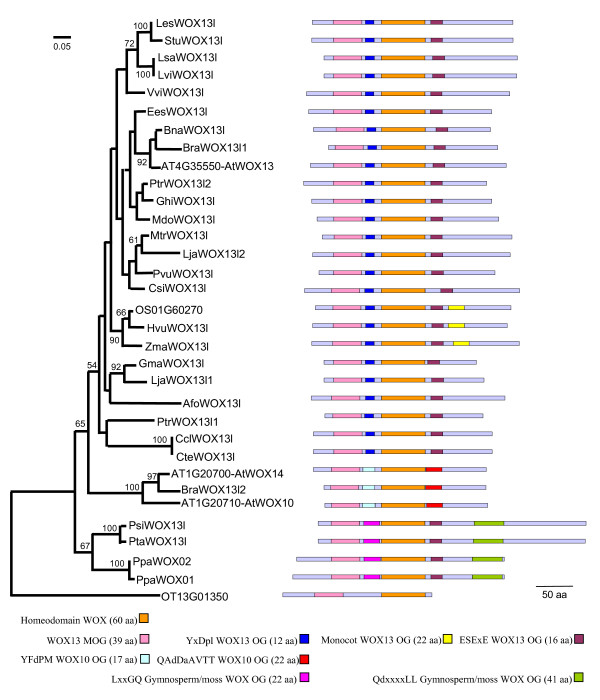
**The WOX13 Orthology Group**. The different elements of the figure are described from the left to the right. 1) The neighbor-joining tree of the WOX proteins from the WOX13 Orthology Group (see material and methods). The tree topology, the significance of the nodes, and the bootstrap values displayed are the same as in Figure 1. The scale at the top left corner indicates 0.05 substitutions per site. 2) The motif composition of each protein is denoted by colored boxes on a bar representing the length of the protein and aligned on the WOX homeodomain (displayed in orange). Species names, in alphabetic order, are: Afo: *Aquilegia formosa*; AT: *Arabidopsis thaliana*; Bna: *Brassica napus*; Bra: *Brassica rapa*; Ccl: *Citrus clementina*; Csi: *Citrus sinensis*; Cte: *Citrus temple*; Ees: *Euphorbia esula*; Ghi: *Gossypium hirsutum*; Gma: *Glycine max*; Gra: *Gossypium raimondii*; Han: *Helianthus annuus*; Hvu: *Hordeum vulgare*; Les: *Lycopersicon esculentum*; Lja: *Lotus japonicus*; Lsa: *Lactuca sativa*; Lvi: *Lactuca virosa*; Mdo: *Malus domestica*; Mtr: *Medicago truncatula*; OS: *Oryza sativa*; OT: *Ostreococcus tauri*; Ppa: *Physcomitrella patens*; Psi: *Picea sitchensis*; Pta: *Pinus taeda*; Ptr: *Populus tremula *× *Populus tremuloides*; Pvu: *Phaseolus vulgaris*; Stu: *Solanum tuberosum*; Vvi: *Vitis vinifera*; Zma: *Zea mays*. The color code of the different motifs is described at the bottom of the figure.

Thirty three full length WOX proteins were used to reconstruct the distance tree of the WOX13 clade (Figure 2), using OtWOX as the outgroup. The neighbor-joining tree showed two main branches. The first branch contained all of the Gymnosperm and the moss WOXs. The second branch contained AtWOX13 together with many Angiosperm sequences. This branch was further separated into two branches, one contained AtWOX13 and many proteins from different Angiosperms while the second contained AtWOX10, AtWOX14 and a *Brassica rapa *sequence.

The preferential presence of amino acid motifs fully supported the separation of the different gene groups within the trees. Obviously, all proteins of the WOX13 clade displayed the WOX HD and the WOX13 motif. Moreover, we also identified specific motifs for the AtWOX10, 14 and for the AtWOX13 branches (see additional file 2: Figure 3). All Angiosperm proteins located inside the WOX13 OG shared two motifs, the YxDpl-WOX13 motif located between the WOX13 MOG motif and the HD, and the ESExE-WOX13 motif just downstream from the HD. The monocots of the AtWOX13 branch also share a specific motif called the monocot-WOX13 motif that was detected also in two other partial proteins of two monocotyledon species.

Proteins of the AtWOX10, 14 branch shared two specific motifs, the YFdPM-WOX10 and QAdDaAVTT-WOX10 motifs. They were located to the same region of the protein as the YxDpl-WOX13 and ESExE-WOX13 motifs, respectively. Hence, the similarity between the YFdPM-WOX10 OG and YxDpl-WOX13 motifs suggested a common origin. Partial protein sequences from *Brassica oleracea *and *Raphanus raphanistrum subsp. raphanistrum *(protein sequence derived from the two ESTs, gb|EX762111| and gb|EX766460|) confirmed the specificity of the motifs to Brassicaceae. Thus, the AtWOX14 containing branch appears to be Brassicaceae specific since: 1) all the sequences containing the two AtWOX10, 14 motifs are from Brassicaceae and 2) no species other than those belonging to the Brassicaceae displayed any of these two motifs.

The Gymnosperm and moss branch contained proteins that shared the ESExE-WOX13 OG motif with the Angiosperm WOX13 proteins, located at the same position relative to the HD. They also shared two other motifs specific to this branch: 1) the LxxGQ-gymnosperm/moss-WOX OG motif that was at the same position as the YxDpl-WOX13 OG or YFdPM-WOX10 OG motifs and 2) a supplementary motif in the C-terminal region of the proteins.

### Functional WOX13 OG genes in the three model plants

We investigated the expression profiles of the WOX13 OG genes within 3 plant model species namely *O. tauri*, *P. patens *and *A. thaliana *because of their position within the green lineage. *O. tauri *is a highly proliferating unicellular alga that has no known differentiated form. In addition to the *WOX *gene, we also identified a single copy of a *KNOX-like *gene (*Ot04g04460*) within the *O. tauri *genome. In Angiosperms, members of the class-1 KNOX subfamily act to prevent cell differentiation within the SAM [26]. Interestingly, the OtKNOX protein showed the characteristics of both the class-1 and class-2 KNOX conserved domains, suggesting that *O. tauri *had diverged from a common ancestor before the diversification of the *KNOX *gene into 2 classes.

**Figure 3:**
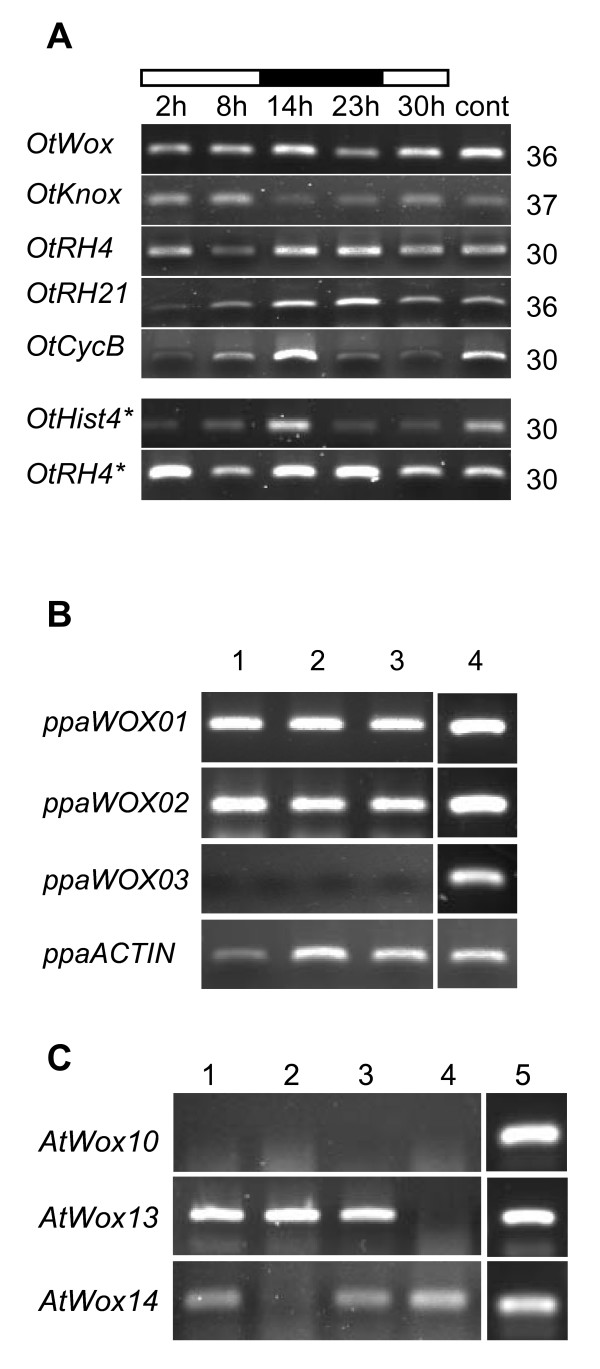
**WOX13 OG gene expression in *O. tauri, P. patens and A. thaliana***. **(A) **RT-PCR analysis of WOX gene expression in *Ostreococcus tauri*. 12 h light/dark synchronized or continuous light (cont) cell cultures of 10-day-old were used. Gene name (left side), number of PCR cycles done (right side), time of sampling (top side), light (blank bar) and dark (black bar) periods. The genes *OtRH4 *and *OtRH21 *were used as constitutive expression controls. RT were done using oligodT primers except in (*) where random primers were used **(B) **RT-PCR analysis of *WOX *gene expression during *Physcomitrella patens *development: (1) 4-day-old, (2) 10-day-old, (3) 14-day-old, (4) genomic DNA control. **(C) **RT-PCR analysis of the expression of WOX13 OG genes carried out on 7-day old seedlings of different *Arabidopsis thaliana *lines: (1) Wild type No ecotype, (2) the pst13645 *AtWOX14 *mutant line, (3) Wild type WS ecotype, (4) the 193G10 *AtWOX13 *mutant line, (5) Genomic DNA control.

In order to monitor the expression of *WOX *and *KNOX *genes during the progression of the cell cycle, we partially light-synchronized a cell suspension of *O. tauri *as described before in [27]. RT-PCR and quantitative PCR analyses of *Cyclin B *and *Histone 4 *genes showed a peak of expression at 14 h, confirming the overlapping of the G2/M and S subpopulations (Figure 3A and see additional file 4: Figure 1). The *OtRH4 (Ot07g03220)*, and *OtRH21 *(*Ot10g01950*) genes, respectively the orthologues of the Arabidopsis *DEAD-box RNA-Helicase 4 *and *21 *[28] were used as controls of constitutively expressed genes. They did not show any significant variation, and they were not cell cycle regulated (Figure 3A). More importantly, the *WOX *and the *KNOX *genes were found to be constitutively expressed and not cell cycle regulated (Figure 3A), with a higher expression level for *WOX *compared to *KNOX *(see additional file 4: Figure 1).

In *P. patens*, the initial gametophytic phase consists of an array of filamentous tubes that are formed after cell initials are produced from the main filament. Further development proceeds by the formation of buds which form the initial meristem from which originates the leafy adult gametophyte [29]. These processes rely on a tight control of cellular differentiation throughout the gametophyte development. An analysis of *WOX *expression in *P. patens *was carried out on 4-, 10- and 14-day-old plants corresponding respectively to the protonema, the bud and the gametophore stages under our growth conditions (data not shown). RT-PCR analyses showed that *PpaWOX01 *and *PpaWOX02 *were expressed throughout gametophyte development whereas *PpaWOX03 *transcripts were not detected (Figure 3B), thus supporting the hypothesis that *PpaWOX3 *could be a pseudogene.

An exhaustive data mining analysis of *A. thaliana *expression databases [30-33] indicated that, both *AtWOX13 *and *AtWOX14 *transcripts are present at different developmental stages *i. e.*, embryo development, plantlet stage, root formation, bolting stage, in flowers and in response to stress (see discussion). However, no *AtWOX10 *transcripts were present in any of the databases analyzed. In addition, RT-PCR analyses of seedlings, even with a high PCR cycle number, did not allow us to detect the *AtWOX10 *mRNA (Figure 3C). These results suggested that the recently duplicated *AtWOX10 *gene, found only in the *A. thaliana *genome, might be a pseudogene. Based on these observations, this gene was not further characterized. We conclude that in the three plant model genomes studied at least one copy of the WOX13OG genes is expressed during development.

### *AtWOX13 *shows a dynamic expression profile during *A. thaliana *development

**Figure 4:**
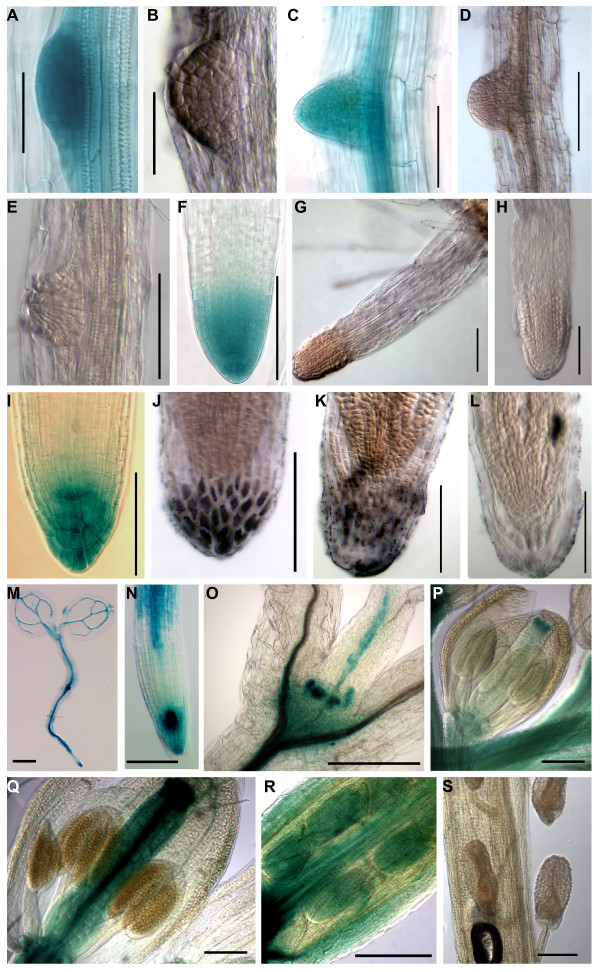
***AtWOX13 *gene expression during plant development**. **(A to L) **Expression profile during the initiation and development of lateral roots obtained with 10-day-old *AtWOX13::GUS *seedlings. Independent lines were tested. (A, C, F and I) GUS staining. (B, D, and K) *In situ *hybridization with an *AtWOX13 *antisense probe. (J) *In situ *hybridization with a GUS antisense probe used as a positive control. (E, H and L) *In situ *hybridization with a CUC2 antisense probe used as a negative control. **(M to S) **Expression profile at different plant developmental stages with independent *AtWOX13::GUS *lines. (M) 5-day-old seedling with staining in the leaf, the hypocotyl, the root vasculature, the pericycle and the primary root tip. (N) Magnification of a 5-day-old seedling primary root tip showing high staining in the stem cell of the proximal meristem. (O) Expression within the shoot apex of 10-day-old seedlings. Note the staining in the leaf primordia. **(P to S) **Expression within the flower at different developmental stages where staining is seen in the stigmata and the vasculature (P) in the gynoecium (Q) at the floral stage 12–13 where the ovules (Q) are stained. (R) Young embryo, (S) Mature silique. Scale bars = 100 μm except for A, B, R and S (= 50 μm) and M (= 500 μm).

Compared to other WOX proteins, the function of the AtWOX13 and 14 proteins have received little attention to date (see review [22]). In order to carry out a more detailed analysis of the WOX13 OG genes in *A. thaliana*, we cloned the gene coding for the *GUS *reporter under the control of the *AtWOX13 *and *AtWOX14 *upstream regulatory sequences. Based on our data mining analyses (see above), we first analyzed the GUS expression during root development. *AtWOX13 *promoter activity was detected during lateral root formation (Figure 4A, C, F and 4I). To validate the specificity of these data, *in situ *hybridization experiments were performed using respectively *AtWOX13 *(Figure 4B, D, G and 4K), *GUS *(Figure 4J) and *CUC2 *as probes (Figure 4E and 4H). The signal obtained using the *AtWOX13 *and *GUS *probes matched perfectly with the above GUS staining, whereas the *CUC2 *probe, as expected, did not give any specific signal. Hence, the GUS staining obtained reflects stable tissue specific mRNA expression and revealed that the *AtWOX13 *promoter sequence, used to drive the reporter gene *GUS *expression was sufficient to mimic the tissue specific localization of the endogenous *AtWOX13 *mRNA.

*AtWOX13 *expression was detected at the early stage of the lateral root (LR) formation of 10-days-old seedlings and it persisted during LR emergence (Figure 4A–D). The GUS staining was progressively restrained to the distal meristem (Figure 4F, and 4G) and it strongly accumulated in both the columella and the adjacent lateral root cap cells at a later developmental stage (Figure 4I–K). In mature LR or primary roots of 10-day-old seedlings no staining was detected within the root tip.

The *AtWOX13 *expression pattern was also investigated using the *AtWOX13::GUS *lines, during plant development. In 5-day-old seedlings, the promoter activity was strong in both cotyledon and root vasculatures, the pericycle and in the stem cells of the primary root meristem (Figure 4M and 4N). No GUS staining was detected in the division zone of the root. In the vegetative apex of 10 day-old seedlings, a strong staining was observed in the leaf primordia (Figure 4O). In the flower, the *AtWOX13 *promoter was active in the vasculature, the stigma (Figure 4P) and in the gynoecium at the flower stage 13/14 (Figure 4Q, and 4R). At this developmental stage, when fertilization was ongoing and pollen grains were germinating at the tip of the gynoecium, ovules stained blue (Figure 4Q). Later on, the young embryos remained stained (Figure 4R), but no staining was observed in mature siliques indicating that the *AtWOX13 *promoter was no longer active (Figure 4S), as observed before in all the other mature organs. During germination, soon after seed imbibition, GUS staining was also seen within the vasculature and the root tip, as observed in young seedlings (data not shown). Hence, *AtWOX13 *expression was restricted to differentiating organs and therefore probably regulated by developmental signals.

### *AtWOX14 *promoter specificity differs from that of *AtWOX13*

**Figure 5:**
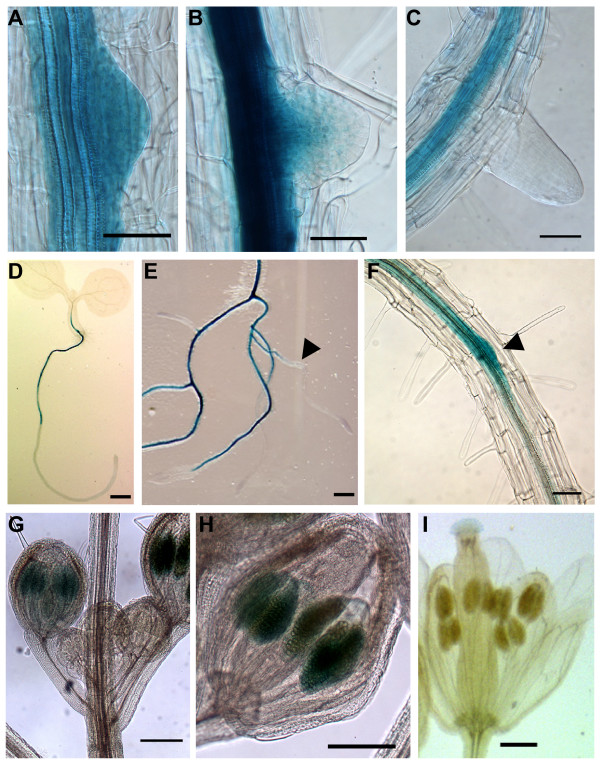
**AtWOX14 gene expression during plant development**. **(A to C) **Expression profile during the initiation and development of lateral roots obtained with 14-day-old *AtWOX14::GUS *seedlings. Independent lines were tested. **(D) **5-day-old seedling showing staining in the lower part of the hypocotyl and the upper part of the primary root vasculature. **(E) **Staining profile in primary and lateral roots of 14-day-old seedlings with expression in the upper root vasculature and pericycle. **(F) **Magnification at the edge of the GUS coloration showing the presence of the youngest LR initial (black arrowhead). **(G to I) **Expression within the flower showing strong staining in the anther only at floral stage 11–12 (G and H) before opening. Note the absence of staining in the mature anther (I). Scale bars = 100 μm except for C (= 200 μm) and D, E (= 500 μm).

Compared to the *AtWOX13 *expression patterns, the activity of *AtWOX14 *was more restrictive during lateral root formation. As for *AtWOX13*, the *AtWOX14 *promoter activity, revealed by GUS staining, was detected in the LR primordia, but decreased progressively as the LR emerged becoming restricted *in fine *to the vasculature up to the LR junction (Figure 5A–C). The *AtWOX13::GUS *gene continued to be expressed for a longer time (compare Figure 5B with Figure 4C). In 5-day-old seedlings, *AtWOX14 *driven GUS activity was only found in the vascular system and in the pericycle of the primary root (Figure 5D). In the LR of 14-day-old seedlings (Figure 5E), GUS staining was again observed in the vasculature and the pericycle with *AtWOX14 *expression being progressively induced in the pericycle and in the vasculature as the LR developed. Compared to the activity of the *AtWOX13 *promoter, *AtWOX14::GUS *expression stopped at a longer distance further up from the root tip (Figure 5E, black arrow), exactly at the position where the youngest LR primordia were initiated (see arrow in Figure 5F).

During flower development, *AtWOX14::GUS *expression was detected in the stamen at floral stage 11/12 (Figure 5G–I), when mitotic divisions of microspores are known to occur [34]. The staining decreased as the stamen matured, showing that the function of WOX14 is linked to the early stages of organ and tissue development. Hence, as for *AtWOX13*, *AtWOX14 *promoter activity also seems to be regulated by developmental signals. The gene expression profile reported in the gene atlas [35] and the tissues and organs affected by the mutation of *AtWOX14 *(see below) agree with the expression profile described above.

### An *AtWOX13 *gene truncated downstream of the homeodomain neither alters root nor flower development

**Figure 6:**
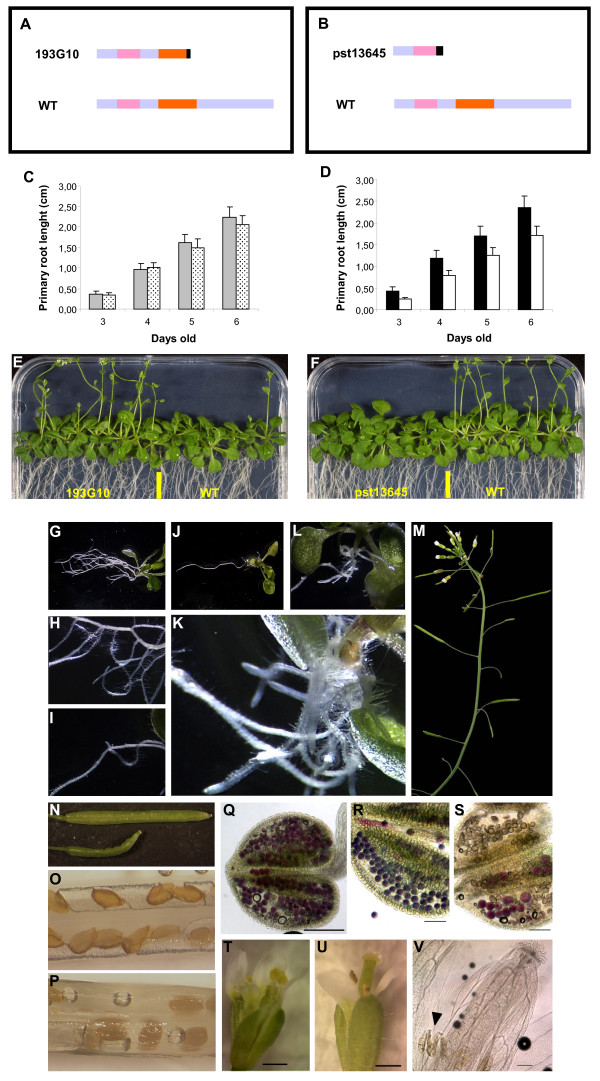
**The effects of the WOX13 OG mutations on *Arabidopsis thaliana *development**. **(A) **Diagram of the AtWOX13 protein of the *wox13 *line 193G10 and the wild type plants (WT). **(B) **Diagram of the AtWOX14 protein of the *wox14 *line pst13645 and the wild type plants (WT). *Legends: *WOX13MOG (pink), HD (orange)**(C and D) **Comparative primary root growth of mutant and wild type seedlings during the first 6 days of development. In (C), wild type WS ecotype (grey bar) and *wox13 *line 193G10 (dot bar). In (D), wild type No ecotype (black bar) and *wox14 *line pst13645 (white bar). **(E) **Floral transition of *wox13 *and WS plants after a long day period of 4 weeks.**(F) **Floral transition of *wox14 *and Nossen plants after a long day period of 4 weeks. **(G to K) **Root development of the wild type plant (G and H) compared to the *wox14 *line pst13645 (I to K) that overproduces adventitious roots. **(M to P) **Flower development of *wox14 *line pst13645 inflorescences that show partial sterility (M). Wild type (upper silique in N and O) and *wox14 *semi-sterile silique (lower siliques in N and P). (N and P) Pictures obtained after clearing. (**Q to S**) Pollen viability tests with Alexander staining. (Q) Wild type, (R) *wox14 *pollen. (S) *dmc1*/RNAi sterile mutant. (T to V). Mature flower development in wild type plants (T) and the *wox14 *line (U and V). (V) Picture after clearing. Scale bars: Q, V (= 100 μm), R, S (= 50 μm) and T, U (= 500 μm).

The only available *wox13 *mutant line, identified in GABI-Kat [36] and numbered 193G10, showed a T-DNA insertion upstream of the sequence coding for the characteristic WFQN motif located at the end of the WOX homeodomain (Figure 6A and see additional file 4: Figure 2A). RT-PCR experiments carried out using polyA^+^-RNA, prepared from the *wox13 *193G10 line, and primers matching each side of the insertion site, did not yield any PCR product (see Figure 3C). Because of the position of the insertion at the end of the WOX homeodomain, we also carried out an expression analysis using another couple of primers matching the 5'UTR of the *AtWOX13 *mRNA and the left border of the T-DNA. The latter experiment led to the amplification of a 500 bp PCR product, the sequence of which coded for a putative chimeric protein of 148 residues composed of the 143 amino acids of the AtWOX13 N-terminal region plus an additional 5 amino acids specified by the T-DNA sequence.

Based on the expression profiles obtained using the GUS reporter gene constructs, an analysis of the primary root growth of the mutant lines was carried out after seed germination. Surprisingly, the above *WOX13 *mutation neither affected primary root growth (Figure 6C), nor vegetative and flower development. The only phenotype appeared to be a much earlier floral transition (Figure 6E).

### A *WOX14 *knock-out mutant modifies lateral root and stamen development of *A. thaliana*

Conversely to the *wox13 *mutant, a *wox14 *insertion mutant line (*i.e.*, pst13645) with the entire WOX homeodomain deleted (Figure 6B and see additional file 4: Figure 2B) displayed developmental defects in all the organs where GUS staining was observed. The primary root growth was retarded (Figure 6D) and after flowering, the pst13645 insertion line showed partial sterility (Figure 6M), with aborted and shorter siliques compared to the wild type (Figure 6N). The floral transition was also altered, however the phenotype observed was that of a late flowering mutant (Figure 6F).

The *wox14 *mutant was further characterized during plant development. Whereas in the wild type Nossen a fully developed network of LR was observed (Figure 6G, H), the LR formation in *wox14 *was strongly inhibited (Figure 6I, J). The pst13645 line displayed extra adventitious roots (see Figure 6K, L) whereas the wild type plants developed only one or two. At the reproductive stage, observations after tissue clearing showed that the shorter siliques of *wox14 *contained many ovules that did not develop into seeds (Figure 6P), compared to the wild type (Figure 6O). Since the *AtWOX14 *promoter was only active in the developing stamen, pollen grain viability was checked using Alexander staining. As shown in Figure 6Q and 6R, the anthers of both the wild type and the *wox14 *mutant contained viable pollen grains when compared to the sterile *dmc1 *RNAi line [37]. However, stamen development was severely affected in the *wox14 *mutant. In open flowers of the wild type plants, the stamens were longer than the gynoecium allowing complete fertilization of the ovule (Figure 6T). In contrast, in the mutant line, the stamen did not fully develop and remained shorter than the gynoecium thereby preventing efficient fertilization and causing ovule abortion. In some *wox14 *flowers, the phenotype was even more severe, with aborted stamens that developed to only half the size of the gynoecium (Figure 6V). These data suggested that *AtWOX14 *prevents premature organ and tissue differentiation at the floral stage.

## Discussion

To improve previously described and not completely congruent WOX phylogenies [14,16,38] we performed multi alignments on protein sequences (60–61 amino acids) longer than the usually defined HD (41–42 amino acids). Furthermore, we used gene or protein features other than the HD sequence to support the three OGs exhibited by our phylogenic trees. Even if the WOX gene phylogeny remains difficult to reconcile with species phylogeny, we are confident about the gene distribution between the three main OGs. Only one *WOX *gene was detected in the basal unicellular and photosynthetic organism *O. tauri *and three WOX genes were identified in moss *P. patens*, including at least two functional paralogues. The WOX13 OG is the clade containing the most conserved WOX genes as it is the only clade that contains the *O. tauri*, the *P. patens *and probably the eight *S. moellendorffii *genes. It also showed a recent and specific expansion in Brassicaceae. These observations point out the interest in the study of the WOX13 OG with respect to its biological specificity and gene family evolution.

### WOX14 orthologues are only observed in Brassicaceae species

We detected *AtWOX13 *and *AtWOX14 *mRNAs at different stages of plant development, in plantlets, roots, flowers and developing embryos. These expression profiles were in accordance with those described in a compendium of *A. thaliana *expression databases for the WOX13 OG genes [30-33]. For instance, in the gene expression map of the Arabidopsis root [39], the AtWOX13 mRNA profile shows a localized expression domain (LED) specific to the stele in the elongation zone of the mature primary root as observed in our study. These databases also revealed that the expression levels of both *AtWOX14 *and *AtWOX13 *were low compared to other WOX genes, suggesting either a tight control of their expression or a cell specific expression pattern. This has been confirmed by the expression data described in this paper.

The *AtWOX14 *branch within the WOX13 OG, has previously been suggested to be unique to *A. thaliana *[14]. Our study based on more species indicates that this branch also contains sequences from other Brassicaceae. Interestingly, differences in the expression profiles between *AtWOX14 *and *AtWOX13 *indicate that their 5' regulatory elements have diverged since gene duplication. In line with this conclusion, our data mining analyses showed that *AtWOX13 *expression, but not that of *AtWOX14*, was modulated in response to biotic and abiotic stresses. Hence, the *AtWOX14 *promoter appears to have diverged to presumably support specific developmental mechanisms found so far only in Brassicaceae. Analysis of the expression profile of a non Brassicaceae *WOX13 *gene should give us a better understanding of the evolutionary mechanisms giving rise to this particular branch.

### WOX13 OG genes affect organogenesis and floral transition

The control of cell proliferation and differentiation during organ development are essential processes that require genes implicated in cell signaling, cell identity and cell cycle transitions. In higher plants, both the SAM and the root pericycle cells serve as initiation sites of organogenesis [40,41]. In this paper, we show that *AtWOX13 *and *AtWOX14 *are active in organ primordia and during early tissue differentiation. In the transcriptomic database CATdb [30], in which only the AtWOX13 probe is available, 5 out of 62 experiments associate *AtWOX13 *gene expression with cell proliferation and growth. Interestingly, the transcriptome profiling experiments, using protoplast cultures [42], showed that the *AtWOX13 *mRNA level followed an expression profile similar to that of the *proliferating cell nuclear antigen 1 *gene (see additional file 4: Figure 3A), a key regulator of DNA metabolism and cell cycle progression [43]. Conversely, the homeobox gene *ATHB7*, a marker of cell differentiation and elongation [44], strongly decreased in the same protoplast culture. Compared to the cell cycle gene (see additional file 4: Figure 3B), *AtWOX13 *expression levels were maximal when the cells entered cell division. *AtWOX13 *expression was also up-regulated in the *tor *mutant or after a treatment with the IP3K inhibitor, wortmannin. The TOR protein and IP3K are regulators of cell division and growth. TOR has only been found in non-differentiated and rapidly dividing meristematic cells [45,46]. However, the dynamic expression of *AtWOX13 *during LR development described in this paper does not entirely overlap with the cell division zone, as *AtWOX13 *promoter activity became more restricted to the vasculature of the root.

Our data also showed that mutations of the *AtWOX13 *and *14 *genes affected the floral transition. An increase of cell division in all the vegetative SAM was proposed to be a prerequisite for morphological changes at the floral transition in plants [47,48]. Furthermore, in *A. thaliana *stem axis growth is the result of cell elongation that occurs only in the rib zone and not in the upper part of the SAM [49]. Hence, the late flowering phenotype observed in the *wox14 *mutant might reflect a default in the coordination of cell division and elongation in the transitional SAM. In contrast, the *wox13 *mutant line showed a surprising early floral transition. However, a chimeric *WOX13 *mRNA coding for a putative protein containing the conserved domains was expressed in the *wox13 *193G10 line. We suggest that this line might act as a dominant negative mutant that induces early floral transition by activating precocious cell proliferation in the SAM. Alternatively, we cannot exclude that other WOX family members could fulfill the function of the AtWOX13 protein, leading to a weak phenotype in the Arabidopsis mutant background. To date, the mechanism underlying gene regulation by the homeobox gene family is complex and remains largely unclear [50]. A monitoring of the promoter activity during the floral transition and an analysis of a mutant line with an insertion upstream of the HD or an RNAi strategy should give us better insights into the function of the WOX13 protein.

Finally, we found that the *wox14 *null mutant showed root growth delay and early anther maturation. Interestingly, a similar phenotype was also observed in anthers when cyclin-dependent kinase inhibitors were ectopically expressed in plants [51-53]. Hence, it is tempting to speculate that the AtWOX14 protein is involved in the negative control of cell differentiation to allow for correct development.

In a similar way, the expression profile observed during the gametophytic development of *P. patens *is consistent with a putative function in cell differentiation control during the filamentous growth and the bud formation although *in situ *hybridization experiment are needed to validate this hypothesis. Last, the constitutive expression of the only *WOX *gene in *O. tauri *may account for its maintenance in an undifferentiated state. Future use of *O. tauri *and *P. patens *as biological models and ongoing complementation experiments of mutants using either full length or truncated proteins should allow us to obtain a better understanding of the functional importance of the WOX13 OG genes.

## Conclusion

Our data provide evidence in favor of the WOX13 OG as the clade containing the most conserved WOX genes. The preferential presence of amino acid motifs fully supports the separation of the different gene groups in the phylogenetic trees. However, the latter is not consistent with the green plant phylogeny [21]. In this study, we linked the function of the WOX13OG Arabidopsis members to organ initiation and development, most likely by preventing premature differentiation as shown for other WOX proteins. The data also suggest that the WOX family has diverged both at the transcriptional and protein level to generate new features that control cell identity in specific domains of land plants. In line with such evolutionary events, the expression profile and mutant phenotype of the *AtWOX14 *gene suggest that the Brassicaceae might have developed specific mechanisms to control floral transition and pollen formation when compared to other plants.

## Methods

### Identification of WOX genes

The identification of *WOX *genes was undertaken using model plants for which the complete genome was available, *i.e.*, *A. thaliana *(a core eudicot), *O. sativa *(a monocot), *P. patens *(a moss) and *O. tauri *(a green alga). The WOX family was defined in *A. thaliana *and *O. sativa *genomes after a BLASTp [54] search in the FLAGdb++ database [55] using AtWUS (AT2G17950). *AtWUS-like *sequences were identified in the *P. patens *whole genome shotgun database ftp://ftp.ncbi.nih.gov/pub/TraceDB/physcomitrella_patens/, the *O. tauri *[56] and the *O. lucimarinus *[57] genomes after a BLASTp using the HD amino acid sequence. The limits of the sub-families were defined following the drop-in-Blast score method [58,59]. The structures of the identified genes were verified using all of the corresponding transcripts and when necessary the gene was reannotated. There was only one *WOX *gene, (OT13G01350), within the *O. tauri *and the *O. lucimarinus *genomes. Gene and protein sequences of the WOX families from completely sequenced genomes of model species are given (see additional file 3: Table 1). The positions of introns were obtained from nucleotide sequence alignments derived from the protein alignments. Only introns inserted into genomic regions coding for sufficiently similar protein sequences were used to study the conservation of intron insertion sites.

The proteome of *S. moellendorffii *was downloaded from the JGI ftp site ftp://ftp.jgi-psf.org/pub/JGI_data/Selaginella_moellendorffii/. Using the WOX-HD HMM (see additional file 3: Table 2), we found 8 WOX sequences, but only 3 were apparently full-length. Only the complete SmWOX sequences were used for the phylogeny reconstruction of the WOX family.

### Distance trees and phylogeny reconstruction of the WOX family

Neighbor-Joining (NJ) [60], Maximum likelihood (ML) [61] trees, both based on Jones-Taylor-Thornton matrix [62], and Parsimony [63] trees were constructed using PHYLIP [64]. To assess support for the calculated relationships, 1000 bootstrap samples were generated [65]. To select appropriate outgroups for the WOX model plant phylogeny, we first established a tree using all of the HD proteins from *A. thaliana *and 3 outgroups (*i.e.*, At3G03260, At4G00730, At4G17710) were chosen from the three clades closest to the WOX clade. Trees were computed from 60 to 61 amino acid sequences containing the HD of the WOX proteins from the five plant model genomes, *O. tauri*, *P. patens, S. moellendorffii *(only the 3 available full-length proteins were used), *A. thaliana *and *O. sativa*. The clustering of the WOX proteins in three different OGs obtained from the genome models were consistent using both NJ (Figure 1) and ML (see additional file 1: Figure 1) methods and the 3 above outgroups either independently or together (data not shown). We also built up phylogenetic tree from the same set of data using the Parsimony method. Different branching patterns were obtained depending upon which outgroup was used. With the multiple outgroup described above, *O. tauri *and *S. moellendorffii *were both independent branches at the base of the tree (see additional file 1: Figure 2).

The WOX13 OG trees has been established using the Neighbor-Joining [60] method based on Henikoffs evolutionary distances [66]. The trees obtained with the ML (see additional file 1: Figure 3) and Parsimony methods (see additional file 1: Figure 4) also gave the same main clusters of proteins.

### Analysis of WOX protein sequences

In order to identify shared motifs among the protein sequences we used the MEME program, version 3.5.4 [67]. A gapped alignment using CLUSTALW was generated for each motif. Based on these multiple alignments a Hidden Markov Model (HMM) was built using HMMER 2.3.2 [20]. The HMM was used to search for motifs in the WOX sequences from the model genomes and in the whole proteome in GenBank NR (see additional file 3: Table 2). The sequence logos were made using Weblogo [68]. The specificity of the HMMs for the different WOX clades was tested using the two recently available Vitis vinifera genome sequences, PN40024 [69] and Pinot noir [70]. Using the WOX HD HMMs, an initial search in these two novel proteomes allowed us to specifically retrieve all members of the WOX gene family. A second search with the clade specific HMMs efficiently clustered the WOX genes into one of the three WOX clades (see additional file 3: Table 3). We found 13 different Vitis genes coding for WOX proteins, with 8 located in the WOX1 OG, 3 in the WOX13 OG, and 2 in the WOX8 OG. No V. vinifera proteins were located to the WOX14 clade.

### Plant material

A naturally synchronized *O. tauri *strain OTTH0595 cell culture was used and the cell cycle analysed by flow cytometry as described by Farinas et al [27]. Using a 12 h light/dark condition, we obtained two subpopulations of synchronized cells at the light/dark transition, comprised of 12% cells in the G2/M phase and the remaining cells in the S phase of the cell-cycle. The moss *P. patens *was grown on BCD medium [71] overlaid with cellophane under 16 h light/8 h dark cycle at 65% hygrometry and at 20°C. The DS transposon tagged *A. thaliana *line *wox14*, *i.e.*, pst13645, was obtained from the RIKEN BRC Japan. The T-DNA tagged *A. thaliana *line *wox13, i.e.*, 193G10, was purchased from the INRA Versailles resource centre. The *wox13 *and *wox14 *mutant lines are in the Wassilewskija (Ws) and the Nossen (No) background, respectively. Wild type plants of both ecotypes were used as background controls.

### Root growth assays

Sterile seeds were placed on a solid growth medium (MS salt supplemented with 20 g/L sucrose and agar) at 4°C for 2 days, and grown under 16 h light/8 h dark cycles (20°C during illumination, 18°C in the dark) at 65% hygrometry. Root length was measured from day 3 to day 6 using the ImageJ free software (NIH, USA) after scanning.

### Characterization of the mutant lines

The pst13645 and 193G10 lines were screened using media containing either 30 mg/L hygromycin or 30 mg/L kanamycin, respectively. In order to verify the mapping of the insertion site, F2 or F3 plants were genotyped at the *AtWOX13*, At *WOX14 *loci. Specific genomic DNA primers and either T-DNA or the Ds-element border-specific primers were designed based on the annotated insertion site of each mutant line (see additional file 4: Table 1). The resulting PCR products were separated on a 2% agarose gel and their size was verified. 341 bp and 163 bp fragments were expected for the *AtWOX13 *wild type and mutated allele, respectively and 498 bp and 216 bp fragments for the *AtWOX14 *wild type and mutated allele, respectively. Moreover, the entire *AtWOX14-AtWOX10 *DNA region was genotyped to check for the absence of chromosome rearrangements of the duplicates and to confer that the phenotype was only due to the disruption of *AtWOX14*. Phenotypes were observed only in homozygous plants for each mutation and therefore they were used in all further experiments.

### Reverse transcriptase PCR

Total RNA was extracted from the different plants using the Qiagen plant RNeasy extraction kit and DNAseI treated following the manufacturer's instructions. The RNA preparations were reverse transcribed using 1 to 5 μg of RNA. RT-PCR experiments were performed in order to verify the expression of the different *WOX *and control genes (see additional file 4: Table 1) within the different plant model species and the transgenic Arabidopsis plants. Primer efficiency was tested using genomic DNA and eventual genomic contamination was always checked by PCR amplification of the RNA samples using the equivalent amount of cDNA used for the RT-PCR. Quantitative real-time RT-PCR was also performed on *O. tauri *mRNA as described previously [72] using a Roche Light Cycler ^® ^480 with the SYBER^© ^green PCR mix from either Roche or Applied Biosystem. For each gene tested, PCR products from genomic DNA cloned into the PGEMT^© ^vector and the *Not*I digested empty plasmid were used as standards to quantify the corresponding RNA copy number. These raw copy numbers were then normalized to the amount of equivalent RNA used for the quantification.

### Construction of the WOX::GUS reporter genes

To clone the *AtWOX13 *and *AtWOX14 *promoters, the entire intergenic regions upstream of the ATG initiators were PCR-amplified using gene specific primers containing either an *Xba*I or a *BamH*I restriction site (5'-TCTCGAGTGGAGCTTTTGCAGGTCTCT-3'; 5'-AGGATCCTCAG AATTTCGCTCAGAAGATTT-3') for AtWOX13 and containing either a *NotI *or a *Spe*I restriction site (5'-GACAGCGGCCGCGGGGTTGTGAGTCCTATTGC-3'; 5'-GACACTAGTTGAACAAGACAATGAGAAAGTGAA-3') for *AtWOX14*. DNA fragments (of 3.6 kb and 1 kb for the *AtWOX13 *and *AtWOX14 *promoters, respectively) were subcloned into the pGEMT vector (Promega) to give the prom *WOX13 *and prom *WOX14 *plasmids. For the *WOX::GUS *reporter constructs, prom *WOX13 Xho*I/*BamH*I and prom *WOX14 Spe*I fragments were subcloned into a *Sal*I/*BamH*I and an *Xba*I digested pPR97 binary vector, respectively [73]. Constructs were introduced into the *Agrabacterium tumefaciens *strain GV3101, then into Arabidopsis plants ecotype Ws as described in [74]. Transformed plants were selected by adding 30 mg/L kanamycin to the growth medium. Resistant plants were transferred to soil and grown in a growth chamber.

### Staining and observations

Beta-glucuronidase (GUS) staining was performed for 24 h on T1 and T2 seedlings as previously described [75], except for the lateral roots where the incubation time was limited to 4 h. After fixing and clearing, samples were mounted on slides in HCG (80% Chloral hydrate, 10% Glycerol, 10% water) and observed under a Zeiss light microscope or a Zeiss binocular. Cytological observations of Alexander-stained anthers were carried out as previously described [76].

### In situ hybridization

*In situ *hybridizations were conducted as previously described [77] using antisense probes. The *AtWOX13 *antisense probe was synthesized in vitro using a PCR product obtained with the following primers: (CCTGCAGATGATGGAATGGGATAATCAGC and TGTAATACGACTCACTATAGGGCACTGCTTATGACTGACTACCAAATCC). The CUC2 probe has been described previously [77] and the GUS probe was provided by the laboratory of P. Laufs (INRA Versailles).

## Authors' contributions

The project was conducted by YD, MK and AL. The bioinformatics analyses were done by CTN, VT and AL; YD and GC carried out all the experimental works with the assistance of H. Morin (in conducting the in situ hybridization) and H. Moreau, the *O. tauri *experiments. YD, MK and AL wrote this manuscript; PL revised several versions of this manuscript. All authors read and commented on drafts of the manuscript and approved the final manuscript.

## Supplementary Material

Additional file 1 **Phylogenetic trees**. **Figure 1 : Maximum-likelihood tree of the WOX proteins from the 5 completely sequenced genomes of the green lineage: ***Arabidopsis thaliana *(AT prefix), *Oryza sativa *(OS prefix), *Physcomitrella patens *(Ppa prefix), *Ostreococcus tauri *(OT prefix) and the three full-length proteins of *Selaginella moellendorffii *(Sm prefix). The significance of each node was tested using 1000 bootstrap replicates. Only bootstrap values above 50% are shown. The scale, at the right of the tree, indicates 0.2 substitution per site. **Figure 2 : Parsimony tree of the WOX proteins from the 5 completely sequenced genomes of the green lineage: ***Arabidopsis thaliana *(AT prefix), *Oryza sativa *(OS prefix), *Physcomitrella patens *(Ppa prefix), *Ostreococcus tauri *(OT prefix) and the three full-length proteins of *Selaginella moellendorffii *(Sm prefix). The significance of each node was tested using 1000 bootstrap replicates. Only bootstrap values above 50% are shown. **Figure 3 : Maximum-likelihood tree of the WOX proteins from the WOX13 Orthology Group**. The significance of each node was tested using 1000 bootstrap replicates. Only bootstrap values above 50% are shown. The scale, at the right of the tree, indicates 0.1 substitution per site. Species names, in alphabetic order, are: Afo: *Aquilegia formosa*; AT: *Arabidopsis thaliana*; Bna: *Brassica napus*; Bra: *Brassica rapa*; Ccl: *Citrus clementina*; Csi: *Citrus sinensis*; Cte: *Citrus temple*; Ees: *Euphorbia esula*; Ghi: *Gossypium hirsutum*; Gma: *Glycine max*; Gra: *Gossypium raimondii*; Han: *Helianthus annuus*; Hvu: *Hordeum vulgare*; Les: *Lycopersicon esculentum*; Lja: *Lotus japonicus*; Lsa: *Lactuca sativa*; Lvi: *Lactuca virosa*; Mdo: *Malus domestica*; Mtr: *Medicago truncatula*; OS: *Oryza sativa*; OT: *Ostreococcus tauri*; Ppa: *Physcomitrella patens*; Psi: *Picea sitchensis*; Pta: *Pinus taeda*; Ptr: *Populus tremula *× *Populus tremuloides*; Pvu: *Phaseolus vulgaris*; Stu: *Solanum tuberosum*; Vvi: *Vitis vinifera*; Zma: *Zea mays*. **Figure 4 : Parsimony tree of the WOX proteins from the WOX13 Orthology Group**. The significance of each node was tested using 1000 bootstrap replicates. Only bootstrap values above 50% are shown. Species names, in alphabetic order, are: Afo: *Aquilegia formosa*; AT: *Arabidopsis thaliana*; Bna: *Brassica napus*; Bra: *Brassica rapa*; Ccl: *Citrus clementina*; Csi: *Citrus sinensis*; Cte: *Citrus temple*; Ees: *Euphorbia esula*; Ghi: *Gossypium hirsutum*; Gma: *Glycine max*; Gra: *Gossypium raimondii*; Han: *Helianthus annuus*; Hvu: *Hordeum vulgare*; Les: *Lycopersicon esculentum*; Lja: *Lotus japonicus*; Lsa: *Lactuca sativa*; Lvi: *Lactuca virosa*; Mdo: *Malus domestica*; Mtr: *Medicago truncatula*; OS: *Oryza sativa*; OT: *Ostreococcus tauri*; Ppa: *Physcomitrella patens*; Psi: *Picea sitchensis*; Pta: *Pinus taeda*; Ptr: *Populus tremula *× *Populus tremuloides*; Pvu: *Phaseolus vulgaris*; Stu: *Solanum tuberosum*; Vvi: *Vitis vinifera*; Zma: *Zea mays*. Click here for file

Additional file 2 **WOX gene structures and protein motifs**. **Figure 1 : The structure of WOX genes from model genomes**. The gene structures from 4 model genomes (*Arabidopsis thaliana*, AT; *Oryza sativa*, OS; *Physcomitrella patens*, Ppa; *Ostreococcus tauri*, OT) and three *Selaginella moellendorffii *(Sm) genes are displayed following the order of the Neighbour-joining tree in Figure 1 and aligned on their translation start. Rectangles stand for exons while lines denote introns. Blue is for exonic regions in UTRs and light grey is for translated exons. Five different gene structures are given for *PpaWOX02 *due to the 5' UTR variants supported by transcripts. **Figure 2 : WOX proteins: Sequence alignments around the virtual intron insertion sites**. For each WOX OG, 15 amino acid sequences both upstream and downstream introns are given. The intron insertion sites are indicated by stars. Numbers on the left of sequences give the intron length. Digits between stars stand for the phase of the intron insertion site. The intron phase is defined by the position of its insertion into a codon. An intron can be located between two codons (phase 0) or within a codon, lying either after the first or after the second base pair (phase 1 and phase 2 respectively). Boxes indicate conserved introns with the same color code than in Figure 1. **Figure 3 : Sequence logos of protein motifs in the WOX family**. Each logo http://weblogo.berkeley.edu consists on stack of letters, one stack for each position in the sequence. The overall height of the stack indicates the sequence conservation at that position, while the letter's height within the stack indicates the relative information content of each amino acid at that position. The amino acid color code is dark blue for large polar, light blue for basic, black for small polar, green for nonpolar, orange for cysteine and purple for ambivalent amino acids. **Figure 4 : WOX motifs: alignments for motifs without Logo**. Motifs defined by less than 6 sequences cannot be shown as logo since the sequence information at each position is too weak. Thus multiple alignments performed with Clustalw with a majority consensus are shown here for theses motifs. Amino acid color code is the same than in Figure 2. Click here for file

Additional file 3 **WOX sequences and HMM motifs**. **Table 1: Gene and protein sequences of the WOX family in the model genomes *A. thaliana*, *O. sativa*, *P. patens *and *O. tauri***. **Table 2: Specificity and sensitivity of HMM for WOX motifs**. Scores can give indications to distinguish potential family -or subfamily-members from false positive. A clear drop in the score could be detected in most cases, indicating that sequence below this threshold did not fulfill the family model as well as above [21]. HMMER bit scores were obtained for each motif by querying the HMM against three protein sequence sets: WOX13 OG (33 sequences), model genome WOX (32 sequences) and GenBank NR (Release 157). The score drops above 50 and indicating the motifs exhibiting the highest specificities with a given database are in bold. a: no hit was found in the set. b: no specific hit was found in the GenBank NR database. Note that Physcomitrella sequences from genome model WOX set are not present in GenBank NR. c: best hits are not belonging to the WOX family and no score drop was found. **Table 3: *Vitis vinifera *WOX protein prediction with HMM WOX motifs**. HMM search scores above a drop give indication to assign novel WOX proteins to the different WOX OGs. AM(...) IDs are contigs from Pinot noir *V. vinifera *while contig(...) IDs are from PN40024 *V. vinifera*. Dash indicates that no score was found above the drop. a: no hit was found with this HMM profile. (*): 17.4 score is due to an undefined C or T nucleotide at one position leading to a stop in the CDS. After fixing the nucleotide to C, the score is 27.7. Click here for file

Additional file 4 **Expression analyses**. **Figure 1 : Real-time PCR analysis of *Ostreoccocus tauri *gene expression in a 12-hour light/12-hour dark synchronized cell culture**. Copy number of *WOX, KNOX*, *Histone4 *and *CyclinB *RNA was quantified using total RNA sampling from the starting of the light period (0 h) or from continuous light grown cell culture. **Figure 2 : Alignments of predicted sequences of AtWOX13 (A) and AtWOX14 (B) chimeric proteins with the full length ones**. The homeodomain are indicated in bold and the amino acid changes at the end of the chimeric proteins indicated in lower case. **Figure 3 : *AtWOX13 *expression is correlated with the induction of cell proliferation**. Data mining analysis of the expression profiles of a set of genes in protoplast culture of *Arabidopsis thaliana*. **(A) ***AtWOX13 *(-◆-), *ATHB-7 *(■), *PCNA1 *(▲); **(B) **Expression profile of a cluster of cell cycle genes including *AT4G37490 (CYC1)*; *AT1G44110 (CYCA1;1 *(■)); *AT5G11300 (CYC3B)*; *AT1G47210 (CYCA3;2)*; *AT3G05330 (CDK)*; *AT2G26760 (CYCB1;4)*; *AT4G35620 (CYCB2;2)*; *AT2G26430 (RCY1)*; *AT2G31400 (ATPDNA Binding)*. **Table 1: **Primer sequences designed for expression analysis, genotyping (g primers) and cDNA cloning (2 last row) using primer3 software. Click here for file

## References

[B1] Haecker A Gross-Hardt R Geiges B Sarkar A Breuninger H Herrmann M Laux T Expression dynamics of WOX genes mark cell fate decisions during early embryonic patterning in Arabidopsis thaliana Development 2004 131 3 657 668 10.1242/dev.00963 14711878

[B2] Nardmann J Werr W The Shoot Stem Cell Niche in Angiosperms: Expression Patterns of WUS Orthologues in Rice and Maize Imply Major Modifications in the Course of Mono- and Dicot Evolution Mol Biol Evol 2006 10.1093/molbev/msl125 16987950

[B3] Gallois JL Nora FR Mizukami Y Sablowski R WUSCHEL induces shoot stem cell activity and developmental plasticity in the root meristem Genes Dev 2004 18 4 375 380 10.1101/gad.291204 15004006 PMC359391

[B4] Park SO Zheng Z Oppenheimer DG Hauser BA The PRETTY FEW SEEDS2 gene encodes an Arabidopsis homeodomain protein that regulates ovule development Development 2005 132 4 841 849 10.1242/dev.01654 15659481

[B5] Kamiya N Nagasaki H Morikami A Sato Y Matsuoka M Isolation and characterization of a rice WUSCHEL-type homeobox gene that is specifically expressed in the central cells of a quiescent center in the root apical meristem Plant J 2003 35 4 429 441 10.1046/j.1365-313X.2003.01816.x 12904206

[B6] Wurschum T Gross-Hardt R Laux T APETALA2 regulates the stem cell niche in the Arabidopsis shoot meristem Plant Cell 2006 18 2 295 307 10.1105/tpc.105.038398 16387832 PMC1356540

[B7] Zuo J Niu QW Frugis G Chua NH The WUSCHEL gene promotes vegetative-to-embryonic transition in Arabidopsis Plant J 2002 30 3 349 359 10.1046/j.1365-313X.2002.01289.x 12000682

[B8] Mayer KF Schoof H Haecker A Lenhard M Jurgens G Laux T Role of WUSCHEL in regulating stem cell fate in the Arabidopsis shoot meristem Cell 1998 95 6 805 815 10.1016/S0092-8674(00)81703-1 9865698

[B9] Sarkar AK Luijten M Miyashima S Lenhard M Hashimoto T Nakajima K Scheres B Heidstra R Laux T Conserved factors regulate signalling in Arabidopsis thaliana shoot and root stem cell organizers Nature 2007 446 7137 811 814 10.1038/nature05703 17429400

[B10] Wu X Dabi T Weigel D Requirement of homeobox gene STIMPY/WOX9 for Arabidopsis meristem growth and maintenance Curr Biol 2005 15 5 436 440 10.1016/j.cub.2004.12.079 15753038

[B11] Riou-Khamlichi C Menges M Healy JM Murray JA Sugar control of the plant cell cycle: differential regulation of Arabidopsis D-type cyclin gene expression Mol Cell Biol 2000 20 13 4513 4521 10.1128/MCB.20.13.4513-4521.2000 10848578 PMC85832

[B12] Planchais S Samland AK Murray JA Differential stability of Arabidopsis D-type cyclins: CYCD3;1 is a highly unstable protein degraded by a proteasome-dependent mechanism Plant J 2004 38 4 616 625 10.1111/j.0960-7412.2004.02071.x 15125768

[B13] Nardmann J Ji J Werr W Scanlon MJ The maize duplicate genes narrow sheath1 and narrow sheath2 encode a conserved homeobox gene function in a lateral domain of shoot apical meristems Development 2004 131 12 2827 2839 10.1242/dev.01164 15169755

[B14] Nardmann J Zimmermann R Durantini D Kranz E Werr W WOX Gene Phylogeny in Poaceae: A Comparative Approach Addressing Leaf and Embryo Development Mol Biol Evol 2007 24 11 2474 2484 10.1093/molbev/msm182 17768306

[B15] Richardt S Lang D Reski R Frank W Rensing SA PlanTAPDB, a Phylogeny-Based Resource of Plant Transcription-Associated Proteins Plant Physiol 2007 143 4 1452 1466 10.1104/pp.107.095760 17337525 PMC1851845

[B16] Nardmann J Werr W The evolution of plant regulatory networks: what Arabidopsis cannot say for itself Curr Opin Plant Biol 2007 10 6 653 659 10.1016/j.pbi.2007.07.009 17720614

[B17] Philippe H Laurent J How good are deep phylogenetic trees? Curr Opin Genet Dev 1998 8 6 616 623 10.1016/S0959-437X(98)80028-2 9914208

[B18] Theissen G Kim JT Saedler H Classification and phylogeny of the MADS-box multigene family suggest defined roles of MADS-box gene subfamilies in the morphological evolution of eukaryotes J Mol Evol 1996 43 5 484 516 10.1007/BF02337521 8875863

[B19] Ooka H Satoh K Doi K Nagata T Otomo Y Murakami K Matsubara K Osato N Kawai J Carninci P Comprehensive analysis of NAC family genes in Oryza sativa and Arabidopsis thaliana DNA Res 2003 10 6 239 247 10.1093/dnares/10.6.239 15029955

[B20] Eddy SR Profile hidden Markov models Bioinformatics 1998 14 9 755 763 10.1093/bioinformatics/14.9.755 9918945

[B21] Bowman JL Floyd SK Sakakibara K Green genes-comparative genomics of the green branch of life Cell 2007 129 2 229 234 10.1016/j.cell.2007.04.004 17448980

[B22] Chandler J Nardmann J Werr W Plant development revolves around axes Trends Plant Sci 2008 13 2 78 84 18262821 10.1016/j.tplants.2007.11.010

[B23] Kieffer M Stern Y Cook H Clerici E Maulbetsch C Laux T Davies B Analysis of the transcription factor WUSCHEL and its functional homologue in Antirrhinum reveals a potential mechanism for their roles in meristem maintenance Plant Cell 2006 18 3 560 573 10.1105/tpc.105.039107 16461579 PMC1383633

[B24] Hiratsu K Mitsuda N Matsui K Ohme-Takagi M Identification of the minimal repression domain of SUPERMAN shows that the DLELRL hexapeptide is both necessary and sufficient for repression of transcription in Arabidopsis Biochem Biophys Res Commun 2004 321 1 172 178 10.1016/j.bbrc.2004.06.115 15358231

[B25] Tiwari SB Hagen G Guilfoyle TJ Aux/IAA proteins contain a potent transcriptional repression domain Plant Cell 2004 16 2 533 543 10.1105/tpc.017384 14742873 PMC341922

[B26] Scofield S Murray JA KNOX Gene Function in Plant Stem Cell Niches Plant Mol Biol 2006 60 6 929 946 10.1007/s11103-005-4478-y 16724262

[B27] Farinas B Mary C O Manes C-Ld Bhaud Y Peaucellier G Moreau H Natural Synchronisation for the Study of Cell Division in the Green Unicellular Alga Ostreococcus tauri Plant Molecular Biology 2006 V60 2 277 292 10.1007/s11103-005-4066-1 16429264

[B28] Mingam A Toffano-Nioche C Brunaud V Boudet N Kreis M Lecharny A DEAD-box RNA helicases in Arabidopsis thaliana: establishing a link between quantitative expression, gene structure and evolution of a family of genes Plant Biotechnology Journal 2004 2 5 401 415 10.1111/j.1467-7652.2004.00084.x 17168887

[B29] Schumaker KS Dietrich MA Programmed Changes in Form during Moss Development Plant Cell 1997 9 7 1099 1107 10.1105/tpc.9.7.1099 12237377 PMC156983

[B30] Gagnot S Tamby J-P Martin-Magniette M-L Bitton F Taconnat L Balzergue S Aubourg S Renou J-P Lecharny A Brunaud V CATdb: a public access to Arabidopsis transcriptome data from the URGV-CATMA platform Nucl Acids Res 2007 gkm757 10.1093/nar/gkm757 PMC2238931 17940091

[B31] Meyers BC Tej SS Vu TH Haudenschild CD Agrawal V Edberg SB Ghazal H Decola S The Use of MPSS for Whole-Genome Transcriptional Analysis in Arabidopsis Genome Res 2004 14 8 1641 1653 10.1101/gr.2275604 15289482 PMC509274

[B32] Zimmermann P Hirsch-Hoffmann M Hennig L Gruissem W GENEVESTIGATOR. Arabidopsis Microarray Database and Analysis Toolbox Plant Physiol 2004 136 1 2621 2632 10.1104/pp.104.046367 15375207 PMC523327

[B33] Craigon DJ James N Okyere J Higgins J Jotham J May S NASCArrays: a repository for microarray data generated by NASC's transcriptomics service Nucl Acids Res 2004 32 suppl_1 D575 577 10.1093/nar/gkh133 14681484 PMC308867

[B34] Caryl AP Jones GH Franklin FC Dissecting plant meiosis using Arabidopsis thaliana mutants J Exp Bot 2003 54 380 25 38 10.1093/jxb/erg041 12456752

[B35] Schmid M Davison TS Henz SR Pape UJ Demar M Vingron M Scholkopf B Weigel D Lohmann JU A gene expression map of Arabidopsis thaliana development Nat Genet 2005 37 5 501 506 10.1038/ng1543 15806101

[B36] Li Y Rosso MG Viehoever P Weisshaar B GABI-Kat SimpleSearch: an Arabidopsis thaliana T-DNA mutant database with detailed information for confirmed insertions Nucleic Acids Res 2007 35 Database D874 878 10.1093/nar/gkl753 17062622 PMC1781121

[B37] Siaud N Dray E Gy I Gerard E Takvorian N Doutriaux MP Brca2 is involved in meiosis in Arabidopsis thaliana as suggested by its interaction with Dmc1 Embo J 2004 23 6 1392 1401 10.1038/sj.emboj.7600146 15014444 PMC381417

[B38] Richardt S Lang D Reski R Frank W Rensing SA PlanTAPDB, a phylogeny-based resource of plant transcription-associated proteins Plant Physiol 2007 143 4 1452 1466 10.1104/pp.107.095760 17337525 PMC1851845

[B39] Birnbaum K Shasha DE Wang JY Jung JW Lambert GM Galbraith DW Benfey PN A Gene Expression Map of the Arabidopsis Root Science 2003 302 5652 1956 1960 10.1126/science.1090022 14671301

[B40] Scofield S Murray JA The evolving concept of the meristem Plant Mol Biol 2006 60 6 V VII 10.1007/s11103-006-0061-4 16724252

[B41] Maughan SC Murray JAH Bogre L A greenprint for growth: signalling the pattern of proliferation Current Opinion in Plant Biology 2006 9 5 490 495 10.1016/j.pbi.2006.07.010 16877026

[B42] Tessadori F Chupeau M-C Chupeau Y Knip M Germann S van Driel R Fransz P Gaudin V Large-scale dissociation and sequential reassembly of pericentric heterochromatin in dedifferentiated Arabidopsis cells J Cell Sci 2007 120 7 1200 1208 10.1242/jcs.000026 17376962

[B43] Raynaud C Sozzani R Glab N Domenichini S Perennes C Cella R Kondorosi E Bergounioux C Two cell-cycle regulated SET-domain proteins interact with proliferating cell nuclear antigen (PCNA) in Arabidopsis Plant J 2006 47 3 395 407 10.1111/j.1365-313X.2006.02799.x 16771839

[B44] Hjellstrom M Olsson ASB Engstrom P Soderman EM Constitutive expression of the water deficit-inducible homeobox gene ATHB7 in transgenic Arabidopsis causes a suppression of stem elongation growth Plant, Cell & Environment 2003 26 7 1127 1136 10.1046/j.1365-3040.2003.01037.x

[B45] Templeton GW Moorhead GB The phosphoinositide-3-OH-kinase-related kinases of Arabidopsis thaliana EMBO Rep 2005 6 8 723 728 10.1038/sj.embor.7400479 16065066 PMC1369146

[B46] Menand B Desnos T Nussaume L Berger F Bouchez D Meyer C Robaglia C Expression and disruption of the Arabidopsis TOR (target of rapamycin) gene PNAS 2002 99 9 6422 6427 10.1073/pnas.092141899 11983923 PMC122964

[B47] Francis D The cell cycle in plant development New Phytologist 1992 122 1 1 20 10.1111/j.1469-8137.1992.tb00048.x 33874038

[B48] Bernier G Growth changes in the shoot apex of Sinapis alba during transition to flowering J Exp Bot 1997 48 5 1071 1077 10.1093/jxb/48.5.1071

[B49] Jacqmard A Gadisseur I Bernier G Cell Division and Morphological Changes in the Shoot Apex of Arabidopsis thaliana during Floral Transition Ann Bot 2003 91 5 571 576 10.1093/aob/mcg053 12646501 PMC4242243

[B50] Svingen T Tonissen KF Hox transcription factors and their elusive mammalian gene targets Heredity 2006 97 2 88 96 10.1038/sj.hdy.6800847 16721389

[B51] Jasinski S Riou-Khamlichi C Roche O Perennes C Bergounioux C Glab N The CDK inhibitor NtKIS1a is involved in plant development, endoreduplication and restores normal development of cyclin D3; 1-overexpressing plants J Cell Sci 2002 115 5 973 982 11870216 10.1242/jcs.115.5.973

[B52] Zhou Y Wang H Gilmer S Whitwill S Keller W Fowke L Control of petal and pollen development by the plant cyclin-dependent kinase inhibitor ICK1 in transgenic Brassica plants Planta 2002 215 2 248 257 10.1007/s00425-002-0752-2 12029474

[B53] Wang H Zhou Y Gilmer S Whitwill S Fowke LC Expression of the plant cyclin-dependent kinase inhibitor ICK1 affects cell division, plant growth and morphology The Plant Journal 2000 24 5 613 623 10.1046/j.1365-313x.2000.00899.x 11123800

[B54] Altschul SF Madden TL Schaffer AA Zhang J Zhang Z Miller W Lipman DJ Gapped BLAST and PSI-BLAST: a new generation of protein database search programs Nucleic Acids Res 1997 25 17 3389 3402 10.1093/nar/25.17.3389 9254694 PMC146917

[B55] Samson F Brunaud V Duchene S De Oliveira Y Caboche M Lecharny A Aubourg S FLAGdb++: a database for the functional analysis of the Arabidopsis genome Nucleic Acids Res 2004 32 Database D347 350 10.1093/nar/gkh134 14681431 PMC308868

[B56] Derelle E Ferraz C Rombauts S Rouze P Worden AZ Robbens S Partensky F Degroeve S Echeynie S Cooke R From the Cover: Genome analysis of the smallest free-living eukaryote Ostreococcus tauri unveils many unique features PNAS 2006 103 31 11647 11652 10.1073/pnas.0604795103 16868079 PMC1544224

[B57] Palenik B Grimwood J Aerts A Rouze P Salamov A Putnam N Dupont C Jorgensen R Derelle E Rombauts S The tiny eukaryote Ostreococcus provides genomic insights into the paradox of plankton speciation Proc Natl Acad Sci USA 2007 104 18 7705 7710 10.1073/pnas.0611046104 17460045 PMC1863510

[B58] Taylor JS Peer Y Van de Braasch I Meyer A Comparative genomics provides evidence for an ancient genome duplication event in fish Philos Trans R Soc Lond B Biol Sci 2001 356 1414 1661 1679 10.1098/rstb.2001.0975 11604130 PMC1088543

[B59] Raes J Rohde A Christensen JH Peer Y Van de Boerjan W Genome-wide characterization of the lignification toolbox in Arabidopsis Plant Physiol 2003 133 3 1051 1071 10.1104/pp.103.026484 14612585 PMC523881

[B60] Saitou N Nei M The neighbor-joining method: a new method for reconstructing phylogenetic trees Mol Biol Evol 1987 4 4 406 425 3447015 10.1093/oxfordjournals.molbev.a040454

[B61] Felsenstein J Evolutionary trees from DNA sequences: a maximum likelihood approach J Mol Evol 1981 17 6 368 376 10.1007/BF01734359 7288891

[B62] Jones DT Taylor WR Thornton JM The rapid generation of mutation data matrices from protein sequences Comput Appl Biosci 1992 8 3 275 282 1633570 10.1093/bioinformatics/8.3.275

[B63] Fitch WM Toward Defining the Course of Evolution: Minimum Change for a Specific Tree Topology Systematic Zoology 1971 20 4 406 416 10.2307/2412116

[B64] Felsenstein J PHYLIP: Phylogeny Inference Package (version 3.2) Cladistics 1989 5 164 166

[B65] Felsenstein J Confidence limits on phylogenies: An approach using the bootstrap Evolution 1985 39 783 791 10.2307/2408678 28561359

[B66] Henikoff S Henikoff JG Amino acid substitution matrices from protein blocks Proc Natl Acad Sci USA 1992 89 22 10915 10919 10.1073/pnas.89.22.10915 1438297 PMC50453

[B67] Bailey TL Elkan C Fitting a mixture model by expectation maximization to discover motifs in biopolymers 1994 Menlo Park, California: AAAI Press 7584402

[B68] Crooks GE Hon G Chandonia JM Brenner SE WebLogo: a sequence logo generator Genome Res 2004 14 6 1188 1190 10.1101/gr.849004 15173120 PMC419797

[B69] Jaillon O Aury JM Noel B Policriti A Clepet C Casagrande A Choisne N Aubourg S Vitulo N Jubin C The grapevine genome sequence suggests ancestral hexaploidization in major angiosperm phyla Nature 2007 449 7161 463 467 10.1038/nature06148 17721507

[B70] Velasco R Zharkikh A Troggio M Cartwright DA Cestaro A Pruss D Pindo M Fitzgerald LM Vezzulli S Reid J A high quality draft consensus sequence of the genome of a heterozygous grapevine variety PLoS ONE 2007 2 12 e1326 10.1371/journal.pone.0001326 18094749 PMC2147077

[B71] Nishiyama T Fujita T Shin IT Seki M Nishide H Uchiyama I Kamiya A Carninci P Hayashizaki Y Shinozaki K Comparative genomics of Physcomitrella patens gametophytic transcriptome and Arabidopsis thaliana: implication for land plant evolution Proc Natl Acad Sci USA 2003 100 13 8007 8012 10.1073/pnas.0932694100 12808149 PMC164703

[B72] Zhu F Massana R Not F Marie D Vaulot D Mapping of picoeucaryotes in marine ecosystems with quantitative PCR of the 18S rRNA gene FEMS Microbiology Ecology 2005 52 1 79 92 10.1016/j.femsec.2004.10.006 16329895

[B73] Szabados L Charrier B Kondorosi A de Bruijn FJ Ratet P New plant promoter and enhancer testing vectors Molecular Breeding 1995 1 4 419 423 10.1007/BF01248419

[B74] Clough SJ Bent AF Floral dip: a simplified method for Agrobacterium-mediated transformation of Arabidopsis thaliana Plant J 1998 16 6 735 743 10.1046/j.1365-313x.1998.00343.x 10069079

[B75] Bertrand C Bergounioux C Domenichini S Delarue M Zhou DX Arabidopsis histone acetyltransferase AtGCN5 regulates the floral meristem activity through the WUSCHEL/AGAMOUS pathway J Biol Chem 2003 278 30 28246 28251 10.1074/jbc.M302787200 12740375

[B76] Domenichini S Raynaud C Ni D-A Henry Y Bergounioux C Atmnd1-[Delta]1 is sensitive to gamma-irradiation and defective in meiotic DNA repair DNA Repair 2006 5 4 455 464 10.1016/j.dnarep.2005.12.007 16442857

[B77] Nikovics K Blein T Peaucelle A Ishida T Morin H Aida M Laufs P The Balance between the MIR164A and CUC2 Genes Controls Leaf Margin Serration in Arabidopsis Plant Cell 2006 18 11 2929 2945 10.1105/tpc.106.045617 17098808 PMC1693934

