# Supplementary material for: Genes of the most conserved WOX clade in plants affect root and flower development in Arabidopsis
Source: BMC Evol Biol. 2008 Oct 24;8:291. doi: 10.1186/1471-2148-8-291 (PMC2584047; doi:10.1186/1471-2148-8-291)
Supplement: Additional file 1 — Phylogenetic trees. Figure 1 : Maximum-likelihood tree of the WOX proteins from the 5 completely sequenced genomes of the green lineage: Arabidopsis thaliana (AT prefix), Oryza sativa (OS prefix), Physcomitrella patens (Ppa prefix), Ostreococcus tauri (OT prefix) and the three full-length proteins of Selaginella moellendorffii (Sm prefix). The significance of each node was tested using 1000 bootstrap replicates. Only bootstrap values above 50% are shown. The scale, at the right of the tree, indicates 0.2 substitution per site. Figure 2 : Parsimony tree of the WOX proteins from the 5 completely sequenced genomes of the green lineage: Arabidopsis thaliana (AT prefix), Oryza sativa (OS prefix), Physcomitrella patens (Ppa prefix), Ostreococcus tauri (OT prefix) and the three full-length proteins of Selaginella moellendorffii (Sm prefix). The significance of each node was tested using 1000 bootstrap replicates. Only bootstrap values above 50% are shown. Figure 3 : Maximum-likelihood tree of the WOX proteins from the WOX13 Orthology Group. The significance of each node was tested using 1000 bootstrap replicates. Only bootstrap values above 50% are shown. The scale, at the right of the tree, indicates 0.1 substitution per site. Species names, in alphabetic order, are: Afo: Aquilegia formosa; AT: Arabidopsis thaliana; Bna: Brassica napus; Bra: Brassica rapa; Ccl: Citrus clementina; Csi: Citrus sinensis; Cte: Citrus temple; Ees: Euphorbia esula; Ghi: Gossypium hirsutum; Gma: Glycine max; Gra: Gossypium raimondii; Han: Helianthus annuus; Hvu: Hordeum vulgare; Les: Lycopersicon esculentum; Lja: Lotus japonicus; Lsa: Lactuca sativa; Lvi: Lactuca virosa; Mdo: Malus domestica; Mtr: Medicago truncatula; OS: Oryza sativa; OT: Ostreococcus tauri; Ppa: Physcomitrella patens; Psi: Picea sitchensis; Pta: Pinus taeda; Ptr: Populus tremula × Populus tremuloides; Pvu: Phaseolus vulgaris; Stu: Solanum tuberosum; Vvi: Vitis vinifera; Zma: Zea mays. Figure 4 : Parsimony tree of the WOX prote [file 1471-2148-8-291-S1.pdf]

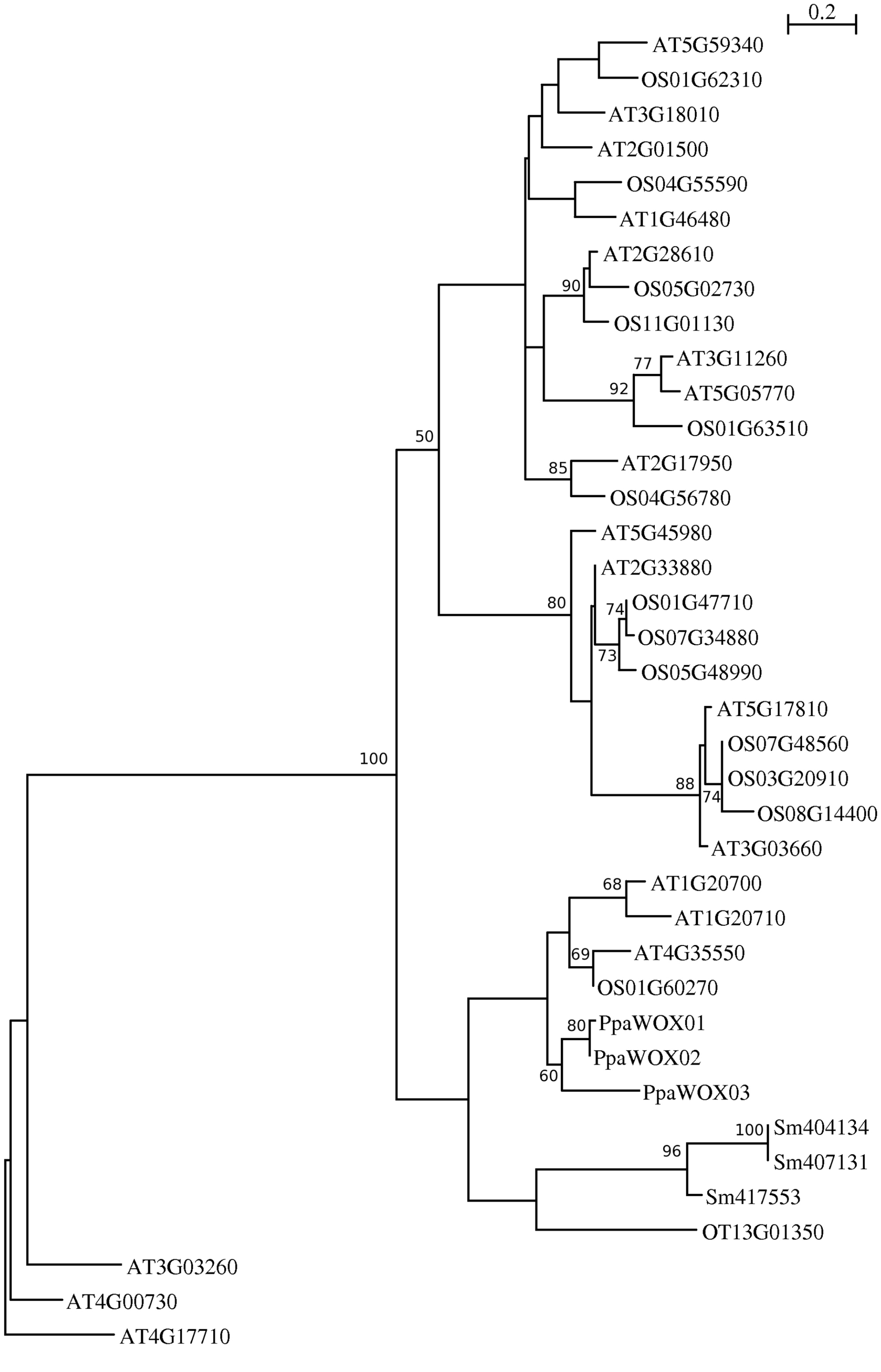

Figure 1: Maximum-likelihood tree of the WOX proteins from the 5 completely sequenced genomes of the green lineage

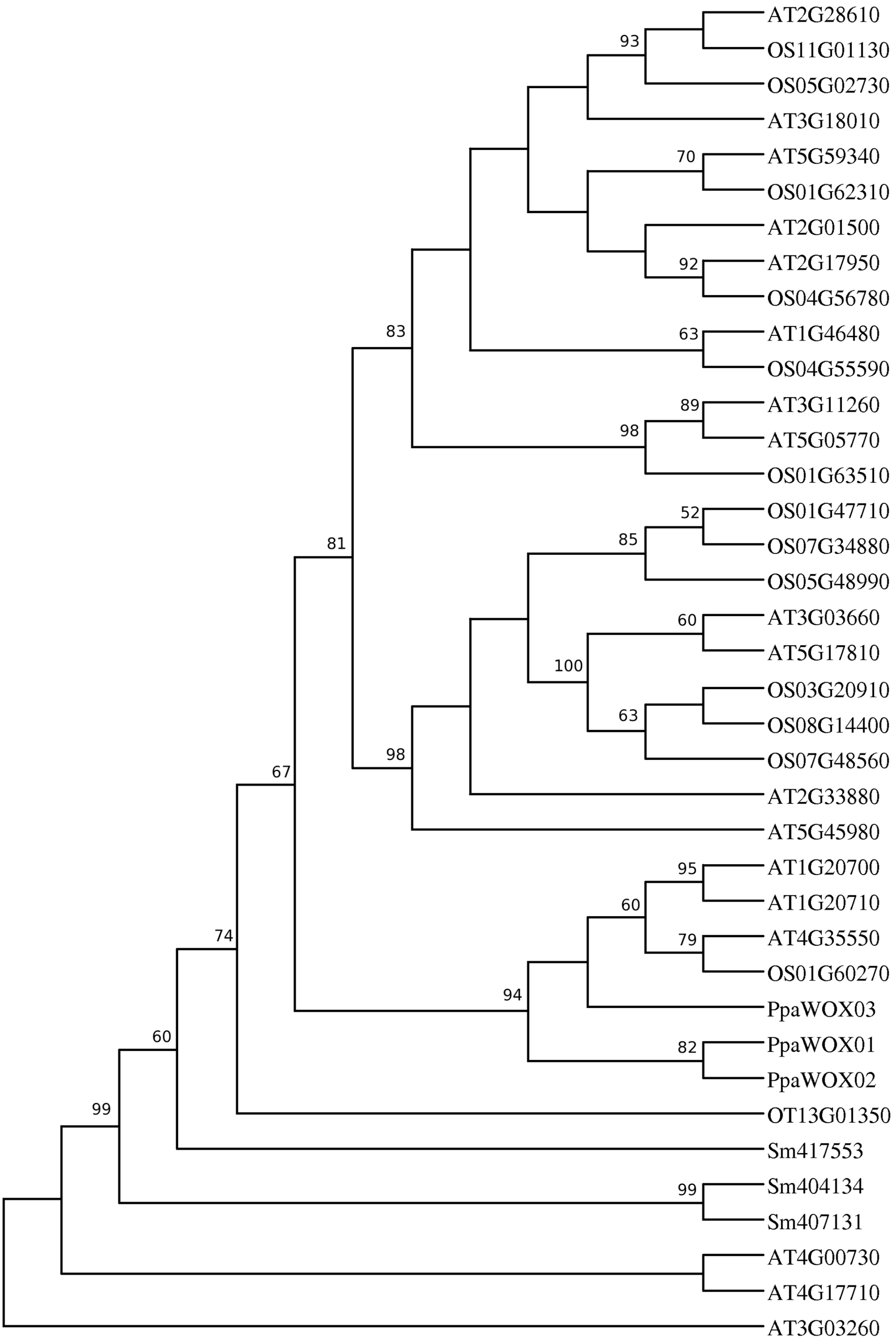

Figure 2: Parsimony tree of the WOX proteins from the 5 completely sequenced genomes of the green lineage

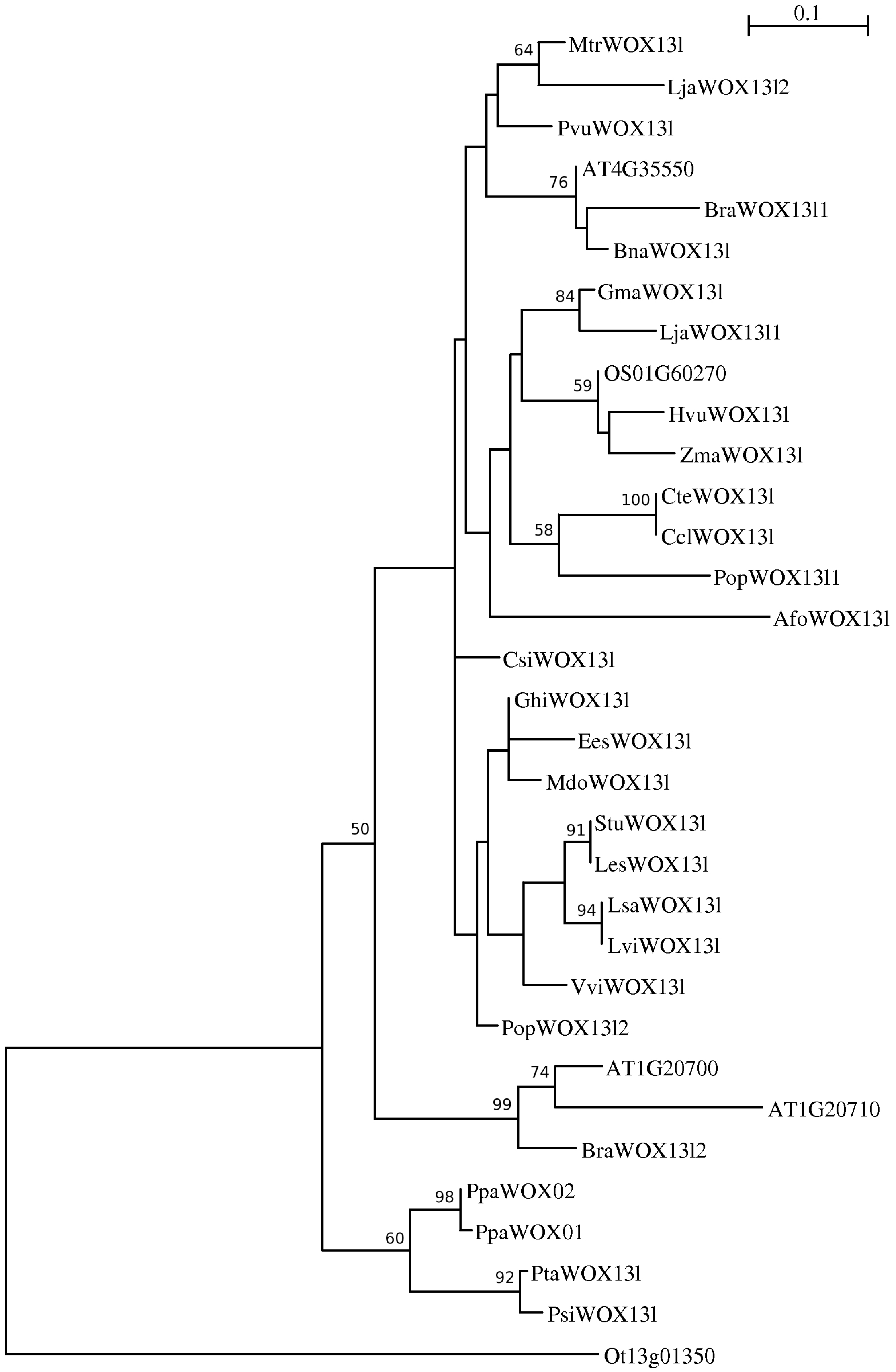

Figure 3: Maximum-likelihood tree of the WOX proteins from the WOX13 Orthology Group

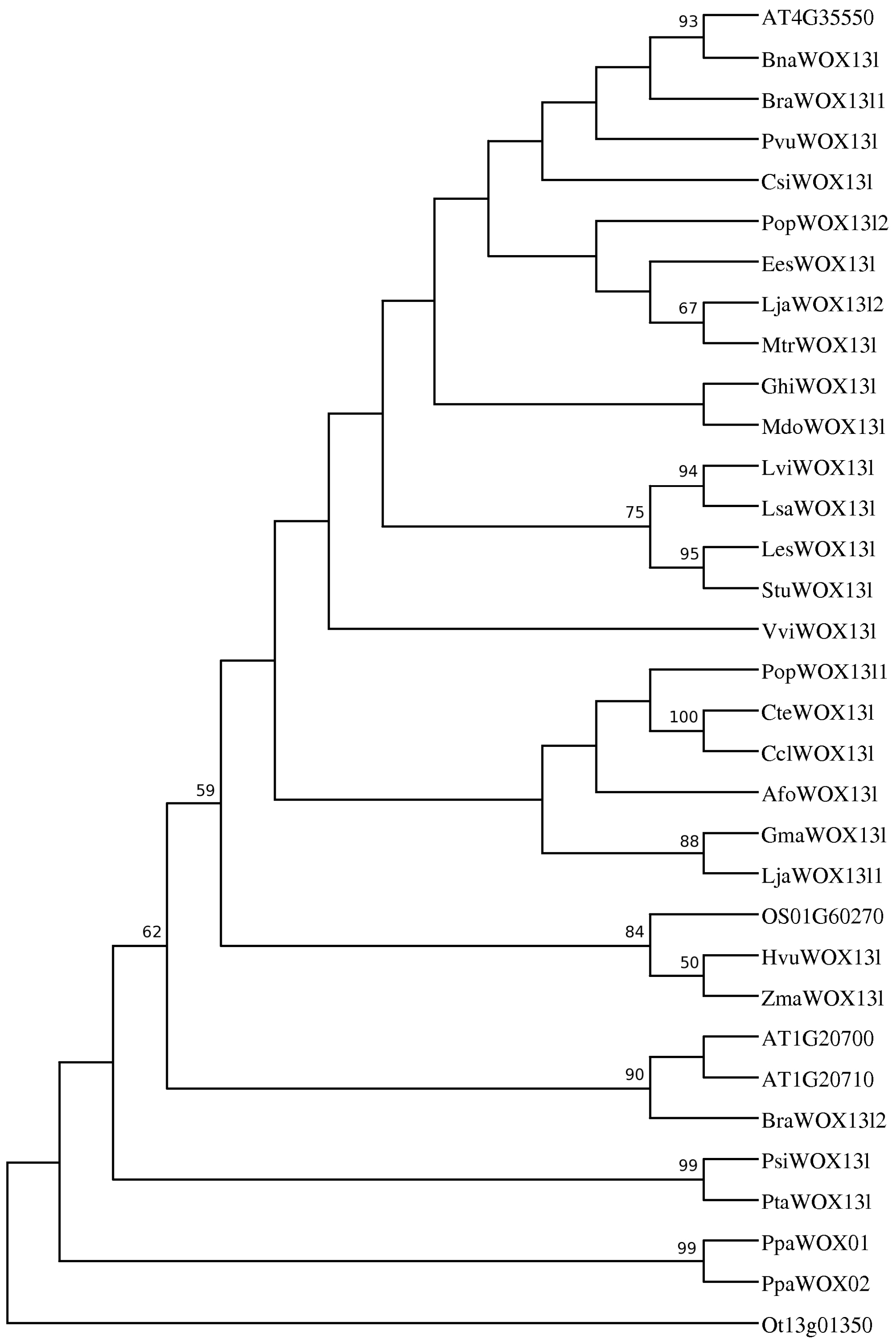

Figure 4: Parsimony tree of the WOX proteins from the WOX13 Orthology Group
