# Supplementary material for: Genes of the most conserved WOX clade in plants affect root and flower development in Arabidopsis
Source: BMC Evol Biol. 2008 Oct 24;8:291. doi: 10.1186/1471-2148-8-291 (PMC2584047; doi:10.1186/1471-2148-8-291)
Supplement: Additional file 2 — WOX gene structures and protein motifs. Figure 1 : The structure of WOX genes from model genomes. The gene structures from 4 model genomes (Arabidopsis thaliana, AT; Oryza sativa, OS; Physcomitrella patens, Ppa; Ostreococcus tauri, OT) and three Selaginella moellendorffii (Sm) genes are displayed following the order of the Neighbour-joining tree in Figure 1 and aligned on their translation start. Rectangles stand for exons while lines denote introns. Blue is for exonic regions in UTRs and light grey is for translated exons. Five different gene structures are given for PpaWOX02 due to the 5' UTR variants supported by transcripts. Figure 2 : WOX proteins: Sequence alignments around the virtual intron insertion sites. For each WOX OG, 15 amino acid sequences both upstream and downstream introns are given. The intron insertion sites are indicated by stars. Numbers on the left of sequences give the intron length. Digits between stars stand for the phase of the intron insertion site. The intron phase is defined by the position of its insertion into a codon. An intron can be located between two codons (phase 0) or within a codon, lying either after the first or after the second base pair (phase 1 and phase 2 respectively). Boxes indicate conserved introns with the same color code than in Figure 1. Figure 3 : Sequence logos of protein motifs in the WOX family. Each logo http://weblogo.berkeley.edu consists on stack of letters, one stack for each position in the sequence. The overall height of the stack indicates the sequence conservation at that position, while the letter's height within the stack indicates the relative information content of each amino acid at that position. The amino acid color code is dark blue for large polar, light blue for basic, black for small polar, green for nonpolar, orange for cysteine and purple for ambivalent amino acids. Figure 4 : WOX motifs: alignments for motifs without Logo. Motifs defined by less than 6 sequences cannot be shown as logo [file 1471-2148-8-291-S2.pdf]

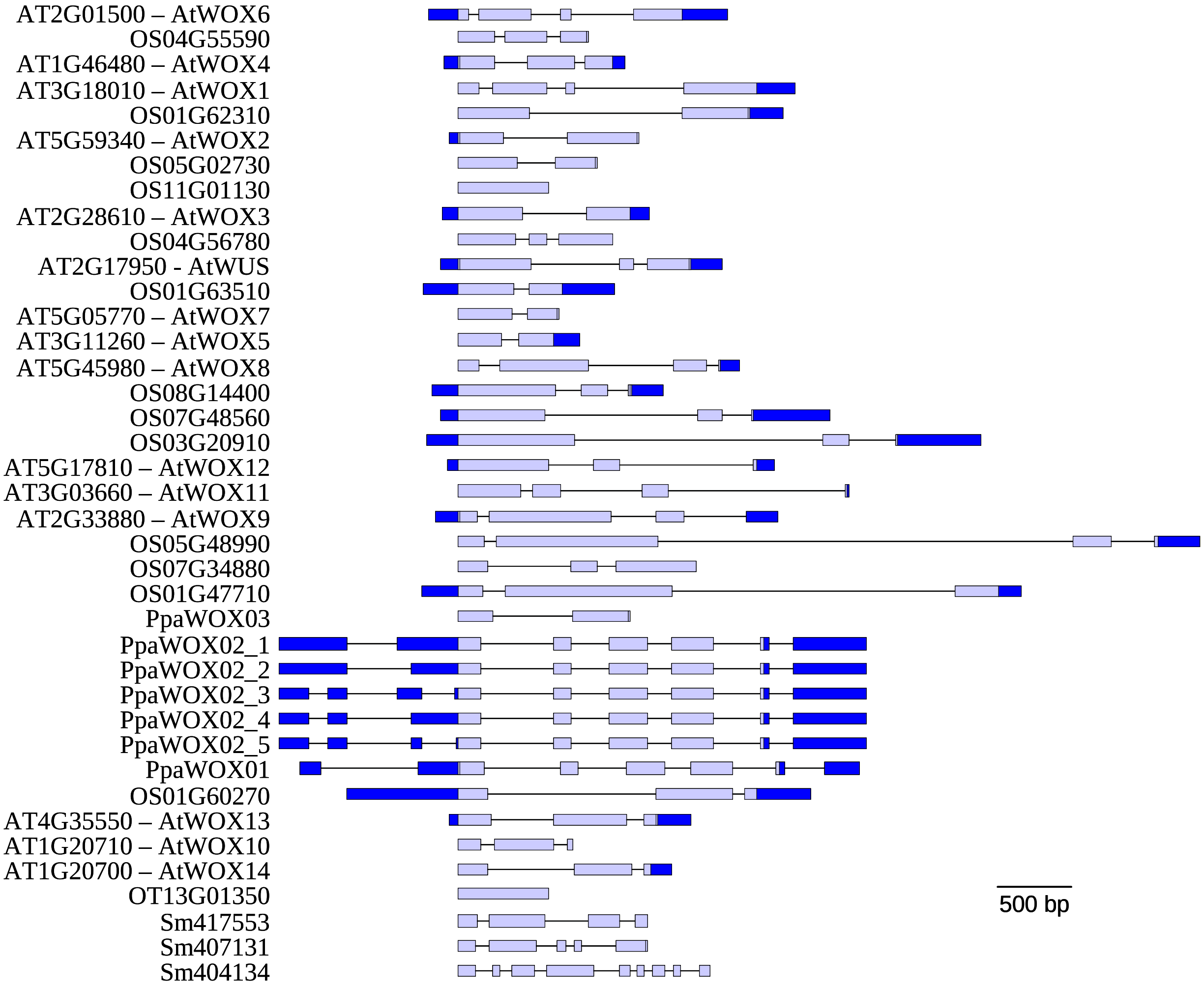

Figure 1: The structure of *WOX* genes from model genomes

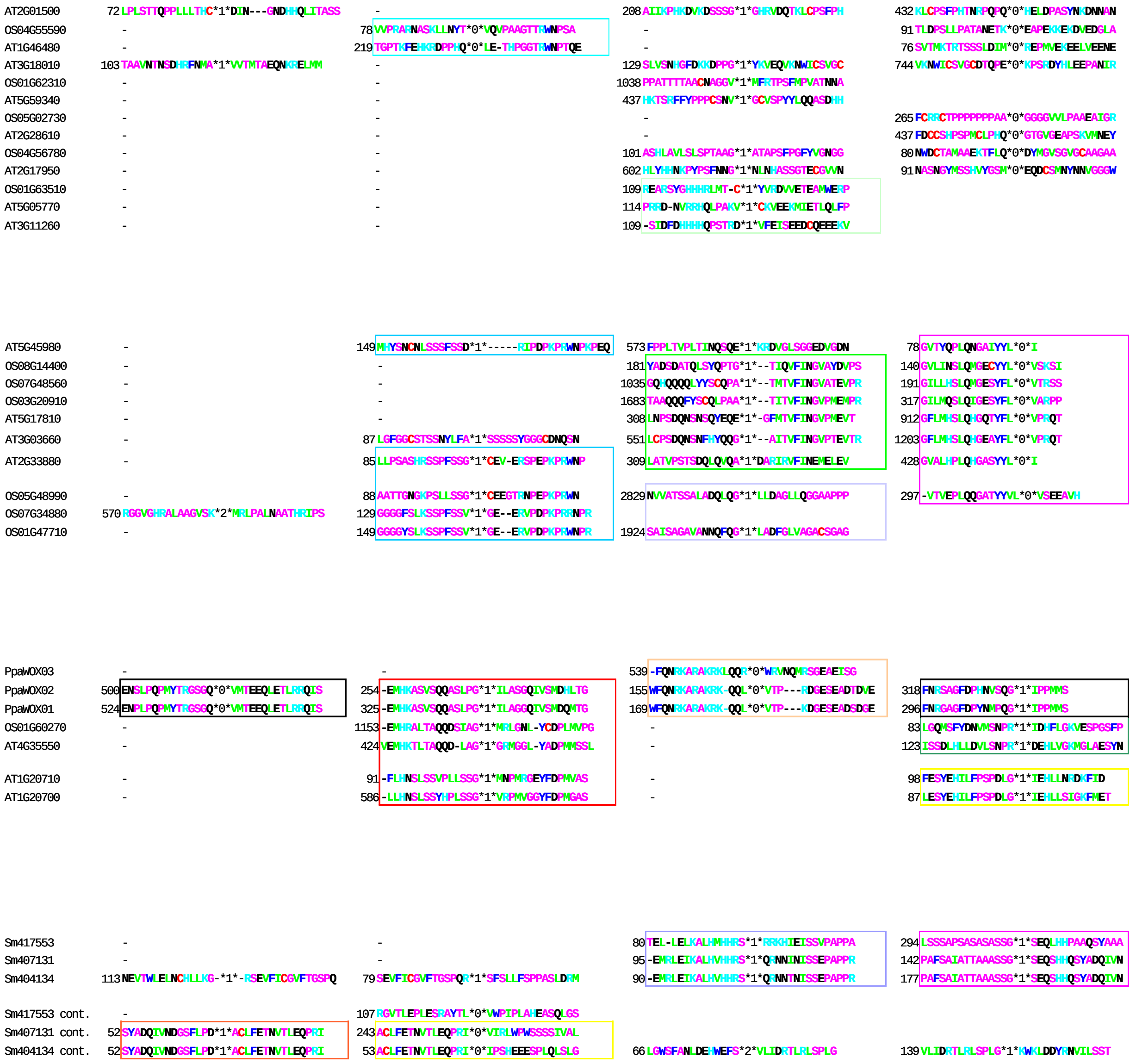

Figure 2: WOX proteins: Sequence alignments around the virtual intron insertion sites

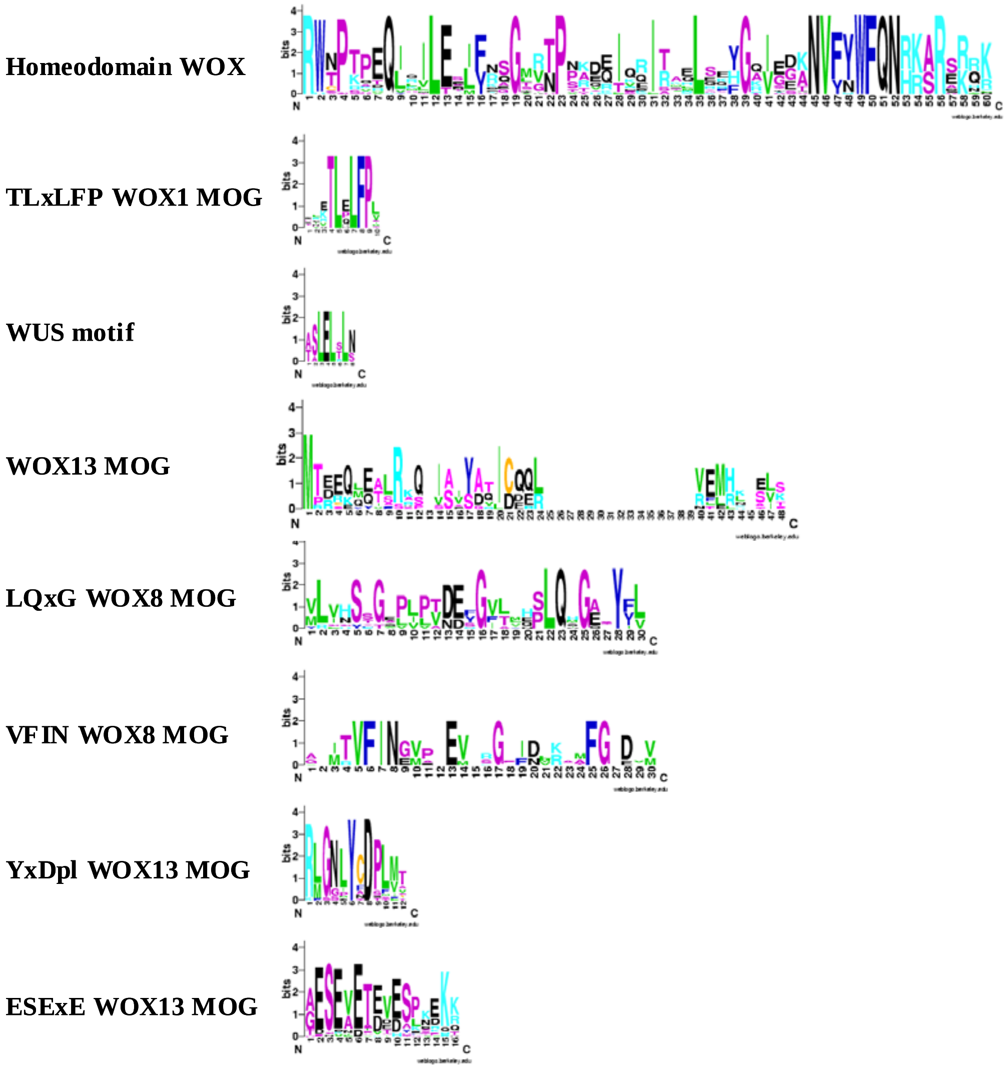

Figure 3: Sequence logos of protein motifs in the WOX family

Monocotyledon WOX13 MOG

```
HvuWOX13l     QS-MAMRAHNPDRISEMHRHFD
TtuWOX13l     QG-MAVRAHNPERISEMHRHFD
OS01G60270    QN-IAMRDHNSERISEMH-HFD
SbiWOX13l     QDHKAMGAHSADRISGMH-HFD
ZmaWOX13l     DNTKAMVALSADRVSGGMRHLD
consensus     q   AMrahn dRiSemhrHfD
```

daAVtt WOX10 MOG

```
BolWOX13l1    PQTMATTDQAEDAAVTTDEKRS
BraWOX13l2    PQTIATTDQAEDAAVTTDEKRS
AT1G20700     PQTTTANGQADDVAVTTEERRS
AT1G20710     PTTTITSSQADDAAVTTTEERG
BolWOX13l2    PQAMTTTTVQTVDAVAATEERS
consensus     Pqt  t  qa daAVtt E Rs
```

YFdPM WOX10 MOG

```
AT1G20710     GMNPMRGEYFDPMVASS
AT1G20700     GVRPMVGGYFDPMGASS
BraWOX13l2    GINPNGDGYFNPMVASS
consensus     G nPm ggYFdPMvASS
```

LxxGQ gymnosperm/moss WOX MOG

```
PtaWOX13l     LLMGQHMLYDLAQGNPGFRTSA
PsiWOX13l     LLMGQHMLYDLAQGNPGFRASA
PpaWOX02      LASGQIVSMDHLTGTPPHKSTA
PpaWOX01      LAGGQIVSMDQMTGTPTHKSTA
consensus     L  GQ    D   G P     A
```

QdxxxxLL gymnosperm/moss WOX MOG

```
PsiWOX13l     QDNDTPSLLPREDEMRPHASFGSTMIGVSGSAEHESVECLP
PtaWOX13l     QENDTQSLLQREDEMRPHASLGSTMLGVSGSAEHESVECLP
PpaWOX01      QDATSSALLHPQADTKPDISSFNRGAGFDPYNMPQGIPPMM
PpaWOX02      QDAASSALLHSQTDTKPDISSFNRSAGFDPHNVSQGIPPMM
consensus     Qd     LL       P  S      G
```

Figure 4: WOX motifs: alignments for motifs without Logo
