# Supplementary material for: Genes of the most conserved WOX clade in plants affect root and flower development in Arabidopsis
Source: BMC Evol Biol. 2008 Oct 24;8:291. doi: 10.1186/1471-2148-8-291 (PMC2584047; doi:10.1186/1471-2148-8-291)
Supplement: Additional file 3 — WOX sequences and HMM motifs. Table 1: Gene and protein sequences of the WOX family in the model genomes A. thaliana, O. sativa, P. patens and O. tauri. Table 2: Specificity and sensitivity of HMM for WOX motifs. Scores can give indications to distinguish potential family -or subfamily-members from false positive. A clear drop in the score could be detected in most cases, indicating that sequence below this threshold did not fulfill the family model as well as above [21]. HMMER bit scores were obtained for each motif by querying the HMM against three protein sequence sets: WOX13 OG (33 sequences), model genome WOX (32 sequences) and GenBank NR (Release 157). The score drops above 50 and indicating the motifs exhibiting the highest specificities with a given database are in bold. a: no hit was found in the set. b: no specific hit was found in the GenBank NR database. Note that Physcomitrella sequences from genome model WOX set are not present in GenBank NR. c: best hits are not belonging to the WOX family and no score drop was found. Table 3: Vitis vinifera WOX protein prediction with HMM WOX motifs. HMM search scores above a drop give indication to assign novel WOX proteins to the different WOX OGs. AM(...) IDs are contigs from Pinot noir V. vinifera while contig(...) IDs are from PN40024 V. vinifera. Dash indicates that no score was found above the drop. a: no hit was found with this HMM profile. (*): 17.4 score is due to an undefined C or T nucleotide at one position leading to a stop in the CDS. After fixing the nucleotide to C, the score is 27.7. [file 1471-2148-8-291-S3.pdf]

```
>AT5G59340
CATGCAAACCATCGTCTTAAAACCCTAGTTCTCCATAAAAAAAATATCCTTGAACACAAA
TAAATGGAAAACGAAGTAAACGCAGGAACAGCAAGCAGTTCAAGATGGAACCCAACGAAA
GATCAGATCACGCTACTGGAAAATCTTTACAAGGAAGGAATACGAACTCCGAGCGCCGAT
CAGATTCAGCAGATCACCGGTAGGCTTCGTGCGTACGGCCATATCGAAGGTAAAAACGTC
TTTTACTGGTTCCAGAACCATAAGGCTAGGCAACGCCAAAAGCAGAAACAGGAGCGCATG
GCTTACTTCAATCGCCTCCTCCACAAAACCTCCCGTTTCTTCTACCCCCCTCCTTGCTCA
AACGTGGGTTGTGTCAGTCCGTACTATTTACAGCAAGCAAGTGATCATCATATGAATCAA
CATGGAAGTGTATACACAAACGATCTTCTTCACAGAAACAATGTGATGATTCCAAGTGGT
GGCTACGAGAAACGGACAGTCACACAACATCAGAAACAACTTTCAGACATAAGAACAACA
GCAGCCACAAGAATGCCAATTTCTCCGAGTTCACTCAGATTTGACAGATTTGCCCTCCGT
GATAACTGTTATGCCGGTGAGGACATTAACGTCAATTCCAGTGGACGGAAAACACTCCCT
CTTTTTCCTCTTCAGCCTTTGAATGCAAGTAATGCTGATGGTATGGGAAGTTCCAGTTTT
GCCCTTGGTAGTGATTCTCCGGTGGATTGTTCTAGCGATGGAGCCGGCCGAGAGCAGCCG
TTTATTGATTTCTTTTCTGGTGGTTCTACTTCTACTCGTTTCGATAGTAATGGTAATGGG
TTGTAACGAAGGTTTAATGAAATTATAAATTTTATGTAA
>AT3G18010
ATGTGGACGATGGGTTACAACGAAGGAGGCGCAGATTCCTTCAACGGAGGAAGAAAGCTT
CGTCCTCTCATCCCACGTCTCACTTCTTGCCCCACCGCCGCCGTAAACACCAACTCCGAC
CACCGCTTTAATATGGCAGTGGTGACGATGACGGCGGAGCAGAACAAGAGGGAGCTGATG
ATGCTAAACTCAGAACCTCAACATCCACCGGTAATGGTGAGCTCACGGTGGAATCCGACA
CCAGATCAGTTAAGGGTTCTTGAAGAGCTTTACCGACAAGGAACTAGAACTCCTTCTGCC
GACCACATCCAACAAATCACCGCACAGCTACGACGGTACGGGAAGATAGAAGGCAAAAAC
GTTTTCTATTGGTTCCAAAACCACAAAGCCCGAGAACGCCAGAAACGACGGCGACAAATG
GAAACTGGCCACGAAGAAACGGTCCTTTCAACAGCAAGTCTTGTCTCAAACCACGGATTC
GACAAGAAAGATCCTCCAGGTTACAAGGTTGAACAGGTCAAGAACTGGATATGTTCGGTC
GGATGCGACACGCAACCAGAGAAACCTTCCCGTGATTATCATCTGGAGGAGCCAGCGAAT
ATACGGGTGGAGCACAATGCTCGTTGTGGAGGAGACGAGAGACGAAGCTTTTTAGGGATA
AACACCACGTGGCAGATGATGCAGTTGCCACCTAGTTTCTACTCTTCTTCACATCATCAT
CATCAACGTAACCTCATTCTCAATTCACCTACTGTTTCCTCCAACATGTCCAACAGCAAC
AATGCTGTCTCTGCTTCTAAAGATACGGTCACGGTTTCACCTGTATTTCTACGTACGAGA
GAAGCAACGAACACAGAGACTTGTCATCGAAATGGTGATGATAATAAAGATCAAGAACAA
CATGAAGATTGCTCCAACGGTGAATTGGATCATCAAGAACAGACACTTGAGCTGTTTCCA
CTGAGAAAAGAAGGGTTTTGTAGCGATGGTGAGAAAGACAAGAACATTAGTGGCATTCAC
TGTTTCTATGAGTTTCTGCCATTGAAGAACTAAACCTAATTGAAGTCTTTCAAGAAAAAC
AGCTTTTTGTTATTAGATGATTAGTTTAATTATTCAACCGAGTTAGGGAGAGTGAAAAAG
GATAATTAGGGTTTTGTCTAGGAGTAAGGGCAGGCGTGTCTTTATGGTGTTGATGTTAAC
ATTTCATAAGGGTTTGCCTTAAAATGCACTTTGGACCACTTTCTTTAATTGTTAAAAATG
AACCAAACAAATTTAAAAAGGAAAAGAATAGAAAGACATGTGATTTTCCC
>AT2G28610
ATCCACACACACACACATCTCTCTCTATATAACCCTAAGTGTTTGGAGATAGCATCACTA
GAGAACACACACACACAAAGCATCAATATTTGTCTGAACGGAGAATGAGTCCTGTGGCTTCA
ACGAGGTGGTGTCCGACGCCGGAGCAACTGATGATCTTGGAAGAGATGTACCGGAGTGGT
ATACGGACTCCGAATGCGGTGCAGATACAACAGATCACAGCTCACTTGGCGTTCTATGGA
AGAATCGAGGGCAAAAACGTCTTTTACTGGTTCCAGAACCATAAGGCTAGAGATAGACAG
AAGCTGAGGAAGAAACTAGCCAAGCAACTTCACCAGCAACAACATCAACTTCAACTCCAA
CTTCAGCAGATCAAACCAAAACCAATATCATCGATGATTTCTCAACCAGTTAATAAGAAT
ATCATCGATCATCACAATCCTTATCATCATCACCATCATAATCATCATCATAATCATCAT
CGTCCATATGATCATATGTCCTTTGATTGCTGCTCTCATCCTTCTCCCATGTGTCTTCCT
CATCAGGGAACTGGAGTAGGAGAAGCTCCAAGCAAAGTGATGAATGAATATTACTGCACC
AAAAGTGGAGCTGAAGAGATATTGATGCAAAAATCAATCACGGGTCCAAACTCATCGTAC
GGTCGAGATTGGATGATGATGATGGATATGGGCCCACGACCATCATATCCCTCATCATCA
TCATCACCCATTTCATGTTGTAACATGATGATGAGCAGTCCAAAGATACCATTGAAAACC
CTGGAACTTTTCCCAATCTCATCTATCAACTCCAAACAAGACAGTACCAAACTTTAAAGA
AAAGACAACAAATACAAAAGAGATTCTGCAGGTGTTTCATTCATGTATCACTGTATGATG
TGTCAAAGGCTAAGAAGAAAACACGTGTACTTGAGGGTAAAGTAATAGCCGTTGGATATA
```

```
ATTCGT
>AT2G01500
AGCCTTTTCATCTCTCTTGCATCACAAGTAAACCTCTCATCATTCATTTCCACACAGCAC
TGAAACTCGAGCATTTGCTCTCTCGAATCCCATTTCCCTAGCTAAAAGGTTCGGAATTCC
ACAACAACAACAAAAGACCTATTTTGTGAATCTTTTCTCTAAATTAAGAGTTATTAGGGC
AAATTTCTACATAGCATGGGCTACATCTCCAACAACAACCTCATCAACTATTTGCCCCTC
TCTACTACTCAACCTCCTCTTCTTCTCACCCACTGTGATATTAACGGCAATGATCACCAT
CAGCTCATAACCGCATCATCAGGAGAACACGATATTGATGAACGGAAAAACAACATTCCT
GCGGCGGCGACTTTGAGATGGAATCCGACGCCAGAGCAGATCACGACGCTAGAAGAGCTT
TACAGAAGCGGAACACGGACGCCGACGACGGAACAGATCCAACAGATAGCATCTAAGCTT
CGTAAATATGGGAGAATCGAAGGGAAGAACGTTTTCTATTGGTTTCAGAATCATAAGGCT
AGAGAGAGACTAAAACGCCGCCGTCGTGAAGGTGGTGCTATTATCAAACCACATAAAGAC
GTCAAGGATTCATCATCAGGTGGTCATCGAGTTGATCAGACAAAGCTCTGCCCATCTTTT
CCACACACAAACCGACCACAGCCACAGCATGAATTAGATCCTGCGAGTTACAATAAAGAC
AACAATGCTAATAATGAAGATCATGGGACGACTGAAGAATCTGATCAGAGGGCATCAGAG
GTTGGTAAATACGCCACATGGAGAAATCTTGTTACTTGGTCGATAACTCAACAACCGGAA
GAGATTAATATCGACGAAAATGTCAACGGAGAAGAAGAAGAAACGAGGGACAACCGGACT
TTAAATCTCTTTCCGGTTAGGGAGTACCAAGAGAAAACAGGCCGGTTGATAGAGAAGACG
AAAGCATGCAACTACTGTTACTACTACGAGTTCATGCCTCTGAAGAACTGAAACAAAAAA
CCCATGTGTGAATAATAAATAGGACTTTAATGAAGGTTTTATTTGTTGTTTTGTCCACGA
ATGGTAAGTGATGTGTTCTTATAATAGAAGAGAAGCGTTTGAAGATGGAGACGTGTAGTA
AATGTTATATAGGAGCGTTTGAAGATTATTATTGTTAGTTATTACCCAAATGAGAAGGTG
GACCATGTTGGGGCTTGGGTTTGGAGTTGCAACTATCTATACATATGTCTGAAAGTGAAA
CTGAAAGCTTCGATGGTTCCTTGTCAATGTAAGGAATATATATTAATTAGATGTTTGTTA
GTAAT
>AT1G46480
TAACACTATCATTTCACACTTATCTTCCTCTATATATACATAGCTTAAGTCTTGTAACGA
GAAAGGCATGCATAGCATTTGCTAGTTTTAACATATAGCAATGAAGGTTCATGAGTTTTC
GAATGGGTTTTCTTCATCGTGGGATCAACATGACTCGACATCATCCCTTAGCCTAAGCTG
CAAACGCCTCCGTCCTCTCGCCCCTAAGCTCTCCGGCAGCCCTCCCTCCCCTCCTTCTTC
TTCCTCCGGCGTCACTTCAGCCACTTTTGACCTTAAAAACTTCATTAGACCCGATCAAAC
CGGTCCGACAAAATTTGAACACAAACGAGACCCTCCTCATCAATTGGAGACGCACCCGGG
AGGGACAAGGTGGAACCCGACTCAAGAACAGATAGGGATACTTGAGATGTTGTACAAAGG
TGGAATGCGTACTCCTAATGCTCAACAGATTGAGCATATCACATTGCAACTCGGTAAGTA
CGGGAAAATCGAAGGGAAAAATGTGTTCTATTGGTTCCAGAACCACAAAGCCCGCGAGAG
ACAGAAGCAGAAGAGGAACAACCTCATCAGCCTAAGTTGCCAAAGCAGCTTCACGACCAC
TGGTGTCTTTAATCCGAGTGTAACTATGAAGACAAGAACATCATCGTCACTAGACATTAT
GAGAGAACCAATGGTGGAGAAGGAGGAGTTAGTGGAAGAGAATGAGTACAAGAGGACATG
TAGGAGCTGGGGATTTGAGAACTTGGAGATAGAGAACAGGAGAAACAAAAATAGTAGTAC
TATGGCAACTACTTTTAATAAAATCATTGACAATGTAACCCTCGAGCTTTTTCCTCTCCA
TCCTGAAGGGAGATGAAGTCATGAAGGTGAGGCAGAAAATTGTGGAATTCTTATGTAGAT
CTGGTTTAGGTTCAGAGGAATCAATTGGTATC
>AT2G17950
AAAATCTCTTTACTACCAGCAAGTTGTTTTCTTGCTAACTTCAAACTTCTCTTTCTCTTG
TTCCTCTCTAAGTCTTGATCTTATTTACCGTTAACTTTGTGAACAAAAGTCGAATCAAAC
ACACATGGAGCCGCCACAGCATCAGCATCATCATCATCAAGCCGACCAAGAAAGCGGCAA
CAACAACAACAACAAGTCCGGCTCTGGTGGTTACACGTGTCGCCAGACCAGCACGAGGTG
GACACCGACGACGGAGCAAATCAAAATCCTCAAAGAACTTTACTACAACAATGCAATCCG
GTCACCAACAGCCGATCAGATCCAGAAGATCACTGCAAGGCTGAGACAGTTCGGAAAGAT
TGAGGGCAAGAACGTCTTTTACTGGTTCCAGAACCATAAGGCTCGTGAGCGTCAGAAGAA
GAGATTCAACGGAACAAACATGACCACACCATCTTCATCACCCAACTCGGTTATGATGGC
GGCTAACGATCATTATCATCCTCTACTTCACCATCATCACGGTGTTCCCATGCAGAGACC
TGCTAATTCCGTCAACGTTAAACTTAACCAAGACCATCATCTCTATCATCATAACAAGCC
ATATCCCAGCTTCAATAACGGGAATTTAAATCATGCAAGCTCAGGTACTGAATGTGGTGT
TGTTAATGCTTCTAATGGCTACATGAGTAGCCATGTCTATGGATCTATGGAACAAGACTG
TTCTATGAATTACAACAACGTAGGTGGAGGATGGGCAAACATGGATCATCATTACTCATC
TGCACCTTACAACTTCTTCGATAGAGCAAAGCCTCTGTTTGGTCTAGAAGGTCATCAAGA
```

AGAAGAAGAATGTGGTGGCGATGCTTATCTGGAACATCGACGTACGCTTCCTCTCTTCCC
TATGCACGGTGAAGATCACATCAACGGTGGTAGTGGTGCCATCTGGAAGTATGGCCAATC
GGAAGTTCGCCCTTGCGCTTCTCTTGAGCTACGTCTGAACTAGCTCTTACGCCGGTGTCG
CTCGGGATTAAAGCTCTTTCCTCTCTCTCTCTCTTTCGTACTCGTATGTTCACAACTATG
CTTCGCTAGTGATTAATGATGCAGTTGTTATATTAGTAGTTAACTAGTTATCTCTCGTTA
TGTGTAATTTGTAATTACTAGCTAAGTATCGTCTAGGTTTTAATTGTAATTGACAACCGT
TTTATCTCTATGATGAATAAGTTAAA
>AT3G11260
CGTAAAACAGTTGAGGACTTTACATCTGAACATGTCTTTCTCCGTGAAAGGTCGAAGCTT
ACGTGGCAACAATAACGGAGGAACGGGGACGAAGTGCGGGAGATGGAATCCAACGGTGGA
GCAGTTGAAGATATTGACTGATCTGTTTCGAGCCGGTCTTAGAACTCCAACAACTGATCA
GATTCAGAAGATCTCTACGGAGCTCAGTTTCTACGGCAAGATAGAGAGCAAGAATGTTTT
CTATTGGTTTCAGAATCATAAGGCTAGGGAGAGGCAGAAACGTCGTAAAATCTCCATTGA
TTTTGATCATCATCATCATCAACCATCAACTAGAGATGTTTTTGAAATAAGCGAAGAAGA
TTGTCAAGAGGAAGAGAAGGTGATAGAGACATTACAACTCTTTCCGGTGAATTCATTTGA
AGACTCCAACTCCAAGGTGGACAAAATGAGAGCTAGAGGCAATAACCAGTACCGTGAATA
TATTCGAGAGACCACCACGACGTCGTTTTCTCCATACTCATCATGTGGAGCTGAAATGGA
ACATCCACCGCCATTAGATCTTCGATTAAGCTTTCTTTAAGTCATTGACCACAATAACAA
AAGAAAAAAAACAGATGCTTTAGCCTTTAAAATGTTGTTGTATTTTGAATTATGACATAT
TATCCGATGCATGGTTTATGAGATATTTTCCAATGCATGGTTTATCAAAAAGTTGTCTCA
TTTTGTTTGATATAAGAATCTATGTTATGG
>AT5G05770
ATGTCGTCGAGAGGATTCAACATTAAAGCTAGAGGATTATGTAATAACAACAACGGAGGA
GGAGGAACGGGGGCGAAGTGTGGACGGTGGAATCCAACGGTGGAGCAAGTGAAGCTTCTG
ACAGATCTGTTCAAGGCGGGACTGCGAACACCGAGCACGGACCAGATTCAGAAGATCTCT
ATGGAGCTGAGTTTCTACGGTAAGATTGAGAGCAAGAACGTGTTCTATTGGTTCCAAAAC
CATAAAGCTAGAGAGAGACAAAAGTGCCGGAAAATCTCCACCGTCAAGTTTGATCATCGT
CAAGATACAGATCTTTCTAAGCCTCGCCGAGACAACGTACGTCGTCATCAACTACCAGCG
AAAGTTTGTAAAGTAGAAGAGAAAATGATAGAGACGTTGCAACTCTTCCCATTATCGAAA
GTTGAGAGAGTGAGAGCAAACGTTACTGCTGCGAGCCACAACGAATACACACGAGAGCAA
GCATATACGACGGCGTTTTCTACATTCTCAACATGTGGGGCTGAGATGGAACATTCGCCG
TTGGATCTCCGATTAAGCTTTCTATGA
>AT4G35550
GTTTCCTTCGTTCACACAGAGAAAGAGAGAAATCTTCTGAGCGAAATTCTGACAAAAGAG
ATGATGGAATGGGATAATCAGCTACAACCCAATAACCATCACTCTTCTAATCTTCAAGGG
ATCGACGTTAATGGCGGCTCCGGCGCCGGAGGAGGAATGTACGTGAAGGTGATGACCGAT
GAGCAGTATGAAACTCTCAGGAAACAGATTGCTATTTACGGCACCATTTGTGAGCGTCTT
GTTGAGATGCATAAAACTCTCACTGCTCAACAAGATCTTGCAGGAGGGAGAATGGGAGGA
CTATATGCAGACCCAATGATGTCATCTTTAGGTCATAAGATGACAGCTAGACAGAGGTGG
ACTCCTACGCCAGTCCAGCTTCAGATTCTGGAACGTATATTCGATCAAGGCACGGGAACA
CCGAGCAAGCAGAAGATCAAAGACATAACAGAAGAGCTGAGCCAACACGGCCAGATTGCT
GAACAAAATGTCTATAACTGGTTCCAGAACCGACGTGCTCGATCCAAGAGGAAGCAGCAT
GGTGGAGGTTCTTCTGGAAATAATAATGGTGAGTCTGAGGTAGAGACTGAAGTTGAAGCA
TTGAATGAGAAGAGAGTAGTGAGGCCAGAGAGTCTTCTTGGTCTTCCAGATGGAAATAGC
AACAATAATGGGTTAGGGACAACAACAGCAACAACTACTGCTCCTAGGCCTGAAGATCTT
TGCTTTCAGAGCCCTGAGATTAGCTCAGACCTTCACTTGCTAGATGTTCTATCAAACCCA
AGGGATGAGCATCTTGTTGGAAAGATGGGACTGGCGGAAAGTTACAACCTTTATGATCAT
GTTGAAGATTATGGCATGTCAGGCTGATTGAATACAAGCTGAGGAAACTTCTAATTATCA
GAGAGATCAATGAAATGGATTTGGTAGTCAGTCATAAGCAGTATTAGACTTTTGTTTTTC
TTAAATTGTTTCTTTCAGGGCTTATTAAAGAAACAAGTAGTTTATCTTATTAATCATTTT
CAGAGAAACATTTACATGGATTGTTGAATCGTCTTGTGATGCTTGCTTTGATAATTTCTG
CATTCAGAACGATTTTT
>AT1G20710
ATGGAGCAAGAGAGCCTAAACGGTAGGTATGGTAGTAGAGTAATGACGGATGAACAAATG
GAGACTCTTCGTAAGCAAATCGCCATTTACGCCGTTCTTTGCGACCAGCTCGTCTTCCTC
CACAACTCTCTCTCTTCTGTCCCTCTTCTTTCATCAGGAATGAATCCAATGAGAGGTGAG
TATTTTGATCCAATGGTGGCATCGTCAAGCGCTCATGGAATGTCGACTCGGCCTCGATGG

ACTCCTACGACAACGCAACTTCAGATTCTTGAGAACATTTACAAGGAAGGCAGTGGAACA
CCAAATCCGCGGAGGATTAAAGAGATCACGATGGAACTGTCTGAACATGGACAAATCATG
GAGAAAAATGTATACCATTGGTTTCAAAACCGACGAGCTCGGTCCAAACGAAAGCAACCT
CCGACAACGACAATTACCTCGAGTCAGGCGGATGATGCGGCCGTGACAACAACTGAGGAG
AGAGGGAGGTGTGGAGATGATTCTGGAGGGTTTGAGTCTTATGAGCATATACTGTTCCCG
AGTCCTGATTTAGGGATTGAGCATTTGTTGAATAGGGACAAGTTTATAGACTGA
>AT1G20700
ATGGTAAAAAAAAAAAAGGAAAAGGAGAAAAGCAAAGAAATAGAGGAGATGGATAGAGAG
ATCCAAAACGGTGCGTATAGTGGGAGAGTGATGACTGAGGAGCAGATGGAGATTCTCCGT
AAGCAGATCGCCGTTTACGCCGTTATTTGTGACCAACTCGTCCTCCTCCACAACTCCCTC
TCTTCTTACCATCCACTCTCATCAGGAGTGAGGCCAATGGTTGGCGGATACTTTGATCCG
ATGGGGGCATCGTCAAGTTCTCATAGGATATCGACTAGGCATCGGTGGACTCCGACTTCA
ACACAGCTTCAGATACTTGAGAGCATTTACGACGAAGGAAGTGGAACACCGAATCGACGG
AGGATTAGAGAGATCGCGACGGAGCTGTCTGAACATGGACAGATCACGGAGACAAATGTC
TACAATTGGTTTCAAAACCGGCGAGCTCGGTCCAAACGAAAGCAGCCTCAAACGACGACA
GCTAATGGTCAGGCTGACGATGTGGCGGTGACAACGGAGGAAAGGAGGAGTTGTGGAGAT
TCAGGGGGATTAGAGTCTTATGAGCATATACTCTTCCCAAGTCCTGACTTAGGGATTGAG
CATTTGTTGAGTATAGGGAAATTTATGGAGACTTAAAATTCGTATAGACTTCGATGAAGA
AGATCTCTAATCTATTCTGTTTTCTTTTACTCTGTTTTCATAAAATAGAATTGTGTGTGT
TTGTGTGTGACATTAACAACAAGTCTTCAATTTTATTATAATTTTGTGTTCCTCAACTT
>AT2G33880
ATTCACATTTTTATTTATCTTTCCATTTAGCCATTCTGTTCCCTGTCTCTTCCTCCTCTC
TTTTTGACACATCACATCATCATCACATCATCATTCAACATCAATCATCATCATATGCAT
ACACATACATCTGTGTTCTGCGGATCGAGTTAATTAGTTATGGCTTCTTCGAATAGACAC
TGGCCAAGCATGTTCAAGTCCAAACCTCATCCCCATCAATGGCAACATGACATCAACTCT
CCTCTCTTGCCTTCTGCTTCTCACCGATCTTCTCCTTTCTCTTCAGGATGTGAAGTGGAG
AGGAGTCCAGAGCCAAAACCAAGATGGAATCCAAAGCCAGAGCAGATTCGGATACTTGAA
GCAATCTTTAACTCCGGGATGGTGAATCCTCCAAGAGAGGAGATCAGGAGGATTAGGGCT
CAGCTTCAAGAATACGGCCAAGTCGGTGATGCTAACGTCTTCTACTGGTTCCAAAACCGT
AAGTCCCGTAGTAAACACAAACTCCGCCTCCTCCACAACCACTCCAAACACTCTCTCCCT
CAAACGCAACCGCAGCCGCAGCCGCAACCTTCGGCTTCCTCTTCCTCTTCCTCCTCCTCT
TCCTCCTCCAAATCCACCAAACCCCGAAAAAGCAAGAACAAGAACAACACTAATCTCTCT
TTGGGTGGTAGTCAAATGATGGGGATGTTTCCACCGGAACCGGCGTTTCTCTTCCCGGTC
TCCACTGTCGGAGGGTTTGAAGGTATCACCGTCTCATCCCAATTAGGGTTTCTCTCCGGT
GATATGATTGAGCAACAAAAACCGGCTCCAACGTGTACCGGACTCCTGCTGAGTGAGATC
ATGAACGGTAGTGTGAGTTATGGAACTCATCATCAACAACACTTGAGTGAGAAAGAAGTT
GAAGAAATGAGGATGAAGATGTTGCAACAGCCACAGACTCAGATTTGTTACGCTACCACT
AATCATCAAATAGCTTCTTACAACAACAACAACAACAACAATAACATCATGCTTCATATT
CCTCCCACTACTTCTACTGCCACCACTATTACTACTTCGCATTCTCTCGCTACTGTCCCA
TCAACTTCGGACCAGCTTCAAGTTCAAGCGGACGCACGAATAAGAGTTTTCATCAATGAA
ATGGAGCTTGAAGTGAGCTCAGGACCGTTCAATGTGAGGGATGCATTTGGGGAAGAGGTT
GTTCTGATTAATTCCGCGGGTCAGCCCATTGTCACCGATGAATATGGCGTCGCTCTTCAC
CCTCTTCAACACGGAGCCTCGTACTATCTGATCTAGTCGTGTGGGAGATTTGAGTTTGAA
GAAGAAATTAAGACCTGTCTCTTTCTTTCACCATCTCTCGTACGTAGGCTTAAATGTTAA
GATTTTATAAAGTATTGGTTTCAGTTACCTGTTGTGACGGTGTTTATGTATGAGTTTCGG
ACAACATTCACAAAACTCTCTCGTTAAATTGTTGACCAATAATATATGATGTGTGTTTCA
TTATT
>AT5G45980
TACACCATCATCATGTCCTCCTCAAACAAAAATTGGCCAAGCATGTTCAAATCCAAACCT
TGCAACAATAATCATCATCATCAACATGAAATCGATACTCCATCTTACATGCACTACTCT
AATTGCAACCTATCATCTTCCTTTTCCTCAGATCGGATACCAGATCCTAAACCGAGATGG
AATCCTAAACCGGAGCAGATTAGGATACTCGAATCAATCTTCAATTCCGGTACTATTAAC
CCACCTAGAGAGGAGATTCAAAGAATCCGGATCCGGCTTCAAGAATATGGTCAAATCGGT
GACGCAAACGTGTTTTACTGGTTTCAAAACCGGAAATCTCGAGCAAAACACAAGCTTCGT
GTTCATCACAAAAGCCCTAAAATGTCAAAGAAGGACAAGACGGTTATTCCTAGTACTGAC
GCTGATCATTGTTTTGGTTTTGTTAACCAAGAAACCGGATTATATCCGGTTCAAAACAAT
GAGTTGGTGGTAACCGAACCGGCCGGTTTTCTATTTCCGGTTCATAATGATCCGAGCGCT

GCTCAATCAGCGTTTGGTTTTGGCGATTTTGTTGTACCGGTGGTAACGGAAGAAGGGATG
GCATTCTCTACCGTTAATAACGGCGTTAATTTGGAGACTAACGAAAATTTTGATAAAATT
CCGGCGATCAATTTATACGGCGGAGATGGAAATGGCGGTGGAAATTGTTTTCCTCCTTTG
ACTGTTCCATTAACCATCAATCAATCTCAAGAAAAACGAGATGTAGGATTATCCGGTGGT
GAAGACGTCGGAGATAATGTTTATCCGGTGAGAATGACGGTGTTTATTAACGAGATGCCT
ATCGAAGTAGTGTCTGGATTATTCAACGTTAAGGCAGCTTTCGGAAACGATGCCGTTTTG
ATCAACTCGTTTGGCCAGCCTATTCTTACAGATGAATTTGGTGTTACTTATCAACCTCTC
CAAAATGGCGCAATCTATTATCTTATTTAGAAGATATTGAAAAGCAAATGTTATGGTGCT
ATGGATAAATATTAATATTATAATAAAAGATTTCTGCGATTTATTTAGTTATTAATTATA
TAAGAATTTCATTTCTTATCTTTTAAATTTATGAACAATTTACAGGAC
>AT3G03660
ATGGACCAAGAACAAACACCACATAGCCCAACCCGCCATAGTCGCTCACCCCCATCCTCC
GCCTCCGGTTCCACCTCAGCAGAACCGGTTCGGTCCCGATGGTCACCTAAACCGGAACAA
ATACTCATACTTGAGTCGATCTTCCACAGTGGTATGGTTAACCCTCCCAAAGAAGAGACG
GTAAGGATAAGAAAGATGCTCGAGAAATTTGGCGCGGTGGGAGATGCAAATGTCTTCTAT
TGGTTTCAAAACCGGCGGTCAAGGTCCCGTCGGAGACAGCGACAGCTACAGGCTGCAGCT
GCAGCAGCGGCCGCAACCACCAACACTTGTGACCAGACTATGATGGTGAGCAACAGCTTA
CCACATCATAGTGGGAGTGATTTGGGGTTTGGAGGTTGTAGCACTTCTTCTAATTACTTA
TTTGCTAGCTCTTCTTCTTCTTATGGTGGTGGATGTGATAATCAAAGCAATAGTGGCATG
GAGAATCTCTTAACAATGTCTGGCCAAATGAGTTACCATGAAGCTACTCATCATCATTAT
CAAAATCATAGCTCAAATGTCACATCGATTTTGTGCCCATCTGATCAAAACTCCAATTTT
CACTACCAACAAGGGGCTATAACGGTGTTTATAAACGGAGTTCCGACAGAAGTGACGAGA
GGAGGAATAGACATGAAAGCAACGTTTGGAGAAGATTTGGTTTTGGTGCATTCCTCAGGT
GTTCCTCTTCCTACTGATGAGTTTGGTTTTTTGATGCATAGCTTACAACATGGTGAAGCT
TATTTCCTGGTTCCAAGACAGACATGAACTGGC
>AT5G17810
ATCTCCTTTCTCTTTTTCTTAAAATCCATAATCCAACCAAAAGAAGAAAAAAAAACAAGA
ATCTTGATAGCAAATGAATCAAGAAGGTGCTTCACATAGCCCATCCTCCACTTCCACCGA
ACCAGTCCGGGCACGTTGGTCACCTAAACCGGAGCAAATCTTGATACTCGAATCCATCTT
CAACAGTGGTACTGTTAACCCACCAAAAGATGAAACGGTGAGGATAAGAAAGATGCTTGA
GAAATTCGGTGCTGTGGGAGACGCAAACGTCTTCTACTGGTTTCAAAACCGACGGTCAAG
ATCTCGCCGGAGACACCGGCAGCTTTTAGCAGCCACCACCGCAGCCGCCACCTCCATAGG
AGCTGAAGACCACCAGCACATGACGGCCATGAGCATGCATCAATATCCTTGCAGCAACAA
CGAGATTGATTTGGGGTTTGGAAGTTGTAGCAACTTATCAGCTAATTACTTCCTTAATGG
ATCGTCGTCATCTCAAATCCCTTCCTTTTTCCTCGGCCTCTCTTCTTCAAGTGGTGGGTG
TGAGAACAACAATGGTATGGAGAATCTCTTCAAAATGTATGGCCATGAATCTGATCATAA
TCATCAGCAGCAGCATCATAGCTCAAATGCTGCATCAGTTTTAAACCCATCTGATCAAAA
CTCCAACTCCCAATACGAACAAGAAGGGTTTATGACGGTGTTTATAAACGGAGTTCCTAT
GGAAGTAACAAAAGGAGCAATAGACATGAAAACAATGTTCGGTGATGATTCGGTGTTACT
TCATTCCTCTGGTCTTCCTCTTCCCACTGATGAGTTTGGTTTCTTGATGCATTCTTTACA
ACATGGACAAACTTATTTCCTGGTACCGAGACAGACATGAACTGGCTGTTTTATGTGGGA
ACATATACGGTCGATTGTTTTCTTTTTTACCTCTATTGATTTCGTGTCTATCGTGTATAA
TTAGATTACAAATAAGTAATAATAAAAAGATTAAAT

>OS04G56780
ATGGATCACATGCAGCAGCAGCAGCGGCAGCAGGTGGGTGGAGGGGGAGGAGAGGAGGTG
GCGGGGAGGGGTGGTGTGCCGGTGTGCCGGCCGAGCGGGACGAGGTGGACGCCGACGACG
GAGCAGATCAAGATCCTGCGGGAGCTGTACTACAGCTGCGGCATCAGGTCGCCCAACTCG
GAGCAGATCCAGCGGATCGCCGCCATGCTGCGCCAGTACGGCCGCATCGAGGGCAAGAAC
GTCTTCTACTGGTTCCAGAACCACAAGGCCCGCGAGCGCCAGAAGAAGCGCCTCACCACG
CTCGACGTCACCACCACCACCGCCGCCGCCGCCGACGCCGACGCCAGCCACCTCGCCGTC
CTCTCCCTCTCGCCTACAGCAGCTGGCGCGACGGCTCCCTCTTTCCCGGGCTTCTACGTC
GGCAATGGCGGCGCCGTGCAGACGGATCAGGCCAACGTCGTCAACTGGGACTGCACCGCC
ATGGCAGCCGAGAAAACCTTCCTGCAGGACTACATGGGCGTGAGCGGCGTCGGTTGCGCC
GCCGGGGCGGCCCCGACGCCGTGGGCGATGACGACGACGACTCGCGAGCCCGAGACGCTT
CCACTCTTCCCAGTCGTCTTCGTCGGCGGCGACGGCGCGCATCGTCACGCCGTCCACGGC
GGTTTCCCGTCCAACTTCCAGCGCTGGGGTTCTGCTGCTGCTACCTCCTACACCATTACG

GTCCAGCAGCATTTGCAGCAGCACAACTTTTACAGCAGCAGCAGCAGCCAGCTGCACAGC
CAGGATGGGCCGGCAGCAGGCACATCCCTGGAGCTCACTCTCAGCTCCTACTACTGCTCA
TGCTCACCTTACCCTGCAGGGTCCATGTGA
>OS04G55590
ATGAGGCTTCACCATCTGCATGTGGCCTACCTGGATCACAAAGCGTCGTCGTCCTCGTCG
TCTCCGGCGCCACCATCCATCTCCCCGTCGTCGATCCCCGGATCGGCCGCCTTCCCTGCT
TTCTCCTTCAAATGCCTCCGCCCGCTCGCGCCCAAGATCTCGCTGCCGGAGCCGAGGAAG
ATGATCGCGCCGCCGGACTTCGTCGTCCCTCGCGCCAGAAACGCTTCCAAGCTGCTCAAC
TACACTGTGCAGGTGCCGGCGGCGGGGACGACGAGGTGGAACCCGTCGGCGGAGCAGATA
AAGGTGCTGGAGATGCTGTACCGCGGCGGGATGCGGACGCCGAACTCCGTCCAGATCGAG
CGGATCACGGAGGAGCTCGGCAAGTACGGCCGGATCGAGGGCAAGAACGTCTTCTACTGG
TTCCAGAATCACAAGGCCCGCGAGCGCCAGAAGCAGAAGCGCGCCGCCCTCCTCACCCTC
AGCACGCTCGACCCTTCCTTGCTGCCCGCTACCGCTAACGAAACCAAAGAGGCACCGGAG
AAGAAGGAGAAGGACGTGGAAGATGGATTGGCGAGCTGCAAGCGGCGGTGCAAGGCGTGG
GGTGACGGCGCCGGCGATGGAGACGCGGTGGTGGCGACGGAGGCGGCCGGCGGCTGCACC
GACGAAGTGACCCTGGAGCTCTTCCCGTTGCATCCCCAGGGAAAAGCTTAA
>OS11G01130
ATGCCTCAGACCCCTTCGACGCGGTGGTGCCCGACGCCGGAGCAGCTGATGATCCTGGAG
GAGATGTACAGGAGCGGCGTGCGAACGCCCAACGCGGCAGAGATCCAGCAAATCACGGCG
CACCTCGCCTACTACGGCCGCATCGAGGGCAAGAACGTCTTCTACTGGTTCCAGAACCAC
AAGGCCCGCGAGCGCCAGCGCCTCCGCCGCCGCCTCTGCGCGCGGCACCAGCAGCAACCC
TCACCGCCCTCCTCCACGGTGCCTCCGGCTCCCACTGCTGCTGCTGCCGGTGCCGTCGTG
CAGGTGCACCCCGCGGTGATGCAGCTACACCACCACCACCACCACCATCACCCATACGCT
GCGGCCGCCGCTGCCCAAAGTCATCACCTGCAGCAGCAGCAGCAGCAGCAAGCTGAGTGG
CCGGCGGCGGTGGACTACTGCAGCACTGCATCGGCGTCAGCGTCGGCAACTGCTGCTGAC
ATGGCGATCCCGCCGTGCTGCCGGCCGCTGAAAACGTTGGAGCTGTTCCCGACCAAGAGC
ACCAGCGGCGGCCTCAAGGAAGATTGCTGCAGCAGCTCCAAGTCCTCCTCTTGCTCCACC
TCCACCAATTAA
>OS05G02730
ATGGCGCCGGCGGTGCAGCAGCAGCAGAGCGGCGGCGGCGGCGGATCGACGGGGGCGGCG
GCGGTGGGGTCGACGACGCGGTGGTGCCCGACGCCGGAGCAGCTGATGATGCTGGAGGAG
ATGTACAGGGGAGGGCTCCGGACGCCGAACGCGGCGCAGATACAGCAGATCACGGCGCAC
CTCTCGACGTACGGCCGCATCGAGGGCAAGAACGTCTTCTACTGGTTCCAGAACCACAAG
GCCCGCGACCGCCAGAATGCCGCCGCCGCCTCTGCATCTCCCACCACCTCCTCTCCTGCG
CCCACTACTACCACCACCACCTCGCCGCCGCCGCCGCCGTCGTTCCGCCGCCGCAGCTTC
TGCCGCCGCTGCACCCCTCCTCCTCCTCCTCCTCCTGCGGCGGGAGGCGGCGGCGTGGTG
TTGCCGGCGGCGGAGGCGATCGGGCGTTCGTCGTCGTCGTCGGACTACTCGCTGGGGAAG
CTAGTGGACAACTTCGGGGTGGCGCTGGAGGAGACGTTCCCGGCGCAGCCGCAGCAGCCG
GCGACGACGATGGCGATGACGGCCGTCGTCGACACTACGGCGGTGGCGGCGGCGGCAGGT
GGCTTCTGCCGGCCGCTCAAGACGCTGGACCTCTTCCCCGGCGGCCTCAAGGAAGAGCAG
CATGACGTCGTCTAG
>OS01G63510
AGCATTGCTTCTGATCAGAAGAAGATCCCGGAAGAGACCAAATCAGAAGCTAAACCTATC
TACCACAAGGCCAAGTTACGTTAGACCGTTTTGATCAGTGAATTAGCTTTCAAGCAGTTC
TACTGCTCCCAGCCGCTCGATCGATCGATCGATCAAACAAGGTAGCTAGCTAGCTCAGGA
TCAAATCTGGTTTGTCTACATATATAGGTAGTGATCGATCAGTCGGTCGGTCATGGAGGC
TCTTAGCGGGCGAGTGGGGGTGAAGTGTGGGCGGTGGAACCCGACGGCGGAGCAGGTGAA
GGTCCTGACGGAGCTGTTCCGAGCGGGGTTGCGGACGCCGAGCACGGAGCAGATCCAGCG
CATCTCCACTCACCTCAGCGCATTCGGCAAGGTGGAGAGCAAGAACGTCTTCTACTGGTT
CCAGAACCACAAGGCCCGCGAGCGCCACCACCACAAGAAGCGCCGCCGCGGCGCCTCCTC
CCCCGACAGCGGCAGCAACGATGACGATGGCCGCGCCGCAGCCCACGAGGGCGACGCCGA
CCTCGTCCTGCAGCCTCCTGAGAGCAAGCGGGAGGCCAGAAGCTACGGCCATCATCACCG
GCTCATGACATGCTACGTGAGGGATGTGGTGGAGACGGAAGCGATGTGGGAGCGGCCGAC
GAGGGAGGTGGAAACGCTGGAGCTGTTCCCACTCAAGTCGTATGACCTCGAGGTGGACAA
GGTCCGGTATGTGAGGGGCGGCGGTGGCGAGCAGTGCAGGGAGATCTCGTTCTTCGACGT
CGCTGCCGGCCGGGATCCGCCGCTGGAGCTCAGGCTTTGCAGCTTCGGCCTCTAGATCGA
CCCGTGCATTGATGTGCCGCACATATGGCATGAGATGCAGCTTGCCGCGACTGTACCTGG

CCTCTTGTCGCTGTGCCTAGTCCTAGTTCTAGCTGCCCCACATCAAAAGGAGTACGTACG
AGATGTCATGAGATACGGCATGTGTTGTGGGAAAGTCCGTTGTTGCATGCAGTATCTGGG
AGGATCAGGAAAGAGCACGCAAACGAGATTTCCCCATTGCCCTTTTCCTTGGTCTTTCCA
TCATGAGATGTACTTCTACTTTTCTTCTTTTCTGCTACATGGATACTAACATGGTCACTG
CTGATACTTCTATATGATATATCCATTAATTAATATTCCATATTCCTTTTT
>OS01G62310
ATGGAGACGACGACGACGACGTTGGGCGGCGGCGGCGGCGGGCGGGCGGGAGGCTTCTCC
GATCCGCCGTCTCCGCTCTCGCCGCCGCTGTCGCCGGCCTCGGCGGCGGCGGCGGCGCTG
GCGAACGCGCGGTGGACGCCGACCAAGGAGCAGATCGCGGTGCTGGAGGGGCTGTACCGG
CAGGGGCTGCGCACGCCGACCGCCGAGCAGATACAGCAGATCACGGCGAGGCTCCGGGAG
CACGGCCACATTGAGGGCAAGAACGTGTTCTACTGGTTCCAGAACCACAAGGCCCGGCAG
CGGCAGAAGCAGAAGCAGCAGAGCTTCGACTACTTCAGCAAGCTGTTCCGCCGCCCGCCG
CCGCTGCCCGTGCTCCACAGGCCACTCGCGCGGCCCTTCCCTCTCGCCATGGCGCCGACG
GCGATGCCACCGCCGCCGCCGCCGCCGGCGACGACGACGACGGCCGCATGCAACGCCGGT
GGTGTGATGTTCAGGACGCCAAGCTTCATGCCGGTCGCGACAAATAACGCCAGCTACTAC
CCGCAGCAGCAGACGCCGTTGCTGTACCCAGGGATGGAAGTGTGTCCGCACGACAAGTCC
ACGGCGCAGCCACCGGCCACCACCACCATGTACCTGCAGGCACCGCCGAGCAGCGCACAC
CTCGCGGCGGCGGCTGGCCGCGGCGCGGCGGAAGCGGAAGGCCATGGCCGCCGCGGCGGC
GGCGCCGGTGGGCGCGAGACCCTCCAGCTGTTCCCCCTGCAGCCCACCTTCGTGCTGCCG
GATCACAAGCCGCTCCGCGCCGGGAGCGCCTGCGCCGCCGTGTCCCCGACGACGCCGTCC
GCGTCCGCGTCGTTCTCGTGGGAGTCGGAGAGCTCGGACAGCCCCAGCAGCGAGGCGCCT
CCGTTCTACGACTTCTTCGGCGTCCATTCTGGAGGCCGCTGAGCGTGAAGGTGAGAGTGA
GTGAGCGTAGAGTGGAGTGGAGTGGAGTGGAAGTTGATATCTTCATGAGTGGACGAACCC
GTCGTGCTTTTTGAAACTTATCTGCATTGTTGCATCGATCATTGCATGGTTTAATTGGTT
AAGGGATTGGAAGGTGTACTGCTTCATCAACTTGAAGTGTTTCATTGGAATGGAATGATG
CATGTTTTGAGGCGGCTGTGCTCTCAC
>OS01G60270
GCCCAATACTGCTCCTACAAAGACACTGCCACATTCCTTCTCCTTCCTCCTGCATACTGT
CTCTCTCTCTCTCTCTATCTGATCAGCTGTTCCGCTTCCTCCTCTTCTTCTCTCCCCCAA
CCCCGTAGCCCTCCCACCCATCGAGCCAGGCGGCGGCGCGACGGCCGGTGGCTAAGGCGG
CGGCCGGTGCCAAAATCTCGAGCAATCGCGTCGCGCCGTCTGGCCGGCTTGCGACATCGC
TCGCCCGAGAGCGTCGTCGTCTTCGAGCTTGTGCCGCGCGGCGCCGTCGCCGCGTGGTCC
GAGGGCCCCGTGCCACCGCGTGCAGAGGGCAGAGGCCCATCGCCTCCTCCTCGAGCCACC
GCGCTGCCGTGTGCCGTCCCCCTTGTCGTCCTCGAGCCCCTGCGTCGCCGCCGGCCGCCG
GGGCTCCTCCTCCTCGTCCTCCTCGTCATCGACGCCCCAATCGGCACCGCCTCCTGGTCG
TTAAAACCATCCTCGGGCCTTTCGCCAATTGGAAGGCGCCATCTTTTCCGCATTCTTTCC
ATCCGGATTCCGGCCGCTGTTGCTGTCTGGCGGAATATCTGTTTCGCCAGATTTTCTTGC
GTTTCGATTTCTTTTTATTATTTTCCTGAGAGAGAGAGAGAGAGAGAGAGAGAGAGAGAG
AGAGAGAGAGAGAGAGAGAGGGTTCCTTCCATTTCGCTGCTGCGCCAAAGAACACAACTT
TCCCAATTCCTTTTCGCCTCCCTGTAGAGGCATGGAGTGGGACAAGGCCAAGGCGTCCTC
CGGCGAGGCGGTGGACGACAGGGGCGGAGGGGAAGGAGGGCTCGGGTACGTGAAGGTGAT
GACGGACGAGCAGATGGAGGTGCTCCGGAAGCAGATCTCCATCTACGCCACCATCTGCGA
GCAGCTCGTCGAGATGCACCGCGCCCTCACCGCGCAGCAGGACTCCATTGCAGGAATGAG
GCTTGGTAATCTGTACTGTGATCCTCTAATGGTTCCCGGAGGTCACAAGATCACAGCAAG
GCAGCGATGGACACCAACCCCAATGCAGTTGCAGATTCTTGAGAACATCTTTGACCAAGG
CAATGGAACACCAAGCAAGCAAAAGATAAAGGACATAACAGCAGAGCTCTCACAGCATGG
CCAGATCTCAGAGACAAATGTCTATAACTGGTTCCAGAACAGGCGGGCACGATCGAAACG
GAAGCAAGCTGCTTTACCAAATAATAATGCAGAATCTGAAGCTGAAGCGGATGAGGAGTC
CCCAACTGACAAAAAACCCAAATCAGATAGGCCGCTTCACCAGAACATAGCCATGAGAGA
TCACAATAGCGAAAGGATCTCGGAGATGCACCACTTTGACACGGAGCATGAGCAAATCCG
TCGTATGATGTATGCATCCAATGACAGTAGCTCGCGATCGTCAGGCAGTTTGGGCCAGAT
GTCCTTCTACGACAATGTTATGTCAAATCCAAGAATCGATCATTTCCTTGGTAAGGTGGA
GAGCCCCGGGAGCTTCCCCCACATGCGATCCGGTGAAAGCTTTGATATGTATTGATGACG
CGGCAATTCTGGATTCTGGTTACTAGGTTCGTTGCATTAGACACCATACTGTTTTCCCCT
GTAAAAAGTGAAGCTGGTAACTTGCCGTTCATTTTGCTTACTGAAATTGCGAAGTTTGCC
TTACGTCCACACGTTGGTTATCATGGTCTTGTCGACATTCTACGGAGAATAGCTAGTTCT
AGTGTAAACCTGTTCTGTTGCTGAATTAGCTGCAAGAAGCCGATAAATAGCACTCTGCAC

ACTTAGGTGTTAGGTGATTGTGTGGATATTTCTTGATGGTGAGTTTGTAACGATCTTCTT
TCACTTTGTCCTGAAGGCTGAATCGATCAGCGCTACAAATGAGTTAGTAGTTTTAAA
>OS01G47710
ATTCCATCCATCCATCCACCTATCCTAGCTAGGGAGATAAGAGGAACACTCCTAGCTCGT
CTACTCCTAGCTCTCTCGTCCAATCCGGCCGGTCCCTGTCGCCTCAAGGAGAATCATCTG
TGGCTAGCTATAGATCTTTCTCTCATCGACGAGCGCGAGCTGCTAGTATAATTAGGCTTG
TGTTGTTGCAGAGGGGAGCTGAGAGTTCATCGGGTTGAATCGATCGATCGGCAGGTTTGC
GGGCATGGCGTCGTCGAACAGGCACTGGCCGAGCATGTTCAGGTCGAAGCACGCCACGCA
GCCGTGGCAGACGCAGCCTGACATGGCCGGGTCGCCGCCCTCCCTCCTCTCCGGCTCCTC
CGCCGGCAGCGCCGGCGGCGGCGGCTACTCCCTCAAGTCGTCGCCCTTCTCGTCAGTGGG
CGAGGAGAGGGTTCCGGACCCGAAGCCGCGGTGGAACCCGCGGCCGGAGCAGATCCGGAT
CCTGGAGGCGATCTTCAACTCCGGCATGGTCAACCCGCCGCGCGACGAGATCCCGCGCAT
CCGCATGCAGCTGCAGGAGTACGGCCAGGTCGGCGACGCCAACGTCTTCTACTGGTTCCA
GAACCGCAAGTCCCGCTCCAAGAACAAGCTGCGCTCCGGCGGGACAGGCCGCGCGGGGCT
CGGCCTCGGCGGCAACCGGGCCTCCGCGCCGGCGGCGGCGCACCGGGAGGCCGTGGCGCC
GTCGTTCACGCCGCCGCCACCAATCCTCCCGGCGCCCCAGCCGGTGCAGCCGCAGCAGCA
GCTTGTCTCGCCTGTGGCGGCGCCTACCTCGTCGTCGTCTTCCTCCTCCGACCGTTCGTC
CGGGTCCAGCAAGCCTGCGAGGGCTACGTCGACGCAGGCGATGTCCGTGACGACGGCCAT
GGACCTGCTCTCGCCGCTCGCCGCGGCGTGCCACCAGCAGATGCTCTATCAAGGCCAGCC
ACTGGAGTCGCCGCCGGCGCCTGCTCCCAAAGTGCACGGCATCGTGCCACACGACGAGCC
GGTCTTCCTGCAGTGGCCGCAGAGCCCCTGCCTGTCGGCCGTCGACCTCGGCGCCGCCAT
TCTTGGCGGCCAGTACATGCACCTGCCGGTGCCCGCTCCGCAGCCACCGTCGTCGCCGGG
CGCGGCGGGCATGTTCTGGGGGCTCTGCAACGACGTGCAAGCGCCAAACAACACCGGCCA
CAAGAGCTGCGCCTGGAGCGCCGGGCTCGGCCAGCACTGGTGCGGCTCCGCCGATCAGCT
CGGCCTCGGCAAGAGCAGCGCGGCGTCGATCGCCACCGTGTCTAGGCCGGAGGAGGCGCA
CGACGTCGACGCCACGAAGCACGGTCTGCTACAGTACGGCTTTGGCATCACCACGCCGCA
AGTGCACGTGGACGTTACCTCCTCGGCTGCTGGCGTTCTGCCTCCTGTTCCGTCCTCGCC
GTCGCCGCCGAACGCCGCCGTCACCGTCGCGAGCGTGGCCGCCACCGCTAGCCTGACTGA
TTTTGCTGCAAGTGCTATATCTGCTGGCGCCGTCGCTAACAATCAGTTTCAAGGTCTCGC
GGATTTCGGGCTCGTCGCCGGCGCCTGCTCCGGCGCCGGAGCCGCCGCCGCCGCCGCCGC
GCCCGAGGCGGGCAGTTCCGTGGCCGCGGTTGTGTGCGTCAGCGTCGCGGGCGCCGCGCC
GCCGCTCTTCTACCCGGCCGCGCACTTCAACGTGAGGCACTACGGCGACGAGGCCGAGCT
GCTCCGCTACAGAGGAGGCAGCCGCACGGAGCCTGTGCCCGTCGACGAGTCGGGCGTCAC
CGTCGAGCCGCTCCAGCAGGGCGCCGTCTACATTGTTGTCATGTAACTATAGATTAATTA
GTTATCTCTCCAATCCGTATCATTGCTGTACTAGTTAAGTGGCCCATATATATATATATA
TATGTGTTGCCAGTCTTGTCGATCAATTAATCAATCTAGCTTTTTTTTTTAGATAATAAT
TAATCAATCTAGCTAGGGTTCTAAT
>OS07G34880
ATGATGGCCTTAGGCGTGCCACCGCCTCCCTCGCGCGCCTACGTGTCCGGCCCGCTACGC
GACGATGACACTTTTGGCGGTGATCGTGTTCGGCGGCGGCGGCGGTGGCTCAAGGAGCAG
TGCCCTGCCATCATTGTCCACGGGGGTGGCAGGCGTGGAGGGGTCGGCCACAGGGCCCTG
GCTGCCGGAGTCTCTAAAATGCGTCTCCCAGCCCTAAACGCCGCCACCCACCGGATCCCC
TCCACCTCGCCCTTAAGTATCCCTCAGACCCTCACCATCACCCGCGATCCTCCCTACCCA
ATGCTGCCTCGAAGTCACGGCCACCGGACCGGCGGCGGCGGCTTCTCCCTCAAGTCCTCG
CCCTTCTCGTCAGTGGGCGAGGAGAGGGTTCCGGACCCGAAGCCGCGGCGGAACCCGCGG
CCGGAGCAGATCCGGATCCTGGAGGCCATCTTCAACTCCGGCATGGTCAACCCGCCGCGC
GACGAGATCCCGCGCATCCGCATGCAGCTGCAGGAGTACGGCCAGGTCGGCGACGCCAAC
GTCTTCTACTGGTTCCAGAACCGCAAGTCCCGCTCCAAGAACAAGCTGCGCTCCGGCGGG
ACAGGCCGCGCGGGGCTCGGGCTCGGCGGCAACCGGGCCTCAGAGCCGCCGGCGGCGGCG
ACGGCGCACCGGGAGGCCGTGGCACCGTCGTTCACGCCGCCACCGATCCTCCCGCCCCAG
CCGGTGCAGCCGCAGCAGCAGCTTGTCTCGCCGGTGGCGGCGCCCACCTCGTTGTCGTCA
TCGTCCTCCGACCGCTCGTCCGGGTCCAGCAAGCCCGCGAGGGCTACGTTGACGCAGGCG
ATGTCCGTGACGGCGGCCATGGACCTGCTCTCGCCGCTCCGCCGATCAGCTCGGCCACGG
CAAGAGCAGCGCCATGTCTAG
>OS05G48990
ATGGCCTCACCGAACAGGCACTGGCCGAGCATGTTCAGGTCCAATCTTGCCTGCAACATC
CAGCAGCAGCAGCAGCCTGACATGAACGGCAACGGCAGCTCGTCCTCTTCCTTCCTCCTC

TCGCCACCTACTGCTGCGACCACCGGCAACGGCAAGCCCTCCTTGCTCTCCTCAGGGTGT
GAGGAGGGGACGAGGAATCCGGAGCCGAAGCCGCGGTGGAACCCGAGGCCGGAGCAGATA
AGGATACTGGAGGGGATCTTCAACTCCGGGATGGTGAACCCGCCGCGCGACGAGATCCGC
CGCATCCGCCTGCAGCTGCAGGAGTACGGCCAGGTCGGCGACGCCAACGTCTTCTACTGG
TTCCAGAACCGCAAGTCCCGCACCAAGAACAAGCTGCGCGCCGCCGGCCACCACCACCAC
CACGGCCGCGCCGCCGCCCTGCCGCGCGCGTCGGCGCCGCCGTCGACGAACATCGTACTC
CCCTCTGCAGCGGCGGCGGCGCCCTTGACGCCGCCGCGGCGCCATCTCCTCGCCGCGACC
TCCTCCTCGTCCTCCTCCTCCGACCGCTCCTCCGGGTCCAGCAAGTCGGTGAAACCAGCT
GCTGCCGCGCTGCTGACGTCAGCCGCCATCGACCTTTTCTCGCCGGCGCCGGCGCCGACG
ACCCAGCTGCCCGCGTGCCAGCTCTACTACCATAGCCATCCCACGCCGCTGGCACGTGAT
GATCAGCTCATCACCTCGCCGGAGTCGTCGTCGCTCCTCCTGCAGTGGCCGGCGAGCCAG
TACATGCCGGCGACGGAGCTCGGCGGCGTCCTCGGCTCGTCGTCCCACACGCAAACCCCG
GCAGCGATCACCACCCACCCATCGACGATCTCACCCAGCGTGCTCCTCGGCCTATGCAAC
GAGGCACTAGGGCAGCATCAGCAAGAGACCATGGACGACATGATGATCACCTGCTCCAAC
CCCTCCAAGGTGTTCGACCACCATTCCATGGACGACATGAGCTGCACCGACGCGGTGAGC
GCCGTGAACAGGGACGACGAGAAGGCGAGGCTGGGGTTACTGCACTACGGCATCGGCGTC
ACTGCTGCTGCAAATCCGGCACCACATCATCATCATCATCATCATCATCTTGCCTCTCCT
GTGCATGATGCTGTCTCGGCTGCAGATGCTAGTACGGCGGCCATGATCCTTCCATTCACC
ACCACTGCTGCTGCGACGCCGAGCAACGTCGTCGCTACAAGCTCTGCACTCGCTGATCAG
TTGCAAGGGCTGTTGGATGCTGGGTTGCTGCAGGGAGGGGCGGCGCCGCCGCCGCCCTCG
GCGACGGTGGTGGCGGTGAGCCGCGACGACGAGACGATGTGCACCAAGACCACGAGCTAC
AGCTTCCCGGCGACGATGCACCTCAACGTGAAGATGTTCGGCGAGGCGGCCGTGCTGGTG
CGCTACAGCGGCGAGCCGGTGCTCGTCGACGACTCCGGCGTCACCGTCGAGCCGCTGCAG
CAGGGCGCGACCTACTACGTGCTGGTATCTGAGGAAGCTGTGCATTGAAGCCGTATTACC
TTGATTACATCCACAATTCGGCACTGATCATGCTTGCTGCATGCGATCGAATCATCGATC
ATCTAAGAGATTCTAAGTTAATTAGTAATTCGCTCACTAGTTTGGTAATAGCTAGCTAGC
AACCTTCATCGATCTGTTATTTCATTCAGTACGTACATGTCTACTTAAAATCACTGTCGT
GTAGTACTGTACGTGTTTTGCTCGTCTGATCAAACAGCAACATGCATGACGGTTTTAATT
TTGATGCAATAACCAGTGGAGTAAACCACATA
>OS03G20910
AAGCATGGTTTAATTGTTGTTGCCTCCTCTCAAGCTAGCTAGCTATACTACTCCGATCCT
CTCTCGCTTATTGAAACCTAGCTACTGCTTCACATGTAGCGCGTGCTGCAACTCGTGTTG
TGTCACTCACACACACATCCAAGAACCAAGATTCCGAGGTAACCAACGCTCGATCTTTTA
CTTACCCTATCTCCCGACGATCGATCGATCGATCGATGGAGGGGAGCAGTAATAGCCCGG
ATAGGCAGTCGTCGGGCGGCAGCCCGCCGGAGGAGCGCGGCGGCGGGGGAAGCGGTGGAG
GAGGAGGGCGGAGCGCCGCCGGCGAGCCGGTGCGGTCGCGGTGGACGCCCAAGCCGGAGC
AGATACTGATCCTGGAGTCCATCTTCAACAGCGGCATGGTCAACCCGCCCAAGGACGAGA
CCGTCCGCATCCGCAAGCTGCTCGAGAGGTTCGGCGCCGTCGGCGACGCCAACGTCTTCT
ACTGGTTCCAGAACCGCCGCTCGCGCTCGCGCCGCCGCCAGCGCCAGATGCAGGCGGCCG
CCGCCGCCGCCGCAGCGGCGGCCTCTTCCTCTTCCCCATCCGCCAACACCTCTCCCGCAG
CCGCGAGCGCCGCCACCGTGCAGGTGGGCCTCCCGCCCGGCGCCGTCGTCCACACCATGG
CCATGGGTGGGAGCGCGTGCCAGTACGAGCAGCAGGCGAGCTCGTCGTCGTCGTCCGGCA
GCACGGGAGGTTCGTCGCTGGGGCTGTTCGCGCACGGCGCGGGGGCCTCCGGCGCCGGCG
GGTACCTGCAGGCGTCGTGCGGCGCGTCCGCGTCGGCGTCGTCGGCGCTGGCGCCCGGGC
TGATGGGGGATGTGGTGGACAGCGGGGGAAGCGACGATCTCTTCGCCATCTCGAGGCAGA
TGGGGTTTGTGGGGAGCCCTCGCTGCTCGCCGGCCAGCTCGCCGGCGACGCCGAGCTCCG
CGGCCACCGCCGCGCAGCAGCAGTTCTACTCATGCCAATTACCTGCAGCGACGATCACGG
TGTTCATCAACGGAGTCCCAATGGAGATGCCGAGGGGTCCAATAGACTTGCGAGCCATGT
TCGGCCAGGATGTGATGCTCGTCCACTCTACTGGGGCCCTCCTCCCAGTCAACGACTATG
GAATCCTAATGCAGAGCCTCCAAATCGGAGAGAGCTACTTTCTGGTCGCTAGGCCACCTT
AAAAACGAGTTCTCGATCTATCCTTGCAGTAGATAGTCAGATATATATCTCTACTCGATA
CTCATTGGATCAGATGCATGAGCCGGCCGGGGTGCAGCTAGTTCTCAGTTAATCATCAAT
CAAGAATGCTGTCTTGGATTCAAGACCTTGGAGTATGTCTAAATCGATCAATCGTCTATC
GAGAACAAGACTATATATATTAGCTAGGTACGAGAAGCAAATTAATGCATGCATTGCAGC
ATATCGATCGGCAAAAGTACAACTACGTACTTATATATATGCCCCTTAATTAAAAACCTA
TGAAAGCGTGGGCGGTCGAGTTTGTTGTCCTTAGAATACAGATTACAATATGGTCGCGTC
TCTTCTCATCATTTTCACAATTCCATTACAATATGCATGTATGCATGCCTGCCCTTGGCA

GTCGAGCTATATATATTCTTTAATTTGTGCTAGCTATAGATGCACGTTTGTATTGTTTTT
TTTTACCGTGTGATGTAATGCTATATGTGTCAGCCAAACGCGCCTGGAATGTTGGTGCTT
GATTCAAGGCCGGTTGGAATTT
>OS08G14400
ATTAAGCTTGAATTGCTGCTCCCTTGGGCGCATGTGTGTCACCCTCTCCTTCCATTGATC
CTTTCACATCCTTTAATTTCTCTCTTTCTTCCACAACAAATCAAACTGTTCATCCTCCCC
TTCACCTTTGACCTTTCCAACCTAATTAATTAGCTACTACCCAATTAGCCGATCCATCCA
TGGACCGCACTGCGACTGCAAGCTGGGAGGTCATGAGCCGGCGAGGTGAGCAGCAGCAGC
AGCTGATGATGCAAGCTCCGGCCAGCCATAATGGTGGCAGCGGCGGCGGCGAGCCGGCGA
GGTCGCGGTGGGCGCCGAAGCCGGAGCAGATCCTGATACTGGAGTCCATCTTCAACAGCG
GCATGGTGAACCCGGCCAAGGACGAGACGGCGCGCATCCGCCGCCTCCTCGAGCGCTTCG
GCGCCGTCCGCGACGCCAACGTCTTCTACTGGTTCCAGAACCGCCGCTCCCGCTCCCGCC
GCCGCGCCCGCCAGCTCCAGCAAGCCTGCGGCGCCGCCCTCCATCAGCTCCCCTCCGCCG
CCGCCGCCGCCGGAGCCGGAGGAGGAGGAGATTACTATCACCACCACCACCAACCTTCGT
CTTCTCCTTTCCTCATGCACGGCGGTGGCGGCGGCGGCGTGGTCACTTCGACGACCGCGG
CGCCAGCGGTGGCGGCGTCCGGCCACTTCTTGGCGGACGAGGTCGACGGCGGCGGCGACG
ACGACCTCTTCGCCATCTCCCGGCAGATGGGCCTCATGGCTCGCCACGGCGGCGGCGACC
ACCATTACAGCAGCTACGCGGACAGCGACGCCACCCAGCTCAGCTACCAACCAACCGGGA
CGATCCAAGTGTTCATCAATGGCGTCGCATACGATGTGCCGAGCGGCGGAGCGCTGGACA
TGGCCGGCACGTTTGGCCGCGACGCCATGCTGGTGCACTCCTCCGGCGAGGTCCTTCCGG
TGGACGAGCACGGCGTGCTCATCAACAGCTTGCAGATGGGGGAGTGCTACTACCTGGTTT
CAAAATCGATCTGATTAATCGGATGGATGAAGGTGGAGAAGAAGATGCAAGATTTATGTA
TGCTCTTGTAAGCATGCGGGATTTGCTTCATTGATTAATTGGGAGATTGATTCCCAGCTC
CATGAATTGTTGTTAATTAGGTTGTCCAGGGATCATATCAGTGTGTACTAATGTGGTTGA
TTTCTCTCCATCGATGTATATTATTATCTCGATGCATCGTCCCCTTG
>OS07G48560
AAGCCAATTAAGCGAATTAAGCACACATCAATTGACCAAACCTATCTCTCTATCTCTCTC
GAGCTAGCGAGCTCTAGGTGTTCGACACCGAACAAGGCAGCTAGCTAGTGGCGATGGACG
GCGGCCACAGCCCGGACAGGCATGCGGCGGCGGCGGCGGGGGAGCCGGTGAGGTCGCGGT
GGACGCCGAAGCCGGAGCAGATACTCATCCTGGAGTCCATCTTCAACAGCGGCATGGTGA
ACCCGCCCAAGGACGAGACCGTCCGCATCCGCAAGCTGCTCGAGCGCTTCGGCGCCGTCG
GCGACGCCAACGTCTTCTACTGGTTCCAGAACCGCCGCTCGCGCTCCCGCCGCCGCCAGC
GCCAGCTGCAGGCGCAGGCGCAGGCGGCCGCGGCCGCCGCCTCGTCGGGATCTCCTCCGA
CTGCTTCGTCCGGTGGCCTCGCGCCTGGCCACGCCGGCTCGCCGGCTTCGTCGCTCGGGA
TGTTCGCGCACGGCGCCGCCGGGTACAGCTCCTCGTCGTCCTCATCGTGGCCGTCCTCGC
CGCCGTCGGTGGGGATGATGATGGGGGACGTGGACTACGGGGGCGGCGGCGACGACCTGT
TCGCCATCTCGAGGCAGATGGGGTACATGGACGGCGGCGGCGGCTCGTCGTCGTCGGCGG
CCGCCGGTCAGCATCAGCAGCAGCAGCTCTACTACTCGTGTCAACCTGCGACGATGACGG
TGTTCATCAACGGAGTGGCGACGGAGGTGCCAAGGGGACCGATCGATCTGAGATCAATGT
TTGGGCAGGACGTGATGCTGGTGCATTCAACGGGTGCTCTTCTTCCAGCCAACGAGTACG
GCATCCTCCTCCATTCTCTCCAGATGGGCGAGAGCTACTTCCTGGTCACGAGGTCGTCTT
GATCGAGTTGAGCGATTCGTCGATTGATTGATCGATGGCAACGTACGTATTGTGTCAGCA
AGATTTAGGTACCCCTTGTCCGTTGGATCAGATCGATATGCAAGCAACGCATGCTTTGCA
GGTGCCGGCCGGTTAATTACGCGATCGATCGAGCTAGCAGATGATGAATGCTGCACGTAC
GCATGGCAATTGTCACTGCGACGAGCCTGCATATATATGAACGATCCATTGATCGTCATT
CTAGCGACCAAGCAGCACGATAGTACAAGTATATACTATACAATGCAATTGCAGCGACGA
ACTAGTTAATTATAATACAGATAATGCATGTGTACTTCTGTGGCCATACATATATATATA
GAGAGAGAGGGGATTAATTACTATATTCGTTTTTCATCTAGCTGGTCGATCTCTGTGTGT
ACTTGTATGTATCCCGTTCTCGATCTTTATATCAGAATATTTTATATATGTATATTTTAC
AATTATATATATAGCCGTCTCGATCGCCAAGCAAA

>PpaWOX02_1
ATAGTAACCCCACGCTTTCCTTCGTCGACGTTCATCCACAGACCTGTCTGTGGCTGCGGC
AGAAGACGCTGGGCTCGTGGTTCTCGCTATCCGAGGCACAAGGACCAGCCGCTTGTTCAA
AACTTCTCCCCCTGGCACCACTCTGCCATCAATCCGTAAACCCTAAAGGAGTCCGGTCGG
TCCACGCATGGCTTCTGAGACGTGAGCGTAATCCATGTCGTCTTCCTGCGCCCGTGGGCG
TGTAGTTTGTTGAGGTGTTTGTTACCACAGCTTTCTCGGCGTTCCTTTTTGTTTTTCTTA

TAGTTTTTAATAGCCTTTCCTGGATTGCAGGGAGGGTTTCTTGGAGTATTCTTGTAGGTC
TGGACGTTTGCTTGGGTTGGACCGGTGTTGAGATCGTTTGACAATTTACGGCCTTTTGAA
GGGTCCTCTGCGGGAGATTGTAGTTTGCAGCGCAGTTCAAGTGAAGGTAATGGAGGCTAC
GCTATTTGGTGGTGGATGACAAAGTCAGTTCCCCTGACTTCATTAATCCATGGTTATGCG
ATTCTCAGGACTGATCTCGATACCTTGGAGCCGTTGCAAGGGATACATTGGAAATCAAGT
CGATTGCATCTTGGTGGTAGGGTGTTTTCACTGAGAGTTTGCAGTGGGTGCTATAGCCCG
ATGCCGTTTGAGGAAAGAGACAGTATCTCCACAATAACGAAGACTTTCTCTCTGGGATTT
TCCAGCTTTGTAGAACTGCAGGGCTAAATTTGCGCAGTCATTTCACCGTTAGCTGAAGGG
CGGTGTGTTGTTCAAAGCTCTCTTCTTAGTTTTGATATCTGACGGTATGTGAAAGTTCAC
AGGATCGAAAACAGGCAGAGCAACGGCATGGAATCTGAATCTAGGTTAGGCCGAATGATG
GACATGACACCTTTGGGGTCGGGATTGCAAGGGCAACCTGTTCCTGGTGGAGCTGCGCTC
GGCCTTGGGCCTTCGTTGGAGAATTCGTTGCCGCAACCCATGTACACTCGGGGGTCTGGG
CAGGTAATGACAGAAGAGCAGCTCGAAACATTGCGACGACAGACTTCGGTGTATGCAACA
ATCTGTCAACAACTTGTTGAAATGCACAAAGCGAGTGTTTCACAACAAGCATCTCTTCCT
GGCATTCTAGCAAGTGGTCAGATTGTGTCGATGGACCATCTCACTGGAACACCCCCTCAC
AAATCGACAGCAAGACAGCGGTGGACCCCCAGCCAACATCAGCTGCAAATTTTAGAAAAG
TTGTTTGAGCAAGGCAGTGGCACACCCAACAAACAGCGCATTAAAGAGATTACTGCCGAA
CTCAGTCAGCATGGTGCAATCTCGGAGACAAATGTGTACAACTGGTTTCAGAATCGCAAA
GCCCGAGCCAAAAGGAAGCAGCAATTGGTTACCCCAAGGGATGGTGAATCGGAAGCAGAT
ACAGATGTAGAGTCACCAAAGGAAAAACGTACAAGACAGGAAGGTGAACAAAATCAGGAC
GAATCAGGGGGTGTTGGTGATACAAATGGTGGAGGCAACTCTGATGGAGCTGGAAATGGG
GTTCCTGAGCAAAGAGCTGCCAACTTTGACCAGCAGGATGCCGCTTCGTCTGCGCTGCTG
CATTCACAAACAGATACTAAACCTGATATATCATCATTTAACAGGAGTGCTGGGTTCGAT
CCTCATAATGTATCTCAAGGCATCCCTCCCATGATGAGTTAAGCGGCATGCAGCTGGTCT
TGTAATCTTGGTATTTACGGTTGGCAAGAGACTTCATTAGTTTGAGAATCAACAAGACAT
GGAGTCTGTGTAGTTATGATGTAAGGGGATAGATAGAAGTGTGTTTCGCTTTTGCATTGC
CTCCGATGTGGTGTGCCCTCAAGGGGATCGAGCCACCCGCGGATAAAGCAGTAATTTTCC
CATTGCAGCAATTGCGCTGCTCTCTTGGGTGTGATAGACAGCTGGTAGTGATTCATGCGT
TCATCGTGTGGTTTTTAAACATGTTGAACTATTGAATACACACATGATTGCTCATGAAGG
ATCGCCGTAGGGATTCCCGAGATCTTCTGTATGCTTCCCTGAGCTATACATGGTCTTCCT
TGACAATCTCAAACTTAATCTAATTACTTTGTCAATCTCAATAGGCTTTGTGGTAATAAC
ATTCTTATTACCAATGGTATAAAAGTAAGATAGTCATCGAATTGAAGGTTTATTCGTGTA
CTGTTTTTTTCGTTTTTTAAATAAAATAAAACTAT
>PpaWOX02_2
ATAGTAACCCCACGCTTTCCTTCGTCGACGTTCATCCACAGACCTGTCTGTGGCTGCGGC
AGAAGACGCTGGGCTCGTGGTTCTCGCTATCCGAGGCACAAGGACCAGCCGCTTGTTCAA
AACTTCTCCCCCTGGCACCACTCTGCCATCAATCCGTAAACCCTAAAGGAGTCCGGTCGG
TCCACGCATGGCTTCTGAGACGTGAGCGTAATCCATGTCGTCTTCCTGCGCCCGTGGGCG
TGTAGTTTGTTGAGGTGTTTGTTACCACAGCTTTCTCGGCGTTCCTTTTTGTTTTTCTTA
TAGTTTTTAATAGCCTTTCCTGGATTGCAGGGAGGGTTTCTTGGAGTATTCTTGTAGGTC
TGGACGTTTGCTTGGGTTGGACCGGTGTTGAGATCGTTTGACAATTTACGGCCTTTTGAA
GGGTCCTCTGCGGGAGATTGTAGTTTGCAGCGCAGTTCAAGGACTGATCTCGATACCTTG
GAGCCGTTGCAAGGGATACATTGGAAATCAAGTCGATTGCATCTTGGTGGTAGGGTGTTT
TCACTGAGAGTTTGCAGTGGGTGCTATAGCCCGATGCCGTTTGAGGAAAGAGACAGTATC
TCCACAATAACGAAGACTTTCTCTCTGGGATTTTCCAGCTTTGTAGAACTGCAGGGCTAA
ATTTGCGCAGTCATTTCACCGTTAGCTGAAGGGCGGTGTGTTGTTCAAAGCTCTCTTCTT
AGTTTTGATATCTGACGGTATGTGAAAGTTCACAGGATCGAAAACAGGCAGAGCAACGGC
ATGGAATCTGAATCTAGGTTAGGCCGAATGATGGACATGACACCTTTGGGGTCGGGATTG
CAAGGGCAACCTGTTCCTGGTGGAGCTGCGCTCGGCCTTGGGCCTTCGTTGGAGAATTCG
TTGCCGCAACCCATGTACACTCGGGGGTCTGGGCAGGTAATGACAGAAGAGCAGCTCGAA
ACATTGCGACGACAGACTTCGGTGTATGCAACAATCTGTCAACAACTTGTTGAAATGCAC
AAAGCGAGTGTTTCACAACAAGCATCTCTTCCTGGCATTCTAGCAAGTGGTCAGATTGTG
TCGATGGACCATCTCACTGGAACACCCCCTCACAAATCGACAGCAAGACAGCGGTGGACC
CCCAGCCAACATCAGCTGCAAATTTTAGAAAAGTTGTTTGAGCAAGGCAGTGGCACACCC
AACAAACAGCGCATTAAAGAGATTACTGCCGAACTCAGTCAGCATGGTGCAATCTCGGAG
ACAAATGTGTACAACTGGTTTCAGAATCGCAAAGCCCGAGCCAAAAGGAAGCAGCAATTG
GTTACCCCAAGGGATGGTGAATCGGAAGCAGATACAGATGTAGAGTCACCAAAGGAAAAA

CGTACAAGACAGGAAGGTGAACAAAATCAGGACGAATCAGGGGGTGTTGGTGATACAAAT
GGTGGAGGCAACTCTGATGGAGCTGGAAATGGGGTTCCTGAGCAAAGAGCTGCCAACTTT
GACCAGCAGGATGCCGCTTCGTCTGCGCTGCTGCATTCACAAACAGATACTAAACCTGAT
ATATCATCATTTAACAGGAGTGCTGGGTTCGATCCTCATAATGTATCTCAAGGCATCCCT
CCCATGATGAGTTAAGCGGCATGCAGCTGGTCTTGTAATCTTGGTATTTACGGTTGGCAA
GAGACTTCATTAGTTTGAGAATCAACAAGACATGGAGTCTGTGTAGTTATGATGTAAGGG
GATAGATAGAAGTGTGTTTCGCTTTTGCATTGCCTCCGATGTGGTGTGCCCTCAAGGGGA
TCGAGCCACCCGCGGATAAAGCAGTAATTTTCCCATTGCAGCAATTGCGCTGCTCTCTTG
GGTGTGATAGACAGCTGGTAGTGATTCATGCGTTCATCGTGTGGTTTTTAAACATGTTGA
ACTATTGAATACACACATGATTGCTCATGAAGGATCGCCGTAGGGATTCCCGAGATCTTC
TGTATGCTTCCCTGAGCTATACATGGTCTTCCTTGACAATCTCAAACTTAATCTAATTAC
TTTGTCAATCTCAATAGGCTTTGTGGTAATAACATTCTTATTACCAATGGTATAAAAGTA
AGATAGTCATCGAATTGAAGGTTTATTCGTGTACTGTTTTTTTCGTTTTTTAAATAAAAT
AAAACTAT
>PpaWOX02_3
ATAGTAACCCCACGCTTTCCTTCGTCGACGTTCATCCACAGACCTGTCTGTGGCTGCGGC
AGAAGACGCTGGGCTCGTGGTTCTCGCTATCCGAGGCACAAGGACCAGCCGCTTGTTCAA
AACTTCTCCCCCTGGCACCACTCTGCCATCAATCCGTAAACCCTAAAGGAGTCCGGTCGG
TCCACGCATGGCTTCTGAGACGGAGGGTTTCTTGGAGTATTCTTGTAGGTCTGGACGTTT
GCTTGGGTTGGACCGGTGTTGAGATCGTTTGACAATTTACGGCCTTTTGAAGGGTCCTCT
GCGGGAGATTGTAGTTTGCAGCGCAGTTCAAGTGAAGGTAATGGAGGCTACGCTATTTGG
TGGTGGATGACAAAGTCAGTTCCCCTGACTTCATTAATCCATGGTTATGCGATTCTCAGG
ACTGATCTCGATACCTTGGAGCCGTTGCAAGGGATACATTGGAAATCAAGTCGATTGCAT
CTTGGTGGATCGAAAACAGGCAGAGCAACGGCATGGAATCTGAATCTAGGTTAGGCCGAA
TGATGGACATGACACCTTTGGGGTCGGGATTGCAAGGGCAACCTGTTCCTGGTGGAGCTG
CGCTCGGCCTTGGGCCTTCGTTGGAGAATTCGTTGCCGCAACCCATGTACACTCGGGGGT
CTGGGCAGGTAATGACAGAAGAGCAGCTCGAAACATTGCGACGACAGACTTCGGTGTATG
CAACAATCTGTCAACAACTTGTTGAAATGCACAAAGCGAGTGTTTCACAACAAGCATCTC
TTCCTGGCATTCTAGCAAGTGGTCAGATTGTGTCGATGGACCATCTCACTGGAACACCCC
CTCACAAATCGACAGCAAGACAGCGGTGGACCCCCAGCCAACATCAGCTGCAAATTTTAG
AAAAGTTGTTTGAGCAAGGCAGTGGCACACCCAACAAACAGCGCATTAAAGAGATTACTG
CCGAACTCAGTCAGCATGGTGCAATCTCGGAGACAAATGTGTACAACTGGTTTCAGAATC
GCAAAGCCCGAGCCAAAAGGAAGCAGCAATTGGTTACCCCAAGGGATGGTGAATCGGAAG
CAGATACAGATGTAGAGTCACCAAAGGAAAAACGTACAAGACAGGAAGGTGAACAAAATC
AGGACGAATCAGGGGGTGTTGGTGATACAAATGGTGGAGGCAACTCTGATGGAGCTGGAA
ATGGGGTTCCTGAGCAAAGAGCTGCCAACTTTGACCAGCAGGATGCCGCTTCGTCTGCGC
TGCTGCATTCACAAACAGATACTAAACCTGATATATCATCATTTAACAGGAGTGCTGGGT
TCGATCCTCATAATGTATCTCAAGGCATCCCTCCCATGATGAGTTAAGCGGCATGCAGCT
GGTCTTGTAATCTTGGTATTTACGGTTGGCAAGAGACTTCATTAGTTTGAGAATCAACAA
GACATGGAGTCTGTGTAGTTATGATGTAAGGGGATAGATAGAAGTGTGTTTCGCTTTTGC
ATTGCCTCCGATGTGGTGTGCCCTCAAGGGGATCGAGCCACCCGCGGATAAAGCAGTAAT
TTTCCCATTGCAGCAATTGCGCTGCTCTCTTGGGTGTGATAGACAGCTGGTAGTGATTCA
TGCGTTCATCGTGTGGTTTTTAAACATGTTGAACTATTGAATACACACATGATTGCTCAT
GAAGGATCGCCGTAGGGATTCCCGAGATCTTCTGTATGCTTCCCTGAGCTATACATGGTC
TTCCTTGACAATCTCAAACTTAATCTAATTACTTTGTCAATCTCAATAGGCTTTGTGGTA
ATAACATTCTTATTACCAATGGTATAAAAGTAAGATAGTCATCGAATTGAAGGTTTATTC
GTGTACTGTTTTTTTCGTTTTTTAAATAAAATAAAACTAT
>PpaWOX02_4
ATAGTAACCCCACGCTTTCCTTCGTCGACGTTCATCCACAGACCTGTCTGTGGCTGCGGC
AGAAGACGCTGGGCTCGTGGTTCTCGCTATCCGAGGCACAAGGACCAGCCGCTTGTTCAA
AACTTCTCCCCCTGGCACCACTCTGCCATCAATCCGTAAACCCTAAAGGAGTCCGGTCGG
TCCACGCATGGCTTCTGAGACGGAGGGTTTCTTGGAGTATTCTTGTAGGTCTGGACGTTT
GCTTGGGTTGGACCGGTGTTGAGATCGTTTGACAATTTACGGCCTTTTGAAGGGTCCTCT
GCGGGAGATTGTAGTTTGCAGCGCAGTTCAAGGACTGATCTCGATACCTTGGAGCCGTTG
CAAGGGATACATTGGAAATCAAGTCGATTGCATCTTGGTGGTAGGGTGTTTTCACTGAGA
GTTTGCAGTGGGTGCTATAGCCCGATGCCGTTTGAGGAAAGAGACAGTATCTCCACAATA
ACGAAGACTTTCTCTCTGGGATTTTCCAGCTTTGTAGAACTGCAGGGCTAAATTTGCGCA

GTCATTTCACCGTTAGCTGAAGGGCGGTGTGTTGTTCAAAGCTCTCTTCTTAGTTTTGAT
ATCTGACGGTATGTGAAAGTTCACAGGATCGAAAACAGGCAGAGCAACGGCATGGAATCT
GAATCTAGGTTAGGCCGAATGATGGACATGACACCTTTGGGGTCGGGATTGCAAGGGCAA
CCTGTTCCTGGTGGAGCTGCGCTCGGCCTTGGGCCTTCGTTGGAGAATTCGTTGCCGCAA
CCCATGTACACTCGGGGGTCTGGGCAGGTAATGACAGAAGAGCAGCTCGAAACATTGCGA
CGACAGACTTCGGTGTATGCAACAATCTGTCAACAACTTGTTGAAATGCACAAAGCGAGT
GTTTCACAACAAGCATCTCTTCCTGGCATTCTAGCAAGTGGTCAGATTGTGTCGATGGAC
CATCTCACTGGAACACCCCCTCACAAATCGACAGCAAGACAGCGGTGGACCCCCAGCCAA
CATCAGCTGCAAATTTTAGAAAAGTTGTTTGAGCAAGGCAGTGGCACACCCAACAAACAG
CGCATTAAAGAGATTACTGCCGAACTCAGTCAGCATGGTGCAATCTCGGAGACAAATGTG
TACAACTGGTTTCAGAATCGCAAAGCCCGAGCCAAAAGGAAGCAGCAATTGGTTACCCCA
AGGGATGGTGAATCGGAAGCAGATACAGATGTAGAGTCACCAAAGGAAAAACGTACAAGA
CAGGAAGGTGAACAAAATCAGGACGAATCAGGGGGTGTTGGTGATACAAATGGTGGAGGC
AACTCTGATGGAGCTGGAAATGGGGTTCCTGAGCAAAGAGCTGCCAACTTTGACCAGCAG
GATGCCGCTTCGTCTGCGCTGCTGCATTCACAAACAGATACTAAACCTGATATATCATCA
TTTAACAGGAGTGCTGGGTTCGATCCTCATAATGTATCTCAAGGCATCCCTCCCATGATG
AGTTAAGCGGCATGCAGCTGGTCTTGTAATCTTGGTATTTACGGTTGGCAAGAGACTTCA
TTAGTTTGAGAATCAACAAGACATGGAGTCTGTGTAGTTATGATGTAAGGGGATAGATAG
AAGTGTGTTTCGCTTTTGCATTGCCTCCGATGTGGTGTGCCCTCAAGGGGATCGAGCCAC
CCGCGGATAAAGCAGTAATTTTCCCATTGCAGCAATTGCGCTGCTCTCTTGGGTGTGATA
GACAGCTGGTAGTGATTCATGCGTTCATCGTGTGGTTTTTAAACATGTTGAACTATTGAA
TACACACATGATTGCTCATGAAGGATCGCCGTAGGGATTCCCGAGATCTTCTGTATGCTT
CCCTGAGCTATACATGGTCTTCCTTGACAATCTCAAACTTAATCTAATTACTTTGTCAAT
CTCAATAGGCTTTGTGGTAATAACATTCTTATTACCAATGGTATAAAAGTAAGATAGTCA
TCGAATTGAAGGTTTATTCGTGTACTGTTTTTTTCGTTTTTTAAATAAAATAAAACTAT
>PpaWOX02_5
ATAGTAACCCCACGCTTTCCTTCGTCGACGTTCATCCACAGACCTGTCTGTGGCTGCGGC
AGAAGACGCTGGGCTCGTGGTTCTCGCTATCCGAGGCACAAGGACCAGCCGCTTGTTCAA
AACTTCTCCCCCTGGCACCACTCTGCCATCAATCCGTAAACCCTAAAGGAGTCCGGTCGG
TCCACGCATGGCTTCTGAGACGGAGGGTTTCTTGGAGTATTCTTGTAGGTCTGGACGTTT
GCTTGGGTTGGACCGGTGTTGAGATCGTTTGACAATTTACGGCCTTTTGAAGGGTCCTCT
GCGGGAGATTGTAGTTTGCAGCGCAGTTCAAGGACTGATCTCGATACCTTGGAGCCGTTG
CAAGGGATACATTGGAAATCAAGTCGATTGCATCTTGGTGGCAGAGCAACGGCATGGAAT
CTGAATCTAGGTTAGGCCGAATGATGGACATGACACCTTTGGGGTCGGGATTGCAAGGGC
AACCTGTTCCTGGTGGAGCTGCGCTCGGCCTTGGGCCTTCGTTGGAGAATTCGTTGCCGC
AACCCATGTACACTCGGGGGTCTGGGCAGGTAATGACAGAAGAGCAGCTCGAAACATTGC
GACGACAGACTTCGGTGTATGCAACAATCTGTCAACAACTTGTTGAAATGCACAAAGCGA
GTGTTTCACAACAAGCATCTCTTCCTGGCATTCTAGCAAGTGGTCAGATTGTGTCGATGG
ACCATCTCACTGGAACACCCCCTCACAAATCGACAGCAAGACAGCGGTGGACCCCCAGCC
AACATCAGCTGCAAATTTTAGAAAAGTTGTTTGAGCAAGGCAGTGGCACACCCAACAAAC
AGCGCATTAAAGAGATTACTGCCGAACTCAGTCAGCATGGTGCAATCTCGGAGACAAATG
TGTACAACTGGTTTCAGAATCGCAAAGCCCGAGCCAAAAGGAAGCAGCAATTGGTTACCC
CAAGGGATGGTGAATCGGAAGCAGATACAGATGTAGAGTCACCAAAGGAAAAACGTACAA
GACAGGAAGGTGAACAAAATCAGGACGAATCAGGGGGTGTTGGTGATACAAATGGTGGAG
GCAACTCTGATGGAGCTGGAAATGGGGTTCCTGAGCAAAGAGCTGCCAACTTTGACCAGC
AGGATGCCGCTTCGTCTGCGCTGCTGCATTCACAAACAGATACTAAACCTGATATATCAT
CATTTAACAGGAGTGCTGGGTTCGATCCTCATAATGTATCTCAAGGCATCCCTCCCATGA
TGAGTTAAGCGGCATGCAGCTGGTCTTGTAATCTTGGTATTTACGGTTGGCAAGAGACTT
CATTAGTTTGAGAATCAACAAGACATGGAGTCTGTGTAGTTATGATGTAAGGGGATAGAT
AGAAGTGTGTTTCGCTTTTGCATTGCCTCCGATGTGGTGTGCCCTCAAGGGGATCGAGCC
ACCCGCGGATAAAGCAGTAATTTTCCCATTGCAGCAATTGCGCTGCTCTCTTGGGTGTGA
TAGACAGCTGGTAGTGATTCATGCGTTCATCGTGTGGTTTTTAAACATGTTGAACTATTG
AATACACACATGATTGCTCATGAAGGATCGCCGTAGGGATTCCCGAGATCTTCTGTATGC
TTCCCTGAGCTATACATGGTCTTCCTTGACAATCTCAAACTTAATCTAATTACTTTGTCA
ATCTCAATAGGCTTTGTGGTAATAACATTCTTATTACCAATGGTATAAAAGTAAGATAGT
CATCGAATTGAAGGTTTATTCGTGTACTGTTTTTTTCGTTTTTTAAATAAAATAAAACTA
T

>PpaWOX01
GGAAGCTTTACCATCTATCTGGCAGAGAGAGAGAGTCCTAAGGAACCTTCGCCGATCGAC
GCTCTCCTCCCGGGACGTTGTGATGTAGGAGGATTCCTCACTCTGCTCTTCGTCTCGGGC
TCGTGGTTTCGTTTACGGATTGATCTCGTTAAATTGGACTGCACAAGACAGGCTAGAATC
CAAGTCGGTTGCATTTTGGCGGTAGGGTGTTTGTATCAAGAGTTTGCGATGGTTCCAGCA
GCGCGACACCATTTGGGGAAAGGACCAGTTTCTCCTCAATAACGAGGAACATCTGCAAGA
CTAAACTTGTGCAATCATCTCACCCTTTCTGAAGGGCGGTGTGTCGTTCAAAGCTCTCTT
CTCAATTTTGACATTTGACCCCATGTGAAAGTTCACAGGGTAAAACACATGCAGAGCAAC
GGGATGGAACCTGAATCTAGGTTAGGTCGAATGATGGATATGTCTCCTATAGGGTCGGGA
TTACGAGGGCAGCCTGTTTCTGGTGGAGCAATGCTTGGTGTCGGGCCTTCCTTGGAGAAT
CCGTTGCCGCAACCCATGTATACTCGGGGATCTGGGCAGGTGATGACAGAGGAGCAGCTC
GAAACATTACGACGACAGATTTCAGTGTATGCAACGATCTGTCAGCAGCTTGTTGAAATG
CACAAAGCGAGTGTTTCCCAGCAAGCATCCCTTCCGGGTATTTTAGCAGGTGGTCAGATC
GTATCTATGGATCAGATGACTGGAACACCTACTCATAAGTCGACAGCAAGGCAGCGATGG
ACCCCTAGTCAGCATCAGCTACAAATACTGGAAAAGTTGTTTGAGCAAGGCAGTGGTACG
CCCAACAAAGTGCGCATCAAAGAGATTACTGCTGAGCTCAGTCAGCATGGTGCTATCTCG
GAGACCAACGTGTACAACTGGTTCCAAAACCGGAAGGCTCGAGCCAAAAGAAAACAGCAA
CTGGTTACCCCGAAGGATGGTGAATCTGAAGCTGATTCAGATGGGGAGTCGCCGAAGGAA
AAACGTACAAGACAGAAAGGTGATCAAAACCAAGATGAATCAGTAGGTATCGGAGATCCA
CATGGTGGAGGAAGTTTTGATGGAGCCGGTAATGGGGTCCCTGAGCAAAAAGCTGTTAAT
TCTGACCAACAAGATGCCACATCATCTGCACTGCTGCATCCACAAGCAGATACTAAACCT
GATATATCATCATTTAACAGGGGTGCTGGGTTTGATCCTTATAATATGCCTCAAGGCATT
CCTCCCATGATGAGTTGAGTGGCATGCTGCTGGTCTTGTAACTTCGGTATTAATGGTTGG
CAAGGGACTACATAAGTCTGAGAATCTACAAGACATGGAGTCTGGCTAGAAGTGTGTTTC
GCTTTGCATGGCCTCTGATGTGGTGTGCTCTCAAGGGCATTGAGCCACCCGCGGATAAAG
CAGTAGACTTTCCATTGCAGCAGTTGTGCTGCTCCCTTGGGTGTATTAGATAGTAGTTAT
TTATGAATTCATCGTGTGGTTTTGAAGCACATCGGTCTATGGAATACACACGT
>PpaWOX03
ATGGCCTGTGAACAAAGGGCGTCAACCAATCCCCCTACCCGATCACGATGGAGTGCTAAT
CAGCAGCAGCTGCAAAACCTAGAGAGTATCTTCGAGCAGGGAAATGGAAACACTCCCAAC
AAAGCTAGGATTAAAGACATAACCATAGAACTTAACCAGTTTGGTCACATCTCAGAAACA
AATGTGTACAACTGGTTTCAGAACCGTAAAGCACGAGCTAAGCGAAAGCTGCAGCAGCGG
TGGCGGGTGAACCAGATGAGGAGCGGGGAAGCGGAGATCAGTGGGGATGTGCAGCCGTAT
CTGCCGGACGCCAAAAGGTTGAAGGCGGTCGGGCCTCAGAGCCAGTCGGCGACCGGGTCA
GCAGCCACGCCTGCGTCGGCGCTGGACGCAGGGAGCTCCATCGCTGCGGCGGAGATTGAC
ACTGAGCTGCGGTCGCGGCCGGCATTGATGCCGCTGACGTCGATTACGCCGCTGACGATG
CCGCTGACGGTGACGCGCGTCGAAGCGATAGAAGAGACGCTAGGCGGCGGGGCAGAGCAT
TGGCATGATCAAGGTCTGCCCGTCCTCCTGCCCCCTTCTTTACATGGAGAGACCTCGTTT
ATTGATTATTACCGCTTCAGAAAATAA

>OT13G01350
ATGGGGTCGAGCGACGCGCGGACGGACGACGCGAACGGGCTCGAGAGTGGGAAGAGTGCG
GTGGAGCGGCGCGCGGCGATGGTGCACGCGGCGAAGGCGACGCTGCCGGGAGACGTGCGC
GCGCCGCCGCCGGGGGCGGCGGTGCACATGACGGAACGGCAGTTGTACGCGTTTCGACAG
CAGATCGCGGCGTACGCGCACATCTGTCAGCAGTTGTTGCAGATAACGACGGTGAGTGCG
ACGCAGCAGACGCCGAGACAGCGAGAGATGCGGCACGAGGTTCCGGGGGCGACTGGGGCG
AACTGGGGGGCGAGCGTTCCGGTGACGGCGCCGGCGTCCGCGCCGCTCTCGAGTTCGAAT
CACTCGAGTGGGAAGACGCGAGGCGAGGATAAGCAGGCGAGAGGACCGAGGTGGACGGGG
ACGCCGACGCAGTACGAGATTTTGGAGGATCTTTTCCAGAAGGGTGAGCAACCGCCGGTT
CGGGACAGGCTGACCGAGCTCACGGAGATGCTCAAGCAACACGGTCCGGTTCAAGAGTCC
AACGTGTACAATTGGTTCCAAAATAGAAGGAGTCGCGAAAAGAAGTTACAAGCCATGGCC
ATGGGGCACTAG

>Smo404134
ATGGATTCCCTGCCAACGGCATCTTGTTCTACTCCAAGTGTAGGAAGACGTTCGTACGGA
AGACGGTGGATTGCAAACGAAGTGACGTGGCTCGAGTTGAATTGCCACCTTCTAAAAGGC
CGCAGCGAAGTGTTTATTTGTGGCGTATTTACTGGCTCGCCCCAAAGATCCTTCTCTCTG

TTGTTTTCACCCCCTGCATCTCTAGATAGAATGCCGAGGCGCAAGCAGCAGGCATCGCGG
GCATCGTCCGTGTCTGATCAGATCGATCAGGAGCGCAGGGAGATGCGCCTGGAGATTAAG
GCATTGCATGTGCATCACAGAAGTCAAAGGAATAACACCAATATCTCTTCGGAGCCAGCG
CCGCCGAGGCCCGTAGGCGTGCAGCAACGATGGGAGCCAAACAGCTACCAGCTGCAAATC
TTGGAGGAGTTCTACGCCAAAGCGACGCCGCCATCGCCGGAGAACATCGCCAATATAGCC
GAGCTCGTCGGCCAGGTCGACCATTCCAAAGTGTACTACTGGTTCAGTAACAAGAAGTCC
CGCGAGAAACGCAAGCGCCGCCGCCTTGAGGAGGCCGCCGCATCCGCCTCTTCCTCTCCT
GCGTTTTCTGCCATCGCCACCACGGCCGCTGCTTCATCCGGGTCGGAGCAATCGCACCAT
CAGTCGTACGCTGACCAGATTGTGAATGACGGTAGTTTTCTCCCAGATGCGTGCCTGTTT
GAAACCAACGTGACGCTGGAGCAGCCCCGAATCATTCCGTCGCACGAAGAAGAATCACCA
TTGCAGTTGAGTCTCGGTTGGAGCTTTGCCAACCTGGACGAGCACTGGGAATTCTCTGTT
TTGATAGATCGGACTCTAAGACTGTCGCCGTTAGGGAAATGGAAACTGGACGATTATCGA
AACGTCATTCTAAGTAGCACATTCTGGCCGAAGATCTAG
>Smo407131
ATGCCGAGGAGCAAGCAGCAGGCATCGCGGGCATCGTCCGTGTCTGATCAGATCGATCAG
GAGCGCAGGGAGATGCGCCTGGAGATTAAGGCCTTGCATGTGCATCACAGAAGTCAAAGG
AATAACATCAATATCTCTTCGGAGCCAGCGCCGCCGAGGCCCGTAGGCGTGCAGCAACGA
TGGGAGCCAAACAGCTACCAGCTGCAAATCTTGGAGGAGTTCTACGCCAAAGCGACGCCA
CCATCGCCGGAGAACATCGCCAATATAGCCGAGCTCGTCGGCCAGGTCGACCATTCCAAA
GTGTACTACTGGTTCAGTAACAAGAAGTCCCGCGAGAAACGCAAGCGCCGCCGCCTTGAG
GAGGCCGGCGCATCCGCCTCTTCCTCTCCTGCGTTTTCCGCCATCGCCACCACCGCCGCT
GCTTCATCCGGGTCGGAGCAATCGCACCATCAGTCGTACGCTGACCAGATCGTGAATGAC
GGTAGTTTTCTCCCAGATGCGTGCCTGTTTGAAACCAACGTGACGCTGGAGCAGCCACGA
ATCGTGATCCGGCTCTGGCCATGGAGTTCGTCTTCAATTGTGGCGCTGGCGGTCGATCTT
GACATCGATAACATGCAGCAGGCTTCCAAGGCGGCGATTATATTTGCTACTGATGATTTG
CATGTCTTTGCTTGCACTGTAGGGAAATGGAAACTAGACGATTATCGAAACGTCCTTCTA
AGTAGCACATTCTGGCCGAAGATCTAG
>Smo417553
ATGAGGCGGTGCGAGCAGCAGGCGGCATCATCGCCGGCGTCGTCCGAGTCTGATCAGATC
GATCAGGAGCGGGATATCAACACAGAGCTCCTGGAGCTAAAGGCATTGCATATGCATCAC
AGAAGTCGCAGGAAACACATCGAAATCTCATCCGTGCCAGCGCCGCCAGCGCCGACAAGG
CCTGCGCAGCAGCGGTGGCGGCCAAACAGTCAGCAGCTGGGCATCCTGGAGGAATTCTAC
GCGAAAGGGACGCCGCCATCGCAAGAAAACGTCACGGAGATAGCCGAGCTCATCGGCCAC
CACGGCCCGGTGGACGAATCCAAAGTGTACTATTGGTTTCAGAACAAGAAGTCCCGCGAG
AAACGCAAGCGGCGCCGCATCGAGGAGGCCAACGCCGCCTCTGGCGCCTCCGCCTCTTCT
TCTCCCGCCGCCGCCAGTGCCACCGCCGCTTTCCAGCAGGATTTGTCCTCCTCCGCCCCT
AGCGCTTCCGCTTCAGCTTCATCCGGGTCGGAGCAATTGCACCATCCCGCCGCGCAGTCG
TATGCTGCTGCCCAGATCGTGATTGACGGGGGCGTGGTTTCTATCATCGACGACAAATCG
CCCTTCGATCTCGTGGGCCACTTTGGCGACGGCGCGGCGCTCTTCGATCCAGCGCTTGGG
CGAATCGTTCCCACCAACGAGCGCGGCGTGACGCTGGAGCCTCTCGAGTCGCGGGCTTAC
ACTCTCGTGTGGCCGATTCCACTGGCGCACGAAGCCTCGCAGCTCGGGAGCTTCGCCAAT
TTGGAGGACCCCCCCTGGAGTTTGTAA

>AT2G17950
MEPPQHQHHHHQADQESGNNNNNKSGSGGYTCRQTSTRWTPTTEQIKILKELYYNNAIRS
PTADQIQKITARLRQFGKIEGKNVFYWFQNHKARERQKKRFNGTNMTTPSSSPNSVMMAA
NDHYHPLLHHHHGVPMQRPANSVNVKLNQDHHLYHHNKPYPSFNNGNLNHASSGTECGVV
NASNGYMSSHVYGSMEQDCSMNYNNVGGGWANMDHHYSSAPYNFFDRAKPLFGLEGHQEE
EECGGDAYLEHRRTLPLFPMHGEDHINGGSGAIWKYGQSEVRPCASLELRLN
>AT3G11260
MSFSVKGRSLRGNNNGGTGTKCGRWNPTVEQLKILTDLFRAGLRTPTTDQIQKISTELSF
YGKIESKNVFYWFQNHKARERQKRRKISIDFDHHHHQPSTRDVFEISEEDCQEEEKVIET
LQLFPVNSFEDSNSKVDKMRARGNNQYREYIRETTTTSFSPYSSCGAEMEHPPPLDLRLS
FL

>AT5G05770
MSSRGFNIKARGLCNNNNGGGGTGAKCGRWNPTVEQVKLLTDLFKAGLRTPSTDQIQKIS
MELSFYGKIESKNVFYWFQNHKARERQKCRKISTVKFDHRQDTDLSKPRRDNVRRHQLPA
KVCKVEEKMIETLQLFPLSKVERVRANVTAASHNEYTREQAYTTAFSTFSTCGAEMEHSP
LDLRLSFL
>AT5G59340
MENEVNAGTASSSRWNPTKDQITLLENLYKEGIRTPSADQIQQITGRLRAYGHIEGKNVF
YWFQNHKARQRQKQKQERMAYFNRLLHKTSRFFYPPPCSNVGCVSPYYLQQASDHHMNQH
GSVYTNDLLHRNNVMIPSGGYEKRTVTQHQKQLSDIRTTAATRMPISPSSLRFDRFALRD
NCYAGEDINVNSSGRKTLPLFPLQPLNASNADGMGSSSFALGSDSPVDCSSDGAGREQPF
IDFFSGGSTSTRFDSNGNGL
>AT3G18010
MWTMGYNEGGADSFNGGRKLRPLIPRLTSCPTAAVNTNSDHRFNMAVVTMTAEQNKRELM
MLNSEPQHPPVMVSSRWNPTPDQLRVLEELYRQGTRTPSADHIQQITAQLRRYGKIEGKN
VFYWFQNHKARERQKRRRQMETGHEETVLSTASLVSNHGFDKKDPPGYKVEQVKNWICSV
GCDTQPEKPSRDYHLEEPANIRVEHNARCGGDERRSFLGINTTWQMMQLPPSFYSSSHHH
HQRNLILNSPTVSSNMSNSNNAVSASKDTVTVSPVFLRTREATNTETCHRNGDDNKDQEQ
HEDCSNGELDHQEQTLELFPLRKEGFCSDGEKDKNISGIHCFYEFLPLKN
>AT2G28610
MSPVASTRWCPTPEQLMILEEMYRSGIRTPNAVQIQQITAHLAFYGRIEGKNVFYWFQNH
KARDRQKLRKKLAKQLHQQQHQLQLQLQQIKPKPISSMISQPVNKNIIDHHNPYHHHHHN
HHHNHHRPYDHMSFDCCSHPSPMCLPHQGTGVGEAPSKVMNEYYCTKSGAEEILMQKSIT
GPNSSYGRDWMMMMDMGPRPSYPSSSSSPISCCNMMMSSPKIPLKTLELFPISSINSKQD
STKL
>AT2G01500
MGYISNNNLINYLPLSTTQPPLLLTHCDINGNDHHQLITASSGEHDIDERKNNIPAAATL
RWNPTPEQITTLEELYRSGTRTPTTEQIQQIASKLRKYGRIEGKNVFYWFQNHKARERLK
RRRREGGAIIKPHKDVKDSSSGGHRVDQTKLCPSFPHTNRPQPQHELDPASYNKDNNANN
EDHGTTEESDQRASEVGKYATWRNLVTWSITQQPEEINIDENVNGEEEETRDNRTLNLFP
VREYQEKTGRLIEKTKACNYCYYYEFMPLKN
>AT1G46480
MKVHEFSNGFSSSWDQHDSTSSLSLSCKRLRPLAPKLSGSPPSPPSSSSGVTSATFDLKN
FIRPDQTGPTKFEHKRDPPHQLETHPGGTRWNPTQEQIGILEMLYKGGMRTPNAQQIEHI
TLQLGKYGKIEGKNVFYWFQNHKARERQKQKRNNLISLSCQSSFTTTGVFNPSVTMKTRT
SSSLDIMREPMVEKEELVEENEYKRTCRSWGFENLEIENRRNKNSSTMATTFNKIIDNVT
LELFPLHPEGR
>AT4G35550
MMEWDNQLQPNNHHSSNLQGIDVNGGSGAGGGMYVKVMTDEQYETLRKQIAIYGTICERL
VEMHKTLTAQQDLAGGRMGGLYADPMMSSLGHKMTARQRWTPTPVQLQILERIFDQGTGT
PSKQKIKDITEELSQHGQIAEQNVYNWFQNRRARSKRKQHGGGSSGNNNGESEVETEVEA
LNEKRVVRPESLLGLPDGNSNNNGLGTTTATTTAPRPEDLCFQSPEISSDLHLLDVLSNP
RDEHLVGKMGLAESYNLYDHVEDYGMSG
>AT1G20710
MEQESLNGRYGSRVMTDEQMETLRKQIAIYAVLCDQLVFLHNSLSSVPLLSSGMNPMRGE
YFDPMVASSSAHGMSTRPRWTPTTTQLQILENIYKEGSGTPNPRRIKEITMELSEHGQIM
EKNVYHWFQNRRARSKRKQPPTTTITSSQADDAAVTTTEERGRCGDDSGGFESYEHILFP
SPDLGIEHLLNRDKFID
>AT1G20700
MVKKKKEKEKSKEIEEMDREIQNGAYSGRVMTEEQMEILRKQIAVYAVICDQLVLLHNSL
SSYHPLSSGVRPMVGGYFDPMGASSSSHRISTRHRWTPTSTQLQILESIYDEGSGTPNRR
RIREIATELSEHGQITETNVYNWFQNRRARSKRKQPQTTTANGQADDVAVTTEERRSCGD
SGGLESYEHILFPSPDLGIEHLLSIGKFMET
>AT2G33880
MASSNRHWPSMFKSKPHPHQWQHDINSPLLPSASHRSSPFSSGCEVERSPEPKPRWNPKP
EQIRILEAIFNSGMVNPPREEIRRIRAQLQEYGQVGDANVFYWFQNRKSRSKHKLRLLHN
HSKHSLPQTQPQPQPQPSASSSSSSSSSSSKSTKPRKSKNKNNTNLSLGGSQMMGMFPPE
PAFLFPVSTVGGFEGITVSSQLGFLSGDMIEQQKPAPTCTGLLLSEIMNGSVSYGTHHQQ

HLSEKEVEEMRMKMLQQPQTQICYATTNHQIASYNNNNNNNNIMLHIPPTTSTATTITTS
HSLATVPSTSDQLQVQADARIRVFINEMELEVSSGPFNVRDAFGEEVVLINSAGQPIVTD
EYGVALHPLQHGASYYLI
>AT5G45980
MSSSNKNWPSMFKSKPCNNNHHHQHEIDTPSYMHYSNCNLSSSFSSDRIPDPKPRWNPKP
EQIRILESIFNSGTINPPREEIQRIRIRLQEYGQIGDANVFYWFQNRKSRAKHKLRVHHK
SPKMSKKDKTVIPSTDADHCFGFVNQETGLYPVQNNELVVTEPAGFLFPVHNDPSAAQSA
FGFGDFVVPVVTEEGMAFSTVNNGVNLETNENFDKIPAINLYGGDGNGGGNCFPPLTVPL
TINQSQEKRDVGLSGGEDVGDNVYPVRMTVFINEMPIEVVSGLFNVKAAFGNDAVLINSF
GQPILTDEFGVTYQPLQNGAIYYLI
>AT3G03660
MDQEQTPHSPTRHSRSPPSSASGSTSAEPVRSRWSPKPEQILILESIFHSGMVNPPKEET
VRIRKMLEKFGAVGDANVFYWFQNRRSRSRRRQRQLQAAAAAAAATTNTCDQTMMVSNSL
PHHSGSDLGFGGCSTSSNYLFASSSSSYGGGCDNQSNSGMENLLTMSGQMSYHEATHHHY
QNHSSNVTSILCPSDQNSNFHYQQGAITVFINGVPTEVTRGGIDMKATFGEDLVLVHSSG
VPLPTDEFGFLMHSLQHGEAYFLVPRQT
>AT5G17810
MNQEGASHSPSSTSTEPVRARWSPKPEQILILESIFNSGTVNPPKDETVRIRKMLEKFGA
VGDANVFYWFQNRRSRSRRRHRQLLAATTAAATSIGAEDHQHMTAMSMHQYPCSNNEIDL
GFGSCSNLSANYFLNGSSSSQIPSFFLGLSSSSGGCENNNGMENLFKMYGHESDHNHQQQ
HHSSNAASVLNPSDQNSNSQYEQEGFMTVFINGVPMEVTKGAIDMKTMFGDDSVLLHSSG
LPLPTDEFGFLMHSLQHGQTYFLVPRQT

>OS04G55590
MRLHHLHVAYLDHKASSSSSSPAPPSISPSSIPGSAAFPAFSFKCLRPLAPKISLPEPRK
MIAPPDFVVPRARNASKLLNYTVQVPAAGTTRWNPSAEQIKVLEMLYRGGMRTPNSVQIE
RITEELGKYGRIEGKNVFYWFQNHKARERQKQKRAALLTLSTLDPSLLPATANETKEAPE
KKEKDVEDGLASCKRRCKAWGDGAGDGDAVVATEAAGGCTDEVTLELFPLHPQGKA
>OS04G56780
MDHMQQQQRQQVGGGGGEEVAGRGGVPVCRPSGTRWTPTTEQIKILRELYYSCGIRSPNS
EQIQRIAAMLRQYGRIEGKNVFYWFQNHKARERQKKRLTTLDVTTTTAAAADADASHLAV
LSLSPTAAGATAPSFPGFYVGNGGAVQTDQANVVNWDCTAMAAEKTFLQDYMGVSGVGCA
AGAAPTPWAMTTTTREPETLPLFPVVFVGGDGAHRHAVHGGFPSNFQRWGSAAATSYTIT
VQQHLQQHNFYSSSSSQLHSQDGPAAGTSLELTLSSYYCSCSPYPAGSM
>OS11G01130
MPQTPSTRWCPTPEQLMILEEMYRSGVRTPNAAEIQQITAHLAYYGRIEGKNVFYWFQNH
KARERQRLRRRLCARHQQQPSPPSSTVPPAPTAAAAGAVVQVHPAVMQLHHHHHHHHPYA
AAAAAQSHHLQQQQQQQAEWPAAVDYCSTASASASATAADMAIPPCCRPLKTLELFPTKS
TSGGLKEDCCSSSKSSSCSTSTN
>OS05G02730
MAPAVQQQQSGGGGGSTGAAAVGSTTRWCPTPEQLMMLEEMYRGGLRTPNAAQIQQITAH
LSTYGRIEGKNVFYWFQNHKARDRQNAAAASASPTTSSPAPTTTTTTSPPPPPSFRRRSF
CRRCTPPPPPPPAAGGGGVVLPAAEAIGRSSSSSDYSLGKLVDNFGVALEETFPAQPQQP
ATTMAMTAVVDTTAVAAAAGGFCRPLKTLDLFPGGLKEEQHDVV >OS01G63510
MEALSGRVGVKCGRWNPTAEQVKVLTELFRAGLRTPSTEQIQRISTHLSAFGKVESKNVF
YWFQNHKARERHHHKKRRRGASSPDSGSNDDDGRAAAHEGDADLVLQPPESKREARSYGH
HHRLMTCYVRDVVETEAMWERPTREVETLELFPLKSYDLEVDKVRYVRGGGGEQCREISF
FDVAAGRDPPLELRLCSFGL
>OS01G62310
METTTTTLGGGGGGRAGGFSDPPSPLSPPLSPASAAAAALANARWTPTKEQIAVLEGLYR
QGLRTPTAEQIQQITARLREHGHIEGKNVFYWFQNHKARQRQKQKQQSFDYFSKLFRRPP
PLPVLHRPLARPFPLAMAPTAMPPPPPPPPATTTTAACNAGGVMFRTPSFMPVATNNASYY
PQQQTPLLYPGMEVCPHDKSTAQPPATTTMYLQAPPSSAHLAAAAGRGAAEAEGHGRRGG
GAGGRETLQLFPLQPTFVLPDHKPLRAGSACAAVSPTTPSASASFSWESESSDSPSSEAP
PFYDFFGVHSGGR
>OS01G60270
MEWDKAKASSGEAVDDRGGGEGGLGYVKVMTDEQMEVLRKQISIYATICEQLVEMHRALT

AQQDSIAGMRLGNLYCDPLMVPGGHKITARQRWTPTPMQLQILENIFDQGNGTPSKQKIK
DITAELSQHGQISETNVYNWFQNRRARSKRKQAALPNNNAESEAEADEESPTDKKPKSDR
PLHQNIAMRDHNSERISEMHHFDTEHEQIRRMMYASNDSSSRSSGSLGQMSFYDNVMSNP
RIDHFLGKVESPGSFPHMRSGESFDMY
>OS01G47710
MASSNRHWPSMFRSKHATQPWQTQPDMAGSPPSLLSGSSAGSAGGGGYSLKSSPFSSVGE
ERVPDPKPRWNPRPEQIRILEAIFNSGMVNPPRDEIPRIRMQLQEYGQVGDANVFYWFQN
RKSRSKNKLRSGGTGRAGLGLGGNRASAPAAAHREAVAPSFTPPPPILPAPQPVQPQQQL
VSPVAAPTSSSSSSSDRSSGSSKPARATSTQAMSVTTAMDLLSPLAAACHQQMLYQGQPL
ESPPAPAPKVHGIVPHDEPVFLQWPQSPCLSAVDLGAAILGGQYMHLPVPAPQPPSSPGA
AGMFWGLCNDVQAPNNTGHKSCAWSAGLGQHWCGSADQLGLGKSSAASIATVSRPEEAHD
VDATKHGLLQYGFGITTPQVHVDVTSSAAGVLPPVPSSPSPPNAAVTVASVAATASLTDF
AASAISAGAVANNQFQGLADFGLVAGACSGAGAAAAAAAPEAGSSVAAVVCVSVAGAAPP
LFYPAAHFNVRHYGDEAELLRYRGGSRTEPVPVDESGVTVEPLQQGAVYIVVM
>OS07G34880
MMALGVPPPPSRAYVSGPLRDDDTFGGDRVRRRRRWLKEQCPAIIVHGGGRRGGVGHRAL
AAGVSKMRLPALNAATHRIPSTSPLSIPQTLTITRDPPYPMLPRSHGHRTGGGGFSLKSS
PFSSVGEERVPDPKPRRNPRPEQIRILEAIFNSGMVNPPRDEIPRIRMQLQEYGQVGDAN
VFYWFQNRKSRSKNKLRSGGTGRAGLGLGGNRASEPPAAATAHREAVAPSFTPPPILPPQ
PVQPQQQLVSPVAAPTSLSSSSSDRSSGSSKPARATLTQAMSVTAAMDLLSPLRRSARPR
QEQRHV
>OS05G48990
MASPNRHWPSMFRSNLACNIQQQQQPDMNGNGSSSSSFLLSPPTAATTGNGKPSLLSSGC
EEGTRNPEPKPRWNPRPEQIRILEGIFNSGMVNPPRDEIRRIRLQLQEYGQVGDANVFYW
FQNRKSRTKNKLRAAGHHHHHGRAAALPRASAPPSTNIVLPSAAAAAPLTPPRRHLLAAT
SSSSSSSDRSSGSSKSVKPAAAALLTSAAIDLFSPAPAPTTQLPACQLYYHSHPTPLARD
DQLITSPESSSLLLQWPASQYMPATELGGVLGSSSHTQTPAAITTHPSTISPSVLLGLCN
EALGQHQQETMDDMMITCSNPSKVFDHHSMDDMSCTDAVSAVNRDDEKARLGLLHYGIGV
TAAANPAPHHHHHHHHLASPVHDAVSAADASTAAMILPFTTTAAATPSNVVATSSALADQ
LQGLLDAGLLQGGAAPPPPSATVVAVSRDDETMCTKTTSYSFPATMHLNVKMFGEAAVLV
RYSGEPVLVDDSGVTVEPLQQGATYYVLVSEEAVH
>OS07G48560
MDGGHSPDRHAAAAAGEPVRSRWTPKPEQILILESIFNSGMVNPPKDETVRIRKLLERFG
AVGDANVFYWFQNRRSRSRRRQRQLQAQAQAAAAAASSGSPPTASSGGLAPGHAGSPASS
LGMFAHGAAGYSSSSSSSWPSSPPSVGMMMGDVDYGGGGDDLFAISRQMGYMDGGGGSSS
SAAAGQHQQQQLYYSCQPATMTVFINGVATEVPRGPIDLRSMFGQDVMLVHSTGALLPAN
EYGILLHSLQMGESYFLVTRSS
>OS03G20910
MEGSSNSPDRQSSGGSPPEERGGGGSGGGGGRSAAGEPVRSRWTPKPEQILILESIFNSG
MVNPPKDETVRIRKLLERFGAVGDANVFYWFQNRRSRSRRRQRQMQAAAAAAAAAASSSS
PSANTSPAAASAATVQVGLPPGAVVHTMAMGGSACQYEQQASSSSSSGSTGGSSLGLFAH
GAGASGAGGYLQASCGASASASSALAPGLMGDVVDSGGSDDLFAISRQMGFVGSPRCSPA
SSPATPSSAATAAQQQFYSCQLPAATITVFINGVPMEMPRGPIDLRAMFGQDVMLVHSTG
ALLPVNDYGILMQSLQIGESYFLVARPP
>OS08G14400
MDRTATASWEVMSRRGEQQQQLMMQAPASHNGGSGGGEPARSRWAPKPEQILILESIFNS
GMVNPAKDETARIRRLLERFGAVRDANVFYWFQNRRSRSRRRARQLQQACGAALHQLPSA
AAAAGAGGGGDYYHHHHQPSSSPFLMHGGGGGGVVTSTTAAPAVAASGHFLADEVDGGGD
DDLFAISRQMGLMARHGGGDHHYSSYADSDATQLSYQPTGTIQVFINGVAYDVPSGGALD
MAGTFGRDAMLVHSSGEVLPVDEHGVLINSLQMGECYYLVSKSI

>PpaWOX02_5
MESESRLGRMMDMTPLGSGLQGQPVPGGAALGLGPSLENSLPQPMYTRGSGQVMTEEQLE
TLRRQISVYATICQQLVEMHKASVSQQASLPGILASGQIVSMDHLTGTPPHKSTARQRWT
PSQHQLQILEKLFEQGSGTPNKQRIKEITAELSQHGAISETNVYNWFQNRKARAKRKQQL
VTPRDGESEADTDVESPKEKRTRQEGEQNQDESGGVGDTNGGGNSDGAGNGVPEQRAANF
DQQDAASSALLHSQTDTKPDISSFNRSAGFDPHNVSQGIPPMMS

>PpaWOX01
MQSNGMEPESRLGRMMDMSPIGSGLRGQPVSGGAMLGVGPSLENPLPQPMYTRGSGQVMT
EEQLETLRRQISVYATICQQLVEMHKASVSQQASLPGILAGGQIVSMDQMTGTPTHKSTA
RQRWTPSQHQLQILEKLFEQGSGTPNKVRIKEITAELSQHGAISETNVYNWFQNRKARAK
RKQQLVTPKDGESEADSDGESPKEKRTRQKGDQNQDESVGIGDPHGGGSFDGAGNGVPEQ
KAVNSDQQDATSSALLHPQADTKPDISSFNRGAGFDPYNMPQGIPPMMS
>PpaWOX03
MACEQRASTNPPTRSRWSANQQQLQNLESIFEQGNGNTPNKARIKDITIELNQFGHISET
NVYNWFQNRKARAKRKLQQRWRVNQMRSGEAEISGDVQPYLPDAKRLKAVGPQSQSATGS
AATPASALDAGSSIAAAEIDTELRSRPALMPLTSITPLTMPLTVTRVEAIEETLGGGAEH
WHDQGLPVLLPPSLHGETSFIDYYRFRK

>Smo404134
MDSLPTASCSTPSVGRRSYGRRWIANEVTWLELNCHLLKGRSEVFICGVFTGSPQRSFSL
LFSPPASLDRMPRRKQQASRASSVSDQIDQERREMRLEIKALHVHHRSQRNNTNISSEPA
PPRPVGVQQRWEPNSYQLQILEEFYAKATPPSPENIANIAELVGQVDHSKVYYWFSNKKS
REKRKRRRLEEAAASASSSPAFSAIATTAAASSGSEQSHHQSYADQIVNDGSFLPDACLF
ETNVTLEQPRIIPSHEEESPLQLSLGWSFANLDEHWEFSVLIDRTLRLSPLGKWKLDDYR
NVILSSTFWPKI
>Smo407131
MPRSKQQASRASSVSDQIDQERREMRLEIKALHVHHRSQRNNINISSEPAPPRPVGVQQR
WEPNSYQLQILEEFYAKATPPSPENIANIAELVGQVDHSKVYYWFSNKKSREKRKRRRLE
EAGASASSSPAFSAIATTAAASSGSEQSHHQSYADQIVNDGSFLPDACLFETNVTLEQPR
IVIRLWPWSSSSIVALAVDLDIDNMQQASKAAIIFATDDLHVFACTVGKWKLDDYRNVLL
SSTFWPKI
>Smo417553
MRRCEQQAASSPASSESDQIDQERDINTELLELKALHMHHRSRRKHIEISSVPAPPAPTR
PAQQRWRPNSQQLGILEEFYAKGTPPSQENVTEIAELIGHHGPVDESKVYYWFQNKKSRE
KRKRRRIEEANAASGASASSSPAAASATAAFQQDLSSSAPSASASASSGSEQLHHPAAQS
YAAAQIVIDGGVVSIIDDKSPFDLVGHFGDGAALFDPALGRIVPTNERGVTLEPLESRAY
TLVWPIPLAHEASQLGSFANLEDPPWSL

>OT13G01350
MGSSDARTDDANGLESGKSAVERRAAMVHAAKATLPGDVRAPPPGAAVHMTERQLYAFRQ
QIAAYAHICQQLLQITTVSATQQTPRQREMRHEVPGATGANWGASVPVTAPASAPLSSSN
HSSGKTRGEDKQARGPRWTGTPTQYEILEDLFQKGEQPPVRDRLTELTEMLKQHGPVQES
NVYNWFQNRRSREKKLQAMAMGH

Table 1: Gene and protein sequences of the WOX family in the model genomes *A. thaliana*, *O. sativa*, *P. patens* and *O. tauri*

		Model genome WOX database		WOX13 OG database		GenBank NR database	
Motif	Length	score drop	hits above the drop	score drop	hits above the drop	score drop	hits above the drop
Homeodomain WOX	60	**118.5**	32	**113.5**	33	**92.1**	92
WOX1 OG	10	12.5	14	a	a	c	c
WUS	8	8.3	2	a	a	c	c
WOX13 OG	39	**73.1**	7	**51.5**	33	**53**	9
LqxG-WOX8 OG	30	**69.1**	9	a	a	**49.5**	10
VFIN-WOX8 OG	30	**84.5**	7	a	a	**53.8**	9
YxDPL-WOX13 OG	12	27.3	2	8.8	28	7.9	4
ESExE-WOX13 OG	16	19.3	4	15.4	29	6.3	6
Monocotyledon-WOX OG	22	44.3	14	39.4	3	26.3	10
QadDaAVTT-WOX10 OG	22	**52.1**	2	**51.3**	3	22.6	2
YfdPM-WOX10 OG	17	35.3	2	22.6	3	26.2	2
LxxGQ-gymnosperm-moss-WOX OG	22	**51.8**	2	**51.1**	4	b	b
QdxxxxLL-gymnosperm-moss-WOX OG	41	**98.9**	2	**97.3**	4	b	b

Table 2: Specificity and sensitivity of HMM for WOX motifs

Scaffold	homeodomain WOX	WOX1 OG	WUS	WOX13 OG	LQxG WOX8 OG	VFIN WOX8 OG	YxDPL WOX13 OG	ESExE WOX13OG	QAdDaAVTT WOX10 OG	YFdPM WOX10 OG	
AM429035.2	153.6	11.8	-	-	-	-	-	-	a	a	WOX1 OG
AM435207.1	127.0	-	-	-	59.3	66.6	-	-	a	a	WOX8 OG
AM439847.2	147.7	12.2	-	-	-	-	-	-	a	a	WOX1 OG
AM447418.2	139.6	12.7	8.3	-	-	-	-	-	a	a	WUS
AM447494.2	152.4	11.8	-	-	-	-	-	-	a	a	WOX1 OG
AM463144.2	143.9	12.3	-	-	-	-	-	-	a	a	WOX1 OG
AM463736.1	136.0	-	-	74.0	-	-	27.7	24.1	a	a	WOX13 OG
AM473516.1	108.2	-	-	74.8	-	-	17.4*	19.1	a	a	WOX13 OG
AM486367.2	135.9	-	-	72.3	-	-	25.4	24.3	a	a	WOX13 OG
AM488026.2	139.9	-	-	-	51.9	45.7	-	-	a	a	WOX8 OG
AM488389.1	135.0	10.5	-	-	-	-	-	-	a	a	WOX1 OG
contig_1094	143.9	12.4	-	-	-	-	-	-	a	a	WOX1 OG
contig_17869	135.9	-	-	72.3	-	-	25.4	24.3	a	a	WOX13 OG
contig_2321	145.2	10.5	-	-	-	-	-	-	a	a	WOX1 OG
contig_2350	153.6	11.8	-	-	-	-	-	-	a	a	WOX1 OG
contig_24189	129.5	-	-	74.8	-	-	27.7	19.1	a	a	WOX13 OG
contig_24189	136.0	-	-	74.0	-	-	27.7	24.1	a	a	WOX13 OG
contig_29485	127.0	-	-	-	59.3	66.6	-	-	a	a	WOX8 OG
contig_4115	146.0	11.7	-	-	-	-	-	-	a	a	WOX1 OG
contig_648	152.4	11.8	-	-	-	-	-	-	a	a	WOX1 OG
contig_8271	147.7	12.2	-	-	-	-	-	-	a	a	WOX1 OG
contig_9279	139.9	-	-	-	51.9	45.7	-	-	a	a	WOX8 OG

Table 3: Vitis vinifera **WOX protein prediction with HMM WOX motifs**
