# Supplementary material for: Genes of the most conserved WOX clade in plants affect root and flower development in Arabidopsis
Source: BMC Evol Biol. 2008 Oct 24;8:291. doi: 10.1186/1471-2148-8-291 (PMC2584047; doi:10.1186/1471-2148-8-291)
Supplement: Additional file 4 — Expression analyses. Figure 1 : Real-time PCR analysis of Ostreoccocus tauri gene expression in a 12-hour light/12-hour dark synchronized cell culture. Copy number of WOX, KNOX, Histone4 and CyclinB RNA was quantified using total RNA sampling from the starting of the light period (0 h) or from continuous light grown cell culture. Figure 2 : Alignments of predicted sequences of AtWOX13 (A) and AtWOX14 (B) chimeric proteins with the full length ones. The homeodomain are indicated in bold and the amino acid changes at the end of the chimeric proteins indicated in lower case. Figure 3 : AtWOX13 expression is correlated with the induction of cell proliferation. Data mining analysis of the expression profiles of a set of genes in protoplast culture of Arabidopsis thaliana. (A) AtWOX13 (-◆-), ATHB-7 (■), PCNA1 (▲); (B) Expression profile of a cluster of cell cycle genes including AT4G37490 (CYC1); AT1G44110 (CYCA1;1 (■)); AT5G11300 (CYC3B); AT1G47210 (CYCA3;2); AT3G05330 (CDK); AT2G26760 (CYCB1;4); AT4G35620 (CYCB2;2); AT2G26430 (RCY1); AT2G31400 (ATPDNA Binding). Table 1: Primer sequences designed for expression analysis, genotyping (g primers) and cDNA cloning (2 last row) using primer3 software. [file 1471-2148-8-291-S4.pdf]

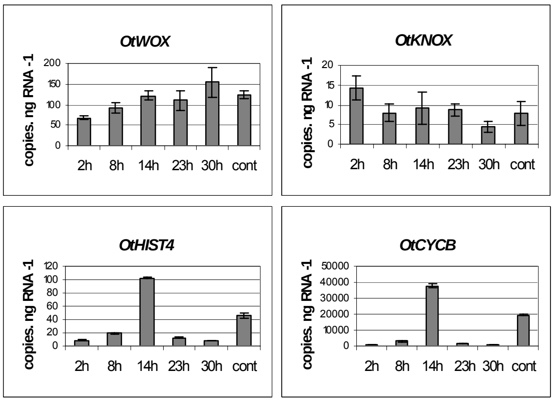

Figure 1: Real-time PCR analysis of *Ostreoccocus tauri* gene expression in a 12-hour light/12-hour dark synchronized cell culture.

A

```
             1                                                    50
 WOX13ΔCt    MMEWDNQLQP NNHHSSNLQG IDVNGGSGAG GGMYVKVMTD EQYETLRKQI
    WOX13    MMEWDNQLQP NNHHSSNLQG IDVNGGSGAG GGMYVKVMTD EQYETLRKQI

             51                                                  100
 WOX13ΔCt    AIYGTICERL VEMHKTLTAQ QDLAGGRMGG LYADPMMSSL GHKMTARQRW
    WOX13    AIYGTICERL VEMHKTLTAQ QDLAGGRMGG LYADPMMSSL GHKMTARQRW

             101                                                 150
 WOX13ΔCt    TPTPVQLQIL ERIFDQGTGT PSKQKIKDIT EELSQHGQIA EptgYmpt..
    WOX13    TPTPVQLQIL ERIFDQGTGT PSKQKIKDIT EELSQHGQIA EQNVYNWFQN

             151                                                 200
 WOX13ΔCt    .......... .......... .......... .......... ..........
    WOX13    RRARSKRKQH GGGSSGNNNG ESEVETEVEA LNEKRVVRPE SLLGLPDGNS

             201                                                 250
 WOX13ΔCt    .......... .......... .......... .......... ..........
    WOX13    NNNGLGTTTA TTTAPRPEDL CFQSPEISSD LHLLDVLSNP RDEHLVGKMG

             251             268
 WOX13ΔCt    .......... ........
    WOX13    LAESYNLYDH VEDYGMSG
Consensus    .......... ........
```

B

```
             1                                                    50
 WOX14dΔt    MVKKKKEKEK SKEIEEMDRE IQNGAYSGRV MTEEQMEILR KQIAVYAVIC
    WOX14    MVKKKKEKEK SKEIEEMDRE IQNGAYSGRV MTEEQMEILR KQIAVYAVIC

             51                                                  100
 WOX14ΔCt    DQLVLLHNSL SSYHPLSSGt llsVfvpd.. .......... ..........
    WOX14    DQLVLLHNSL SSYHPLSSGV RPMVGGYFDP MGASSSSHRI STRHRWTPTS

             101                                                 150
 WOX14ΔCt    .......... .......... .......... .......... ..........
    WOX14    TQLQILESIY DEGSGTPNRR RIREIATELS EHGQITETNV YNWFQNRRAR

             151                                                 200
 WOX14ΔCt    .......... .......... .......... .......... ..........
    WOX14    SKRKQPQTTT ANGQADDVAV TTEERRSCGD SGGLESYEHI LFPSPDLGIE

             201      211
 WOX14ΔCt    .......... .
    WOX14    HLLSIGKFME T
```

Figure 2: Alignments of predicted sequences of AtWOX13 (A) and AtWOX14 (B) chimeric proteins with the full length ones.

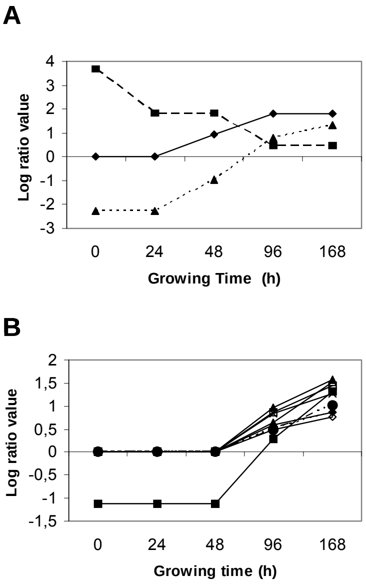

Figure 3: *AtWOX13* expression is correlated with the induction of cell proliferation.

Forward primers	Reverse primers	
5′-AACGAAAGCAACCTCCGACA-3′	5′-CCCTCCAGAATCATCTCCACAC-3′	*AtWOX10*
5′-AAGATGGGACTGGCGGAAA-3′	5′-ACTGCTTATGACTGACTACCAAATCC-3′	*AtWOX13*
5′-CCAAACGAAAGCAGCCTCAA-3′	5′-CCCCTGAATCTCCACAACTCC-3′	*AtWOX14*
5′-TTAATGGTTGGCAAGGGACTAC-3′	5′-GCTTCAAAACCACACGATGA-3′	*PpaWOX01*
5′-CTGCGGGAGATTGTAGTTTGC-3′	5′-CGTTGCTCTGCCACCAAGA-3′	*PpaWOX02a*
5′-GATGCCGTTTGAGGAAAGAGAC-3′	5′-AACAACACACCGCCCTTCAG-3′	*PpaWOX02b*
5′-GATTACGCCGCTGACGATG-3′	5′-TAAAGAAGGGGGCAGGAGGA-3′	PpaWOX03
5′-TTTCAGCACACTCCCTTCCC-3′	5′-AACCATAGTCATCTGCGAAATAAACC-3′	*PpaACTIN*
5′-GCCGACGCAGTACGAGATTT-3′	5′-ACCGGACCGTGTTGCTTG-3′	*OtWOX*
5′-CCGACTGTTTGAACGAGGATG-3′	5′-TCCTTCGCCTTCTCCAATGA-3′	*OtKNOX*
5′-CTGCGTGGGCAAGTCAAAGT-3′	5′-GGCTCGTCCCGTGTGTTG-3′	*OtRH4*
5′-TCGTGGCGAGCAAGTATGAA-3′	5′-CGTCGGAACAGTGAGATGGAA-3′	*OtCYCLINB*
5′-TTCGCAAGGGTGTGGAGATT-3′	5′-CGCCTCGTCTTCAGGTTTCA-3′	*OtRH21*
5′-CCGTGACCTACACCGAGCA-3′	5′-CGCCTCTTCGTCCCATCA-3′	*OtHISTONE4*
5′-CGTGTGCCAGGTGCCCACGGAATAGT-3′	5′-TTGCTGTTGTTGTCCCTAACC-3′	*gAtWOX13*
	5′-ACAGAGGTGGACTCCTACGC-3′	*gTDNAwox13*
5′-TACCTCGGGTTCGAAATCG AT-3′	5′-TGAAAGGCCATAGTTCTCCAA-3′	*gAtWOX14*
	5′-GCTCATCAAATGGTCTCTTGTAAA-3′	*gDS-wox14*
5′-TCTCGAGTGGAGCTTTTGCAGGTCTCT-3′	5′-AGGATCCTCAG AATTTCGCTCAGAAGAT-TT-3′	*Xba*I/*BamH*I WOX13
5′-GACAGCGGCCGCGGGGTTGTGAGTCCTAT-TGC3′	5′-GACACTAGTTGAACAAGACAATGAGAAA-GTGAA-3	NotI/*Spe*I WOX14

Table 1: Primer sequences
